# Catalytic Diastereo- and Enantioselective Vinylogous Mannich Reaction of Alkylidenepyrazolones to Isatin-Derived Ketimines

**DOI:** 10.1021/acs.orglett.1c02571

**Published:** 2021-09-23

**Authors:** Laura Carceller-Ferrer, Carlos Vila, Gonzalo Blay, M. Carmen Muñoz, José R. Pedro

**Affiliations:** † Departament de Química Orgànica, Facultat de Química, Universitat de València, Dr. Moliner 50, 46100 Burjassot, València, Spain; ‡ Departament de Física Aplicada, Universitat Politècnica de València, Camino de Vera s/n, 46022 València, Spain

## Abstract

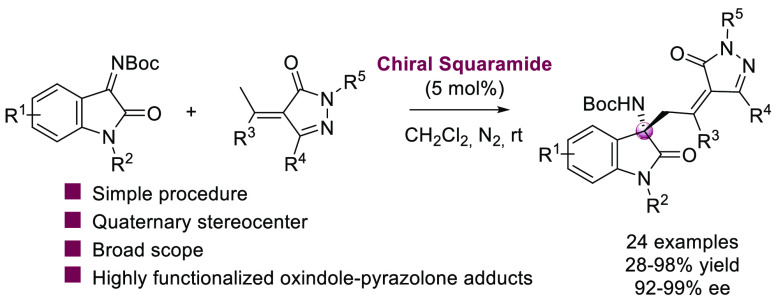

A valuable organocatalytic vinylogous Mannich reaction between alkylidenepyrazolones and isatin-derived ketimines has been successfully established. Squaramide organocatalyst, prepared from quinine, catalyzed the diastereo- and enantioselective vinylogous Mannich addition, affording a range of aminooxindole-pyrazolone adducts (24 examples) with excellent outcomes: up to 98% yield with complete diastereoselectivity and excellent enantioselectivity (up to 99% ee). Additionally, different synthetic transformations were performed with the chiral pyrazolone-oxindole adducts.

The direct catalytic asymmetric vinylogous reaction represents a powerful tool in synthetic organic chemistry to introduce stereocenters at the γ-position or even more remote positions of the functional groups in organic compounds in an atom-economical and efficient way.^1^ In this research area, the asymmetric vinylogous Mannich reaction is a powerful, direct, and straightforward C–C bond-forming reaction leading to the synthesis of optically active δ-amino-α,β-unsaturated carbonyl derivatives.^1d^ This class of compounds is a significant building block for the synthesis of biologically active compounds and drugs.

On the contrary, pyrazolone derivatives represent one of the most important five-membered heterocycles containing nitrogen atoms, which are present in several bioactive natural products and pharmaceuticals.^2^ Therefore, the enantioselective synthesis of chiral pyrazolones has received the attention of the synthetic organic chemists in the last several years.^3^ In this context, the asymmetric vinylogous nucleophilic γ-addition of α,β-unsaturated pyrazolone bearing γ-hydrogen atoms to electrophiles has been explored for the construction of chiral pyrazolones. However, these examples are restricted to the use of α,β-unsaturated compounds^4^ or Morita–Baylis–Hillman carbonates^5^ as electrophiles. As far as we know, the corresponding asymmetric nucleophilic 1,2-addition of alkylidenepyrazolones to carbonyl compounds or imines has not yet been described in the literature (Scheme 1A).

**Scheme 1:**
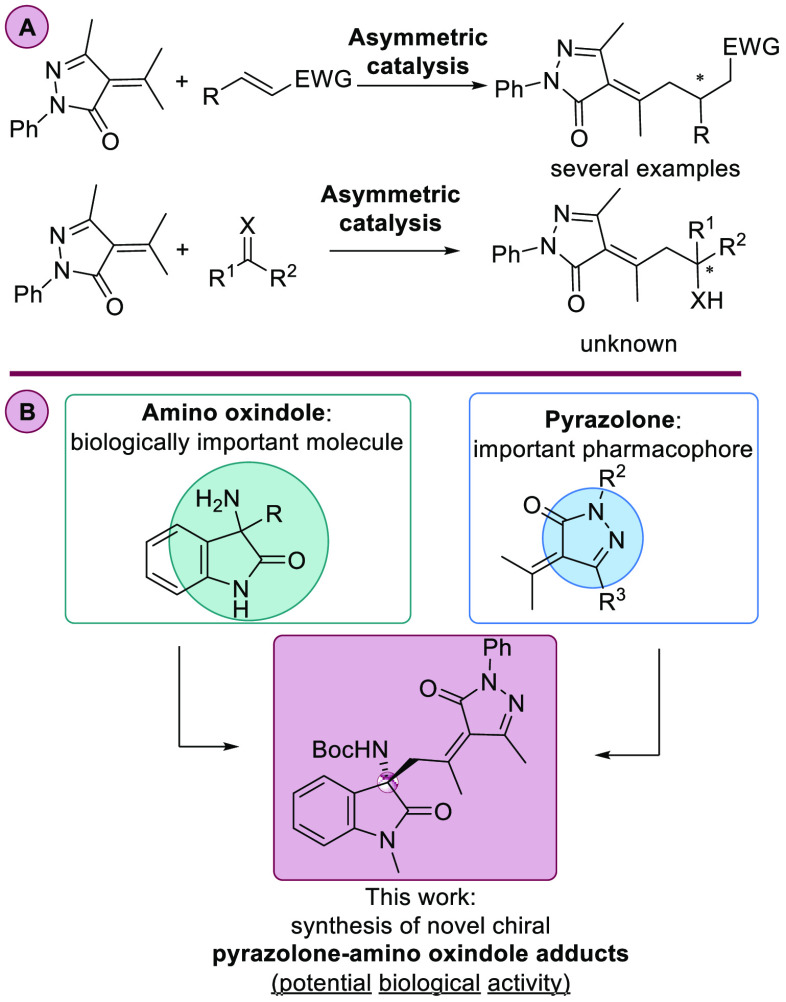
Asymmetric Vinylogous Alkylation of Alkylidenepyrazolones

During our recent studies on the enantioselective Mannich addition of pyrazolones to imines,^6^ we envisioned that the corresponding asymmetric vinylogous Mannich reaction could be feasible. Using α-isopropylidenepyrazolone as a nucleophile, remote γ-exocyclic functionalization of the diazaheterocycle could be possible using isatin-derived ketimines as electrophiles and bifunctional organocatalysis. The nucleophilic addition to isatin-derived ketimines constitutes a straightforward methodology to synthesize enantioenriched amino oxindole compounds.^7^ Numerous natural products and pharmacologically active compounds contain in their structures the amino-oxindole scaffold, showing the importance of this structural motif in synthetic organic chemistry.^8^ In light of the pharmacological and biological activities of pyrazolones and amino oxindoles, the combination of both structural motifs into one molecule could result in novel and interesting chiral alkylidenepyrazolones bearing a quaternary aminooxindole stereocenter that may be useful for drug discovery (Scheme 1B).^9^

Initially, we selected the enantioselective vinylogous Mannich reaction of α-isopropylidenepyrazolone **2a**, which was easily prepared from the commercially available edaravone and acetone, and isatin-derived *N*-Boc ketimine **1a** in CH_2_Cl_2_ at room temperature. With these conditions, several bifunctional organocatalysts were tested, and the results are summarized in Table 1. We selected bifunctional organocatalysts^10^ with a tertiary amine responsible for the activation of the nucleophile (deprotonation of the γ-hydrogens of the α,β-unsaturated pyrazolone) and a hydrogen-bonding donor moiety with the purpose of activating the electrophile (the isatin-derived *N*-Boc ketimine). When quinine (**I**) was used as catalyst, the yield of the Mannich product **3aa** was very low (6%), but the enantioselectivity was moderate (50% ee). We observed large amounts of *N*-methylisatine from the hydrolysis of ketimine **1a**. Takemoto’s thiourea **II** and quinine-derived thiourea **III** exhibited high stereocontrol (90% ee); however, the yield of product **3aa** was still low (∼20%). Delightfully, quinine-derived squaramide **IV** gave excellent enantiomeric excess (98%), and the yield increased to 42% after 3 days of reaction (entry 4). When benzylic (**V**)- and *tert-*butyl (**VI**)-substituted squaramides were used as catalysts, product **3aa** was obtained in similar yield but with somewhat lower enantioselectivity. The squaramide **VII**, prepared from dihydroquinine, displayed similar reactivity and stereoselectivity, and product **3aa** was obtained in 41% yield with 97% *ee* after 3 days. Squaramide **VIII**, prepared from quinidine, gave a similar yield (39%) and enantiomeric excess (96% ee) as quinine-based **IV**, but the opposite enantiomer was obtained. We chose **IV** to continue the optimization process of the reaction conditions. Different solvents were tested, achieving high enantioselectivities but lower yields than CH_2_Cl_2_ (entries 9–13). To improve the yield of the reaction, we studied the variation of the equivalents of the electrophile (entry 14) or nucleophile (entry 15); however, the yields were lower. We observed in all cases the formation of *N*-methyl isatin, the corresponding hydrolysis product of **1a**. To avoid the hydrolysis of the ketimine and increase the yield, we performed the reaction under an anhydrous nitrogen atmosphere (entry 16). In this case, the yield of the Mannich product **3aa** increased to 57%, maintaining the enantioselectivity (98%). Finally, we increased the reaction scale to 0.2 mmol and obtained similar results (entry 17).

**Table 1:**
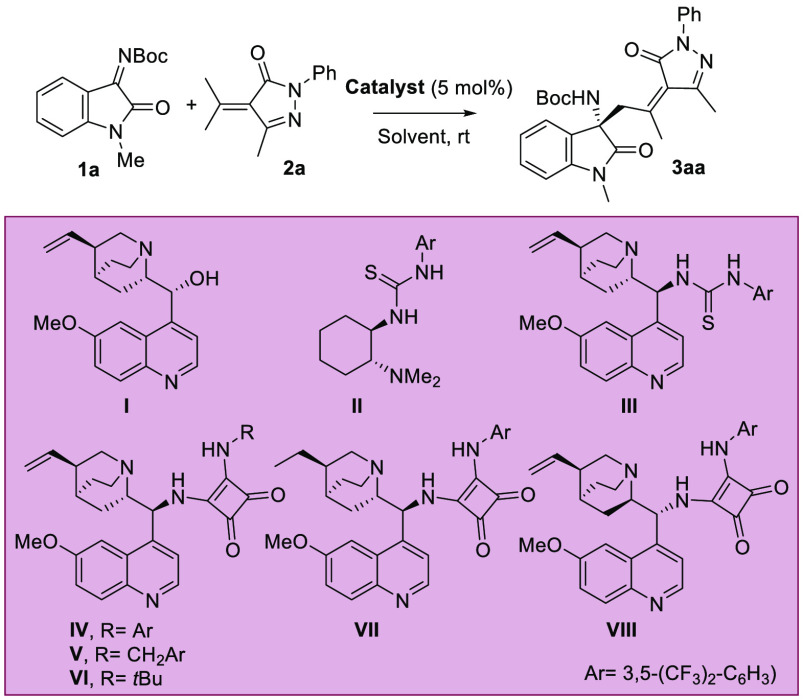
Optimization of the Reaction Conditions^a^

entry	catalyst	solvent	*t* (days)	yield (%)^b^	ee (%)^c^
1	**I** (5%)	CH_2_Cl_2_	4	6	50
2	**II** (5%)	CH_2_Cl_2_	4	16	91
3	**III** (5%)	CH_2_Cl_2_	3	19	89
4	**IV** (5%)	CH_2_Cl_2_	3	42	98
5	**V** (5%)	CH_2_Cl_2_	3	41	92
6	**VI** (5%)	CH_2_Cl_2_	3	44	94
7	**VII** (5%)	CH_2_Cl_2_	3	41	97
8	**VIII** (5%)	CH_2_Cl_2_	3	39	96^d^
9	**IV** (5%)	ClCH_2_CH_2_Cl	3	40	96
10	**IV** (5%)	CHCl_3_	3	28	97
11	**IV** (5%)	EtOAc	3	33	91
12	**IV** (5%)	Et_2_O	3	55	92
13	**IV** (5%)	toluene	3	42	95
14^e^	**IV** (5%)	CH_2_Cl_2_	3	38	97
15^f^	**IV** (5%)	CH_2_Cl_2_	3	42	97
16^g^	**IV** (5%)	CH_2_Cl_2_	3	57	98
17^g^^,^^h^	**IV** (5%)	CH_2_Cl_2_	3	52	98

a Reaction conditions: **1a** (0.1 mmol), **2a** (0.1 mmol), and 5 mol % of organocatalyst in 1 mL of solvent at rt under an air atmosphere.

b Isolated yield of **3aa** after column chromatography.

c Determined by chiral HPLC.

d Opposite enantiomer was obtained.

e 0.12 mmol of **1a** was used.

f 0.12 mmol of **2a** was used.

g Reaction was performed under a N_2_ atmosphere.

h Reaction was performed on a 0.2 mmol scale.

With the optimized reaction conditions in hand (entry 16, Table 1), we evaluated the scope of the vinylogous Mannich reaction with an assortment of isatin-derived ketimines **1** with several substituents in different positions (Scheme 2). Initially, substitution with different groups such as methyl, benzyl, or allyl at the N-1 of the oxindole was evaluated (**3aa–3fa**), providing the corresponding products in good yields (52–68%) with high enantioselectivities (97–98% ee). We also tested isatin-derived ketimines with Ph, −CH_2_OMe, and H substituents at the N-1 and obtained excellent enantioselectivities (92–96% ee) but lower yields (30–42%). Because the best yield was obtained with *N*-benzyl isatin-derived ketimines, we evaluated the effect of the substitution pattern of several *N*-benzylisatines. Electron-withdrawing (Br or Cl) or electron-donating (MeO) groups were tolerated at the five-position of the isatin-derived ketimine, affording the corresponding products **3ha** and **3ia** in good yields with excellent enantioselectivities in all cases. However, with the ketimine prepared from 5-bromoisatin, the yield was low (28%). Furthermore, isatin-derived ketimines with substituents at the six- or seven-positions reacted efficiently, providing the Mannich products **3ja** and **3ka** in good yields (67–75%) with excellent stereoselectivities. Also, the disubstituted ketimine **1l** could be used, affording the corresponding product **3la** with excellent enantioselectivity (98% ee) in 66% yield. The reaction could be accomplished on a 1 mmol scale, improving the yield of product **3ba** (83%) and maintaining the enantioselectivity of the reaction (96% ee). We also tested the reaction on a 1 mmol scale lowering the catalyst amount to 2 mol % of **IV**. In this case, we observed a similar enantioselectivity (95% ee) but a lower yield (54%).

**Scheme 2:**
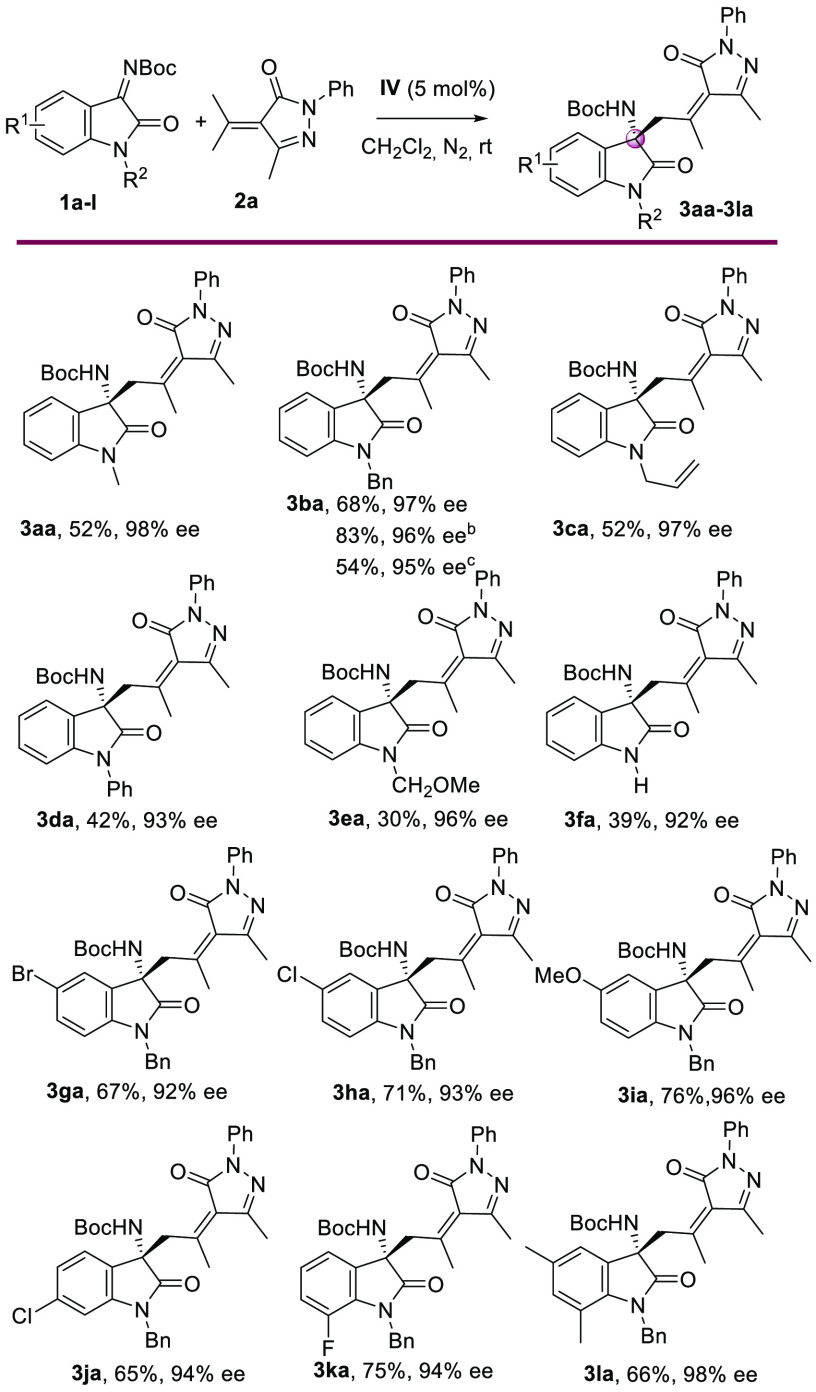
Scope of the Catalytic Enantioselective Vinylogous Addition of Alkylidenepyrazolone **2a** to Isatin-Derived Ketimines **1** Reaction conditions: **1** (0.2 mmol), **2** (0.2 mmol), and **IV** (5 mol %) in 1 mL of CH_2_Cl_2_ at rt under a N_2_ atmosphere. Isolated yield of **3** after column chromatography. Determined by chiral HPLC. 1 mmol scale reaction using 5 mol % of **IV**. 1 mmol scale reaction using 2 mol % of **IV**.

Next, we turned our attention to further explore the substrate scope with respect to the alkylidenepyrazolones **2** (Scheme 3). Alkyl groups (Et, Pr, and cyclopropyl) other than Me were well tolerated at the five-position of the pyrazolones (**3bb**–**3bd**), providing excellent yields (84–98%) and enantioselectivities (95–96% ee). 2,5-Diphenyl and 2,5-dimethyl alkylidenepyrazolone were also examined under the optimized reaction conditions, providing products **3ae** in 68% yield with 94% ee and **3bf** in 73% yield with 99% ee. Moreover, when alkylidenepyrazolone derived from acetophenone was tested with ketimines **1a** and **1b**, the corresponding Mannich products **3ag** and **3bg** were obtained with excellent results. Finally, the reaction proceeded efficiently with pyrazolones with different substituents (NO_2_, Cl, Me, and MeO) on the *N*-aryl group, providing the corresponding products in high yields (63–90%) with high enantiomeric excesses (93–97% ee).

**Scheme 3:**
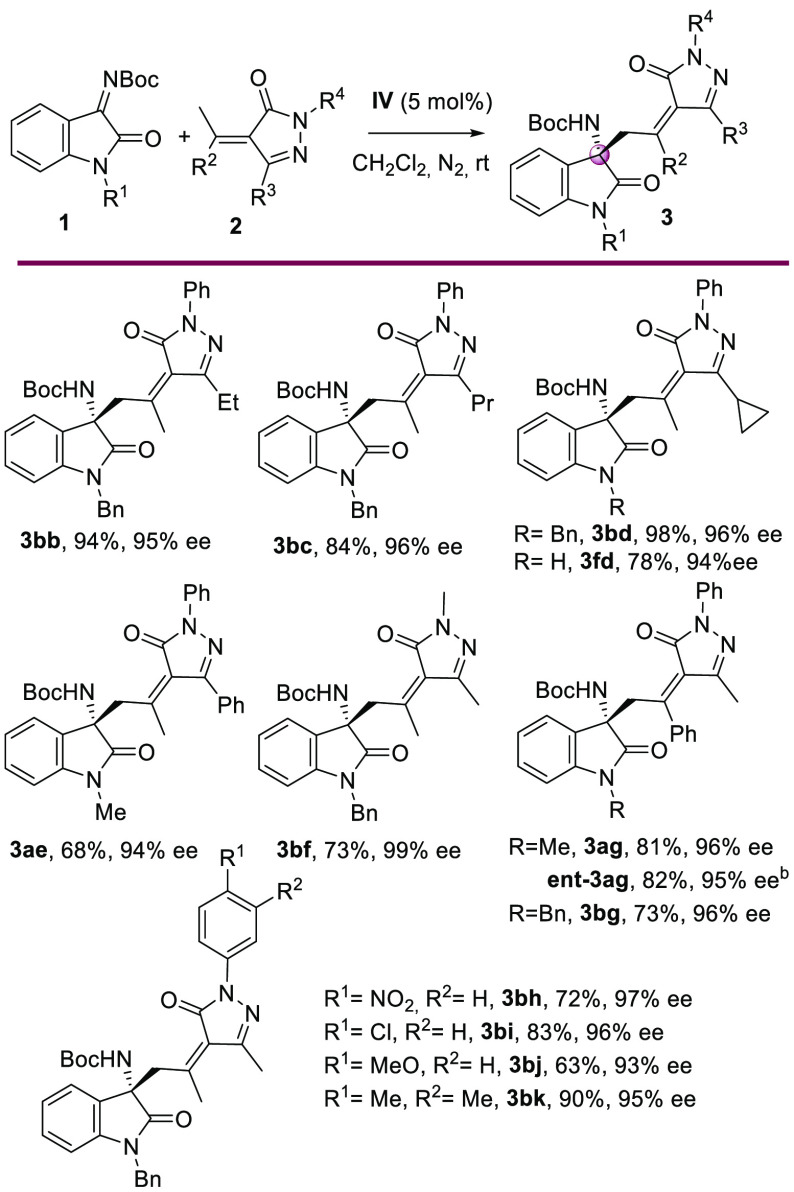
Scope of the Catalytic Enantioselective Vinylogous Addition of Alkylidenepyrazolone **2** to Isatin-Derived Ketimines **1** Reaction conditions: **1** (0.2 mmol), **2** (0.2 mmol), and **IV** (5 mol %) in 1 mL of CH_2_Cl_2_ at rt under a N_2_ atmosphere. Isolated yield of **3** after column chromatography. Determined by chiral HPLC. **VIII** was used.

The double-bond configuration and absolute configuration of the stereogenic center present in compound **3ea** were ascertained to be (S, Z) by X-ray crystallographic analysis (Scheme 4); the configuration of the remaining Mannich products **3** was assigned on the assumption of a uniform mechanistic reaction pathway. A plausible transition-state model is illustrated in Scheme 4, where the squaramide catalyst **IV** is responsible for the preorientation and the activation of the substrates of the reaction. Whereas the methyl group of alkylidenepyrazolones is first deprotonated by the quinuclidine moiety of the organocatalyst to form the corresponding diene enolate, the isatin-derived *N*-Boc-ketimine moiety is activated upon the formation of hydrogen bonds between the *N*-Boc group and the squaramide moiety of the organocatalyst. The pyrazolone enolate will be directed to the *Re*-face of the ketimine, thus accounting for the observed enantioselectivity.

**Scheme 4:**
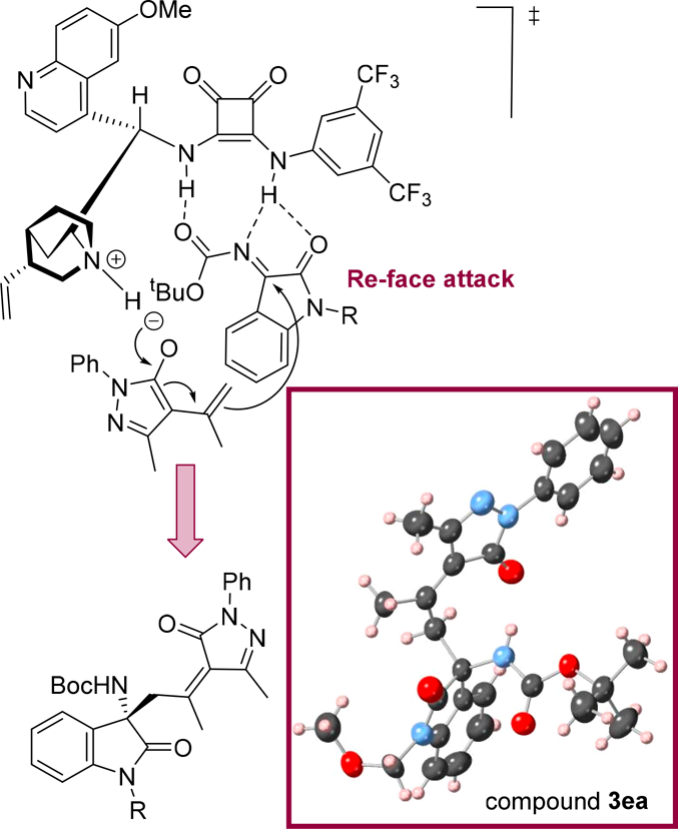
Proposed Reaction Mechanism for the Asymmetric Vinylogous Mannich Reaction and X-ray Crystal Structure of **3ea**

To demonstrate the versatility and usefulness of our organocatalytic vinylogous methodology, we performed several chemical modifications of the Mannich products (Scheme 5). A relevant structural feature of the obtained vinylogous Mannich adducts is that they preserve the exocyclic unsaturation of the initial pyrazolone substrate. This olefinic group could be used to further functionalize the compound, thus increasing the molecular complexity of the pyrazolone products. For example, compound **3ba** was stereoselectively epoxidated with *meta*-chloroperoxybenzoic acid (mCPBA), affording the spirooxirane **4** (Scheme 5A) with three quaternary stereocenters in 90% yield with good diastereoselectivity (84:16 dr) and maintaining the enantiomeric excess.^12^ We could obtain crystals of the major diastereoisomer **4**, which allowed us to determine the configuration of the epoxide. Moreover, compound **3ba** could be subjected to a conjugate addition of NaCN,^14^ providing the corresponding product **5** as a single diastereoisomer in good yield (73%) and maintaining the enantiomeric excess (Scheme 5B). Finally, the reaction of compound **3ba** with methyl bromoacetate in the presence of NaH^15^ afforded the highly functionalized chiral pyrazole **6** in 89% yield and preserved the optical purity of the starting material (Scheme 5C).

**Scheme 5:**
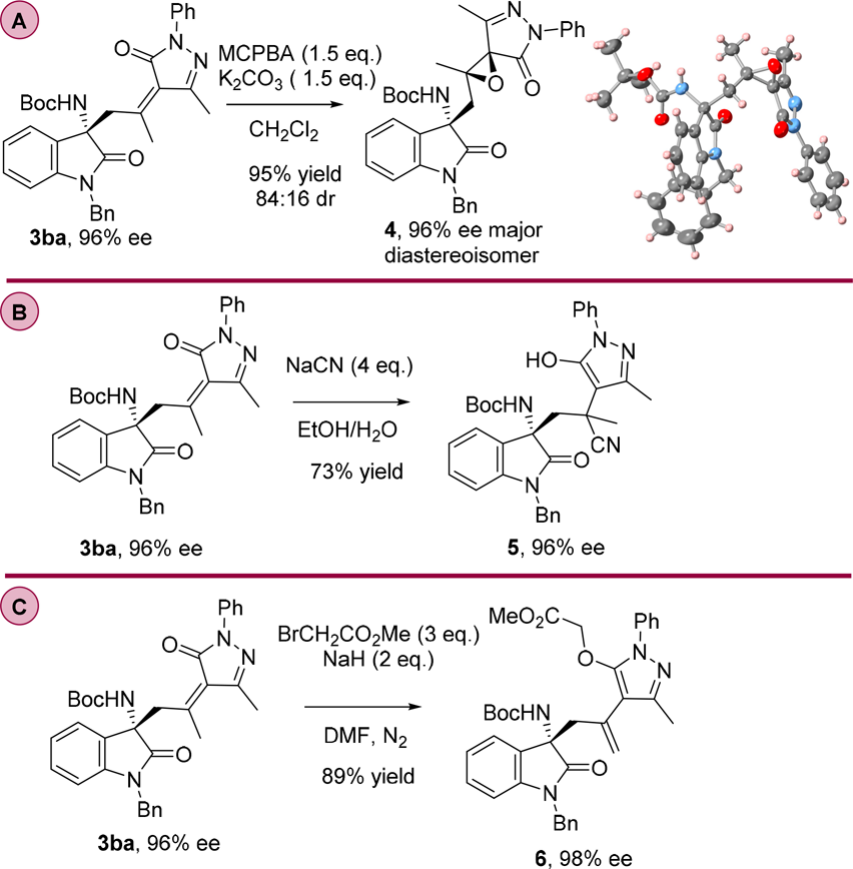
Synthetic Transformations

In summary, we have established an organocatalytic diastereo- and enantioselective vinylogous Mannich reaction of alkylidenepyrazolones with isatin-derived ketimines using a quinine-derived squaramide organocatalyst, obtaining the corresponding chiral Mannich adducts in moderate to high yields (up to 98%) with complete diastereoselectivities toward the Z double bond and excellent enantioselectivities (up to 99% ee) under mild reaction conditions. The reaction showed a wide substrate scope for different *N*-Boc-ketimines and alkylidenepyrazolones. The new compounds feature pyrazolone and amino-oxindole moieties, which are privileged structures in medicinal chemistry. Moreover, several synthetic transformations of the chiral Mannich product **3ba** have been performed, showing the potential applicability of the present methodology. Studies on extending the scope of the reaction are currently ongoing in our laboratory.

## Supporting Information Available

Complete experimental procedures and characterization of new products and ^1^H and ^13^C NMR spectra for all compounds (PDF)

## Accession Codes

CCDC 2092891–2092892 contain the supplementary crystallographic data for this paper. These data can be obtained free of charge via www.ccdc.cam.ac.uk/data_request/cif, or by emailing data_request@ccdc.cam.ac.uk, or by contacting The Cambridge Crystallographic Data Centre, 12 Union Road, Cambridge CB2 1EZ, UK; fax: +44 1223 336033.
