# Supplementary material for: Catalytic Diastereo- and Enantioselective Vinylogous Mannich Reaction of Alkylidenepyrazolones to Isatin-Derived Ketimines
Source: Org Lett. 2021 Sep 23;23(19):7391–5. doi: 10.1021/acs.orglett.1c02571 (PMC8491163; doi:10.1021/acs.orglett.1c02571)
Supplement: Supplementary file 1 — ol1c02571_si_001.pdf [file ol1c02571_si_001.pdf]

Catalytic Diastereo- and Enantioselective Vinylogous Mannich Reaction of Alkylidenepyrazolones to Isatin-Derived Ketimines.

Laura Carceller-Ferrer,[a] Carlos Vila,[a] Gonzalo Blay,[a*] M. Carmen Muñoz,[b] José R. Pedro[a*]

[a] Departament de Química Orgànica, Facultat de Química, Universitat de València, Dr. Moliner 50, 46100 Burjassot, València (Spain). Email: gonzalo.blay@uv.es; jose.r.pedro@uv.es

[b] Departament de Física Aplicada, Universitat Politècnica de València, Camino de Vera s/n, 46022 València (Spain)

Contents

General Experimental Methods ……………………………………………………..……. S1

Experimental procedures……………………………………………………………..…..S2

i. Synthesis of alkylidene pyrazolones **2b**, **2d**, **2f** and **2k**……………………..….S2
ii. General procedure for the racemic reaction between isatin-derived imines **1** and alkylidene pyrazolones…………………………………………………………..…S2
iii. Procedure for the enantioselective reaction between isatin-derived imines **1** and alkylidene pyrazolones **2** ……………………………………………………..…S2
iv. Procedure for the enantioselective reaction between isatin-derived imines 1a and alkylidene pyrazolones 2g using squaramide VIII....................................S3
v. Procedure for the enantioselective 1mmol reaction between isatin-derived imines 1b and alkylidene pyrazolones 2a…………………………………………………S3
vi. Synthetic transformation I: epoxidation of the double bond……………………..S3
vii. Synthetic transformation II: Michael addition of cyanide ………………………..S4
viii. Synthetic transformation III: pyrazole formation ………………………………..S4

Characterization of the starting materials ………………………………………………..S5

Characterization of the final amines **3**……………………………………………………S6

Characterization of product **4**……………………………………………………………...S16

Characterization of product **5**…………………………………………………………….. S17

Characterization of product **6** ……………………………………………………………. S18

NMR data (^{1}H-NMR and ^{13}C-NMR)………………………………………………………S19

HPLC data………………………………………………………………………………...S52

GENERAL EXPERIMENTAL METHODS

Commercial reagents were used without any further purification. Reactions progress was monitored by TLC (Thin Layer Chromatography) analysis using Merck Silica Gel 60 F-254 thin layer plates. Flash column chromatography was used for the purifications of the products using Merck silica gel 60, 0.040–0.063 mm. NMR spectra were run in a Bruker DPX300 spectrometer (Bruker, Billerica, MA, USA) at 300 MHz for ^{1}H and at 75 MHz for ^{13}C using residual non-deuterated solvent as internal standard ($CHCl_3$: δ 7.26 for 1 H and 77.0 ppm for ^{13}C; DMSO-d_6 δ 2.50 for ^{1}H and 39.52 ppm for ^{13}C). Chemical shifts are given in ppm. The carbon type was determined by DEPT (Distortionless Enhancement by Polarization Transfer) experiments. High resolution mass spectra (ESI) were recorded on a TRIPLETOFT5600 spectrometer with a QTOF mass analyzer (AB Sciex, Warrington, UK) equipped with an electrospray source with a capillary voltage of 4.5 kV (ESI). Specific optical rotations were measured using sodium light (D line 589 nm). Chiral HPLC (High Performance Liquid Chromatography) analyses were carried out in a chromatograph equipped with a UV diode-array detector using chiral stationary columns from Daicel and Phenomenex. Isatin-derived imines **1** were prepared as described in the literature.[1] Some alkylidene pyrazolones derivatives **2** were prepared as described in literature and others (**2b**, **2d**, **2f** and **2k**) were synthetized modifying the existing references.[2]

References

1. Yan, W., Wang, D., Feng, J., Li, P., Zhao, D., Wang, R. *Org. Lett*. **2012**, *14*, 2512-2515.
2. (a) Deruiter, J., Carter, D. A., Arledge, W. S., Sullivan, P. J.J. *Heterocycl. Chem.* 1987, *24*, 149-153 (**2a**, **2h**, **2i** and **2j**); (b) Rassu, G., Zambrano, V., Pinna, L., Curti, C., Battistini, L., Sartori, A., Pelosi, G., Casiraghi, G., Zanardi, F. *Adv. Synth. Catal.* **2014**, *356*, 2330 – 2336 (**2c** and **2e**); (c) Zheng, L., Li, P., Gu, M., Lin, A., Yao, H. *Org. Lett*. **2017**, *19*, 2829−2832 (**3g**).

EXPERIMENTAL PROCEDURES

i. Procedure for the synthesis of alkylidene pyrazolones 2b, 2d, 2f and 2k[2a]

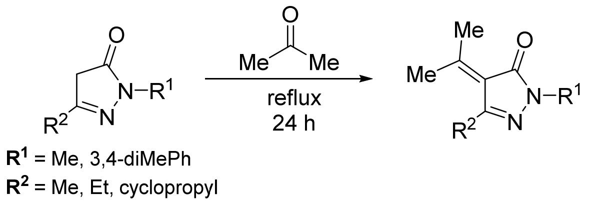

Alkyliden pyrazolones **2b**, **2d**, **2f** and **2k** were synthetized according to reference 2a. The corresponding pyrazolone (5 mmol) was dissolved in acetone (5 mL) and heated to reflux for 24 h. Then, the reaction solution was cooled down to 0 ºC and water (2-5 mL) was added until of a yellow solid precipitated. Filtration of the crude reaction and several washes with cold water afforded alkylidene pyrazolones **2** in good yields (56-64%).

ii. General procedure for the racemic reaction between isatin-derived imines 1 and alkylidene pyrazolones 2.

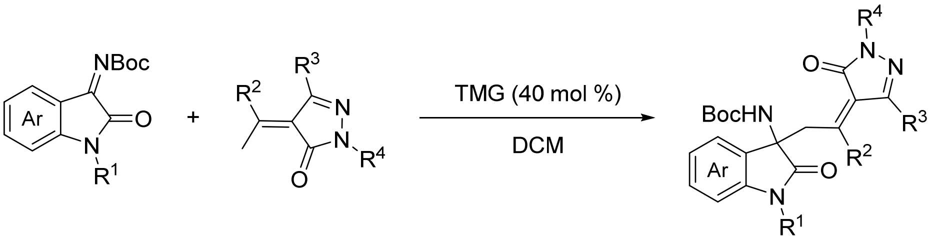

In a 5 mL vial, the corresponding isatin-derived imine (**1**, 0.1 mmol) and alkylidene pyrazolone (**2**, 0.1 mmol) were dissolved in DCM (1 mL). Then, trymethylguanidine (TMG, 40 mol %, 0.04 mmol, 5µL) was added and the mixture was stirred at room temperature until completion (TLC). The resulting product was purified by column chromatography being eluted with DCM/Et_2O 98:2 to 90:10.

iii. Procedure for the enantioselective reaction between isatin-derived imines 1 and alkylidene pyrazolones 2.

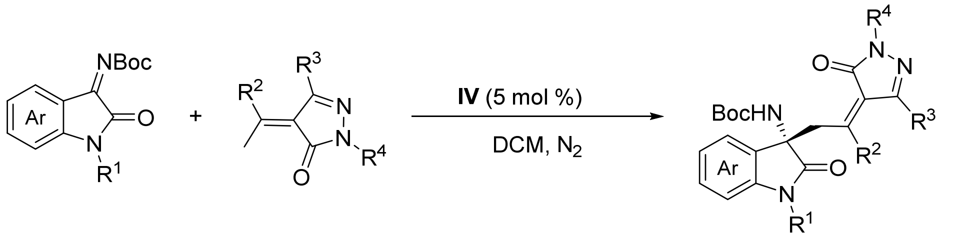

In a 5 mL vial, the corresponding corresponding isatin-derived imine (**1**, 0.1 mmol) and alkylidene pyrazolone (**2**, 0.1 mmol) and catalyst (**IV**, 5 mol %, 0.005 mmol, 3.2 mg) were dissolved in DCM (1 mL) and the reaction mixture was left at room temperature for 3 days. The crude product was purified by column chromatography being eluted with DCM/Et_2O 98:2 to DCM/Et_2O 90:10, affording the corresponding amines as yellow solids.

iv. Procedure for the enantioselective reaction between isatin-derived imines 1a and alkylidene pyrazolones 2g using squaramide VIII.

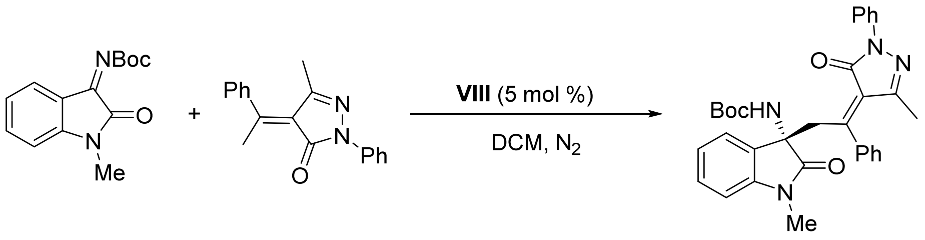

In a 5 mL vial, isatin-derived imine **1a** (26.0 mg, 0.1 mmol) and alkylidene pyrazolone **2g** (27.6 mg, 0.1 mmol) and catalyst (**VIII**, 5 mol %, 0.005 mmol, 3.2 mg) were dissolved in DCM (1 mL) and the reaction mixture was left at room temperature for 3 days. The crude product was purified by column chromatography being eluted with DCM/Et_2O 98:2 to DCM/Et_2O 90:10, affording the corresponding product ent-**3ag** as a yellow solid (43.9 mg, 0.082 mmol, 82% yield, 95% ee).

v. Procedure for the enantioselective 1mmol reaction between isatin-derived imines 1b and alkylidene pyrazolones 2a.

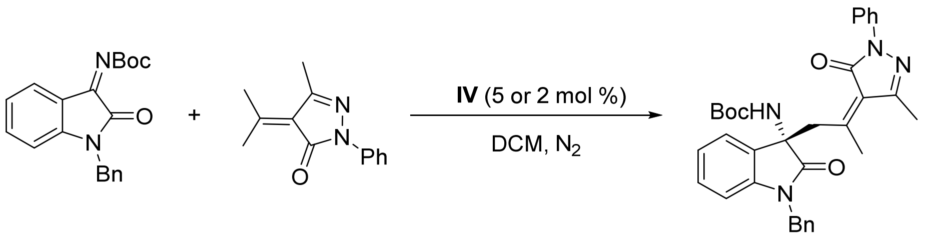

In a 25 mL round-bottom flask, isatin-derived imine **1b** (336.4 mg**,** 1 mmol) and alkylidene pyrazolone **2a** (214.0 mg, 1 mmol) and catalyst **IV** (5 mol %, 0.05 mmol, 32.0 mg or 2 mol%, 0.02 mmol, 12.8 mg) were dissolved in DCM (10 mL) and the reaction mixture was left at room temperature for 4 days. The crude product was purified by column chromatography being eluted with DCM/Et_2O 98:2 to DCM/Et_2O 90:10, affording the corresponding product **3ba** as a yellow solid (456.9 mg, 0.83 mmol, 83% yield, 96% ee using 5 mol% of **IV** and 297.5 mg, 0.54 mmol, 54% yield, 95% ee using 2 mol% of **IV**).

vi. Synthetic transformation I: epoxidation of the double bond

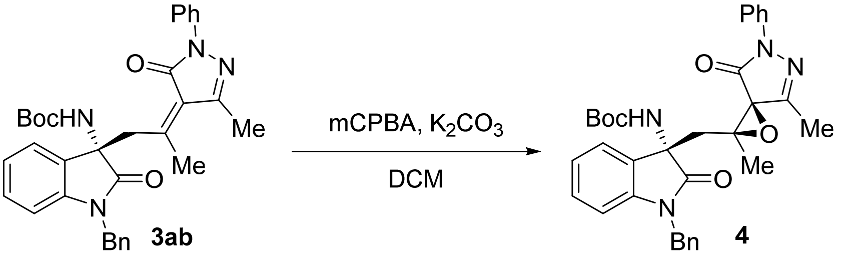

mCPBA (1.5 eq., 0.11 mmol, 19.0 mg) was added dropwise at 0 ºC to a solution of the product **3ba** (0.073 mmol, 40.2 mg) in DCM (1 mL). Then, K_2CO_3 (1.5 eq., 0.11 mmol, 15.2 mg) was added maintaining the solution in the ice-bath and the reaction mixture was stirred at 0 ºC for 1h. After the reaction is completed, the mixture is diluted with H_2O (10 mL) and extracted with DCM (3x20 mL). The combined organic layers were dried over $MgSO_4$ (anh.) and solvent was removed under reduced pressure. Product **4** was obtained (39.3 mg, 0.069 mmol, 95% yield) as a mixture of diastereoisomers (*dr* 84:16)

that can be separated by column chromatography being eluted with hexane/Et_2O 70:30 to hexane/Et_2O 40:60.

vii. Synthetic transformation II: Michael addition of cyanide

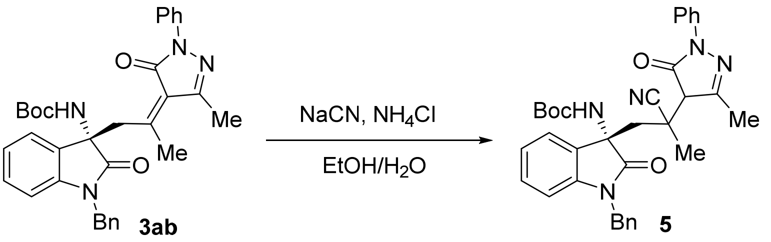

To a solution of product **3ba** (0.073 mmol, 40.2 mg) in EtOH (1 mL), a solution of KCN (4 eq., 0.286 mmol, 18.6 mg) and NH_4Cl (1.75 eq., 0.128 mmol, 7 mg) in water (1 mL) was added at room temperature. The reaction was then heated to reflux until reaction completed (TLC). After 90 min., H_2O (10 mL) was added to the crude mixture at room temperature and extracted with AcOEt (20 mLx3). The organic layers were combined and dried over $MgSO_4$ (anh.). The residue was purified by flash column chromatography being eluted with DCM/Et_2O 95:5 to 90:10 to obtain the pure product as a white solid (30.8 mg, 73% yield).

viii. Synthetic transformation III: pyrazole formation

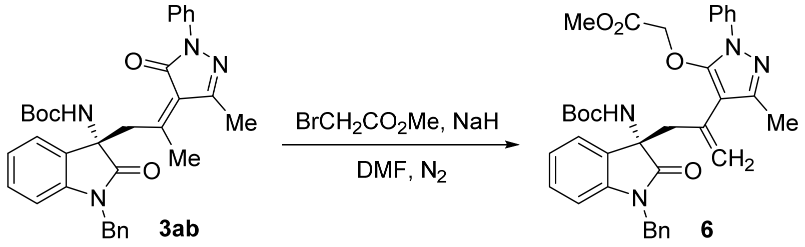

Product **3ba** (0.073 mmol, 40.2 mg) was weight in a round-bottom flask and purged with N_2. DMF (5 mL) was added. NaH (60% mineral oil) (2 eq., 0.145 mmol, 6.0 mg) was added in portions to the solution at 0 ºC and it was left stirring until no H_2 is observed. Still in the ice-bath, methyl bromoacetate (3 eq., 0.218 mmol, 20,6 µL) was added and the reaction mixture was heated to 80 ºC for 1 h. After the reaction is completed, cold water was added (10 mL) followed by the extraction with AcOEt (20 mLx3). The organic layers were combined and dried over $MgSO_4$ (anh.) and solvent was removed using the rotavapor. The residue was purified by flash-column chromatography using hexane/Et_2O 60:40 to hexane/Et_2O 50:50 as eluent, affording the desired product as white solid (40.6 mg, 89% yield).

CHARACTERITZATION OF THE STARTING MATERIALS: ALKYLIDENE PYRAZOLONES 2 DERIVATES

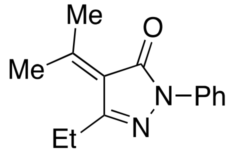

2b

Product **2b** has been synthesized following procedure i as a yellow solid (650 mg, 57% yield).
^{1}H NMR (300 MHz, CDCl$_3$) δ 7.89 (d, *J* = 7.7 Hz, 2H), 7.32 (t, *J* = 7.9 Hz, 2H), 7.10 (d, *J* = 7.3 Hz, 1H), 2.70 (q, *J* = 7.3 Hz, 2H), 2.55 (s, 3H), 2.29 (s, 3H), 1.26 (t, *J* = 7.3 Hz, 3H). **^{13}C NMR (75 MHz, CDCl$_3$)** δ 165.1 (C), 163.9 (C), 152.2 (C), 138.5 (C), 128.7 (CH), 125.0 (C), 124.5 (CH), 118.9 (CH), 25.6 (CH$_2$), 25.1 (CH$_3$), 23.1 (CH$_3$), 11.1 (CH$_3$). **HRMS (ESI/Q-TOF)** m/z: [M+H]$^+$ $C_{14}H_{17}N_2O^+$ Calcd for 229.1335; Found 229.1327.

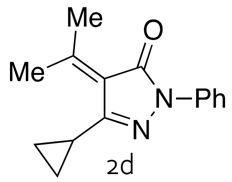

2d

Product **2d** has been synthesized following procedure i as a yellow solid (709 mg, 59% yield).
^{1}H NMR (300 MHz, CDCl$_3$) δ 7.93 (dd, *J* = 8.7, 1.0 Hz, 2H), 7.37 (t, *J* = 8.5 Hz, 2H), 7.14 (t, *J* = 7.4 Hz, 1H), 2.64 (s, 3H), 2.55 (s, 3H), 1.97 – 1.76 (m, 1H), 1.16 – 1.00 (m, 2H), 1.00 – 0.83 (m, 2H)**. ^{13}C NMR (75 MHz, CDCl$_3$)** δ 166.2 (C), 163.8 (C), 151.9 (C), 138.5 (C), 128.6 (CH), 125.4 (C), 124.5 (CH), 118.9 (CH), 25.1 (CH$_3$), 23.0 (CH$_3$), 12.6 (CH), 6.4 (CH$_2$). **HRMS (ESI/Q-TOF)** m/z: [M+H]$^+$ $C_{15}H_{17}N_2O^+$ Calcd for 241.1335; Found 241.1343.

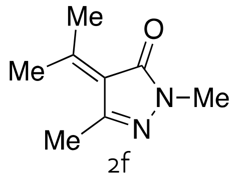

2f

Product **2f** has been synthesized following procedure i as a yellow solid (488 mg, 64% yield).
^{1}H NMR (300 MHz, CDCl$_3$) δ 3.26 (s, 3H), 2.51 (s, 3H), 2.25 (s, 6H). ^{13}C NMR (75 MHz, CDCl$_3$) δ 165.0 (C), 164.8 (C), 146.4 (C), 124.7 (C), 30.7 (CH$_3$), 24.5(CH$_3$), 22.5(CH$_3$), 18.7 (CH$_3$). **^{13}C NMR (75 MHz, CDCl$_3$)** δ 165.0 (C), 164.8 (C), 146.4 (C), 124.7 (C), 30.7 (CH$_3$), 24.5 (CH$_3$), 22.5 (CH$_3$), 18.7 (CH$_3$). **HRMS (ESI/Q-TOF)** m/z: [M+H]$^+$ $C_8H_{13}N_2O^+$ Calcd for 153.1022; Found 153.1027.

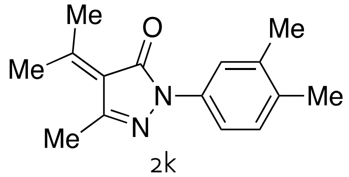

2k

Product **2k** has been synthesized following procedure i as a yellow solid (679 mg, 56% yield).
^{1}H NMR (300 MHz, CDCl$_3$) δ 7.63 (d, *J* = 2.0 Hz, 1H), 7.55 (dd, *J* = 8.2, 2.3 Hz, 1H), 7.07 (d, *J* = 8.2 Hz, 1H), 2.55 (s, 3H), 2.35 (s, 3H), 2.29 (s, 3H), 2.22 (s, 3H), 2.18 (s, 3H). **^{13}C NMR (75 MHz, CDCl$_3$)** δ 165.6 (C), 163.5 (C), 147.7 (C), 136.9 (C), 136.2 (C), 133.0 (C), 129.7 (CH), 125.5 (C), 120.2 (CH), 116.6 (CH), 24.9 (CH$_3$), 22.9 (CH$_3$), 20.0 (CH$_3$), 19.2 (CH$_3$), 19.1 (CH$_3$). **HRMS (ESI/Q-TOF)** m/z: [M+H]$^+$ $C_{15}H_{19}N_2O^+$ Calcd for 243.1492; Found 243.1500.

CHARACTERIZATION OF THE FINAL AMINES 3

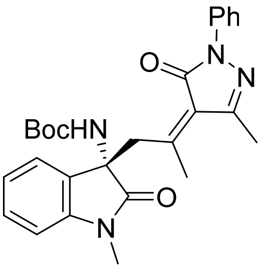

3aa

Following the General Procedure **iii**, product **3aa** was obtained as a yellow solid (24.7 mg, 52% yield) using as eluent mixtures of DCM/Et_2O 98:2 to DCM/Et_2O 90:10. Enantiomeric excess (98%) was determined by chiral HPLC (Chiralpak® ADH, *i*PrOH/hexane = 20/80, flow rate= 1.0 mL/min, λ = 254 nm) t_R = 19.15 min (major), 16.11 min (minor). $[\alpha_D^{20}]$= -211.4 (c 0.5, $CHCl_3$). m.p.= 184-185 ºC.

^{1}H NMR (300 MHz, $CDCl_3$) δ 7.91 (dd, *J* = 8.8, 1.1 Hz, 2H), 7.45-7.35 (m, 4H), 7.29 (td, *J* = 7.7, 1.2 Hz, 1H), 7.20 (t, *J* = 7.4 Hz, 1H), 7.05 (td, *J* = 7.7, 0.9 Hz, 1H), 6.85 (d, *J* = 7.7 Hz, 1H), 3.99 (d, *J* = 12.5 Hz, 1H), 3.27 (s, 3H), 2.91 (d, *J* = 11.2 Hz, 1H), 2.42 (s, 3H), 2.22 (s, 3H), 1.23 (s, 9H). **^{13}C NMR (75 MHz, $CDCl_3$)** δ 175.9 (C), 175.9 (C), 164.1 (C), 163.0 (C), 154.1 (C), 149.0 (C), 142.6 (C), 137.8 (C), 131.6 (C), 129.2 (C), 129.0 (CH), 128.8 (CH), 125.3 (CH), 122.8 (CH), 122.5 (CH), 119.5 (CH), 108.1 (CH), 79.9 (C), 63.8 (C), 42.2 (CH_2), 28.0 (CH_3), 26.6 (CH_3), 25.9 (CH_3), 19.2 (CH_3). **HRMS (ESI/Q-TOF)** m/z: $[M+H]^+$ $C_{27}H_{31}N_4O_4^+$ Calcd for 475.2340; Found 475.2335.

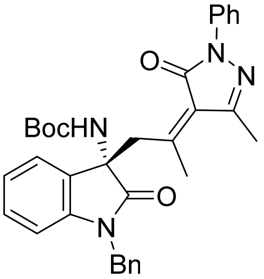

3ba

Following the General Procedure **iii**, product **3ba** was obtained as a yellow solid (37,4 mg, 68% yield) using as eluent mixtures of DCM/Et_2O 98:2 to DCM/Et_2O 90:10. Enantiomeric excess (97%) was determined by chiral HPLC (Chiralpak® ADH *i*PrOH/hexane = 20/80, flow rate= 1.0 mL/min, λ = 254 nm), t_R = 26.87 min (major), 19.43 min (minor). $[\alpha_D^{20}]$= -118.7 (c 0.8, $CHCl_3$). m.p.= 177-178 ºC.

^{1}H NMR (300 MHz, $CDCl_3$) δ 7.92 (dd, *J* = 8.8, 1.1 Hz, 2H), 7.49 (s, 1H, NH), 7.46-7.27 (m, 8H), 7.25 – 7.15 (m, 2H), 7.03 (t, *J* = 8.0 Hz, 1H), 6.77 (d, *J* = 7.9 Hz, 1H), 5.19 (s, 1H), 4.79 (s, 1H), 4.11 (d, *J* = 12.3 Hz, 1H), 2.42 (s, 3H), 2.18 (s, 3H), 1.26 (s, 9H). **^{13}C NMR (75 MHz, $CDCl_3$)** δ 175.9 (C), 164.0 (C), 162.9 (C), 154.2 (C), 149.0 (C), 141.8 (C), 137.8 (C), 136.0 (C), 131.6 (C), 129.2 (C), 128.9 (CH), 128.8 (CH), 128.7 (CH), 127.6 (CH), 127.4 (CH), 125.3 (CH), 122.7 (CH), 122.5 (CH), 119.5 (CH), 109.1 (CH), 79.9 (C), 63.9 (C), 44.1 (CH_2), 42.5 (CH_2), 28.0 (CH_3), 26.0 (CH_3), 19.2 (CH_3). **HRMS (ESI/Q-TOF)** m/z: $[M+H]^+$ $C_{33}H_{35}N_4O_4^+$ Calcd for 551.2653; Found 551.2651.

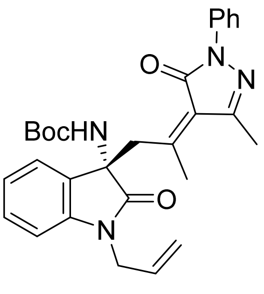

3ca

Following the General Procedure **iii**, product **3ca** was obtained as a yellow solid (26.0 mg, 52% yield) using as eluent mixtures of DCM/Et_2O 98:2 to DCM/Et_2O 90:10. Enantiomeric excess (97%) was determined by chiral HPLC (Chiralpak® ADH *i*PrOH/hexane = 20/80, flow rate= 1.0 mL/min, λ = 254 nm), t_R = 15.31 min (major), 9.29 min (minor). [α_D^{20}]= -197.8 (c 0.6, $CHCl_3$). m.p.= 179-180 ºC.

^{1}H NMR (300 MHz, $CDCl_3$) δ 7.91 (dd, *J* = 8.8, 1.2 Hz, 2H), 7.47 (s, 1H, NH), 7.45-7.34 (m, 3H), 7.30-7.15 (m, 2H), 7.04 (td, *J* = 7.6, 0.8 Hz, 1H), 6.85 (d, *J* = 7.7 Hz, 1H), 6.00-5.83 (m, 1H), 5.34 (dd, *J* = 17.2, 1.3 Hz, 1H), 5.25 (dd, *J* = 10.3, 1.3 Hz, 1H), 4.61 (s, 1H), 4.18 (s, 1H), 4.09 (d, *J* = 12.4 Hz, 1H), 2.84 (d, *J* = 11.9 Hz, 1H), 2.42 (s, 3H), 2.22 (s, 3H), 1.24 (s, 9H). **^{13}C NMR (75 MHz, $CDCl_3$)** δ 175.6 (C), 164.0 (C), 163.0 (C), 154.1 (C), 149.0 (C), 141.7 (C), 137.8 (C), 131.7 (CH), 129.2 (C), 128.83 (CH), 128.77 (CH), 128.68 (C), 125.3 (CH), 122.7 (CH), 122.5 (CH), 119.6 (C), 118.8 (C), 117.5 (CH2), 109.0 (CH), 79.9 (C), 63.9 (C), 42.6 (CH_2), 42.5 (CH_2), 28.0 (CH_3), 26.0 (CH_3), 19.2 (CH_3). **HRMS (ESI/Q-TOF)** m/z: $[M+H]^+$ $C_{29}H_{33}N_4O_4^+$ Calcd for 501.2496; Found 501.2487.

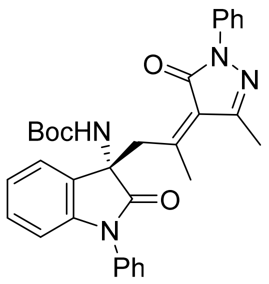

3da

Following the General Procedure **iii**, product **3da** was obtained as a yellow solid (22.7mg, 42% yield) using as eluent mixtures of DCM/Et_2O 98:2 to DCM/Et_2O 90:10. Enantiomeric excess (93%) was determined by chiral HPLC (Chiralpak® ADH *i*PrOH/hexane = 20/80, flow rate= 1.0 mL/min, λ = 254 nm), t_R = 14.76 min (major), 11.85 min (minor). [α_D^{20}]= -115.0 (c 0.5, $CHCl_3$). m.p.= 194-195 ºC.

^{1}H NMR (300 MHz, $CDCl_3$) δ 7.92 (dd, *J* = 8.7, 1.2 Hz, 2H), 7.55-7.37 (m, 9H), 7.25-7.17 (m, 2H), 7.10 (dd, *J* = 7.4, 0.8 Hz, 1H), 6.85 (d, *J* = 7.3 Hz, 1H), 4.12 (d, *J* = 12.5 Hz, 1H), 3.13 (d, *J* = 12.5 Hz, 1H), 2.41 (s, 3H), 2.28 (s, 3H), 1.26 (s, 9H). **^{13}C NMR (75 MHz, $CDCl_3$)** δ 175.2 (C), 164.0 (C), 162.9 (C), 154.3 (C), 148.9 (C), 142.6 (C), 137.8 (C), 134.7 (C), 131.4 (C), 129.6 (CH), 129.2 (C), 128.82 (CH), 128.80 (CH), 128.0 (CH), 126.6 (CH), 125.3 (CH), 123.2 (CH), 122.9 (CH), 119.6 (CH), 109.4 (CH), 80.0 (C), 63.9 (C), 42.3 (CH_2), 28.2 (CH_3), 26.0 (CH_3), 19.2 (CH_3). **HRMS (ESI/Q-TOF)** m/z: $[M+H]^+$ $C_{32}H_{33}N_4O_4^+$ Calcd for 537.2496; Found 537.2501.

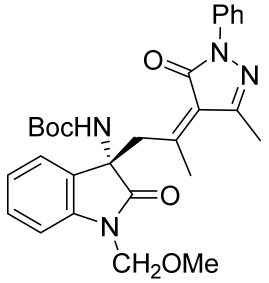

3ea

Following the General Procedure **iii**, product **3ea** was obtained as a yellow solid (17.2 mg, 30% yield) using as eluent mixtures of DCM/Et_2O 98:2 to DCM/Et_2O 90:10. Enantiomeric excess (96%) was determined by chiral HPLC (Chiralpak® ADH *i*PrOH/hexane = 20/80, flow rate= 1.0 mL/min, λ = 254 nm), t_R =14.67 min (major), 8.65 min (minor). [α_D^{20}]= +135.7 (c 0.6, $CHCl_3$). m.p.= 189-190 ºC.

^{1}H NMR (300 MHz, $CDCl_3$) δ 7.92 (dd, *J* = 8.8, 1.2 Hz, 2H), 7.52 (s, 1H), 7.46-7.36 (m, 3H), 7.30 (td, *J* = 7.7, 1.3 Hz, 1H), 7.25-7.17 (m, 1H), 7.12-7.02 (m, 2H), 5.28 (d, *J* = 10.8 Hz, 1H), 5.12 (s,

1H), 4.01 (d, J = 12.5 Hz, 1H), 3.44 (s, 3H), 2.93 (d, J = 12.5 Hz, 1H), 2.43 (s, 3H), 2.23 (s, 3H), 1.24 (s, 9H). **^{13}C NMR (75 MHz, $CDCl_3$)** δ 176.5 (C), 164.0 (C), 162.8 (C), 154.2 (C), 149.0 (C), 141.0 (C), 137.8 (C), 131.0 (C), 129.3 (C), 129.1 (CH), 128.8 (CH), 128.73 (C), 125.4 (CH), 123.3 (CH), 122.6 (CH), 119.6 (CH), 118.8 (C), 109.6 (C), 80.0 (C), 71.9 (CH2), 64.1 (C), 56.6 (CH_3), 42.5 (CH_2), 28.0 (CH_3), 26.1 (CH_3), 19.2 (CH_3). **HRMS (ESI/Q-TOF)** m/z: $[M+H]^+$ $C_{28}H_{33}N_4O_5^+$ Calcd for 505.2445; Found 505.2433.

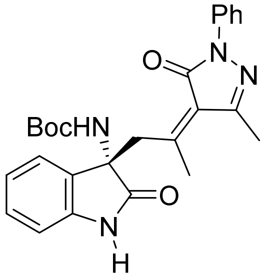

3fa

Following the General Procedure **iii**, product **3fa** was obtained as a yellow solid (18.0 mg, 39% yield) using as eluent mixtures of DCM/Et_2O 98:2 to DCM/Et_2O 90:10. Enantiomeric excess (92%) was determined by chiral HPLC (Chiralpak® ADH *i*PrOH/hexane = 20/80, flow rate= 1.0 mL/min, λ = 254 nm), t_R = 14.31 min (major), 10.69 min (minor). $[\alpha_D^{20}]$= -177.7 (c 0.5, $CHCl_3$). m.p.= 225-226 ºC.

^{1}H NMR (300 MHz, $CDCl_3$) δ 8.07 (s, 1H, NH), 7.91 (dd, J = 8.7, 1.2 Hz, 2H), 7.59 (s, 1H, NH), 7.42 (t, J = 7.9 Hz, 2H), 7.35 (d, J = 7.3 Hz, 1H), 7.23 (d, J = 7.6 Hz, 2H), 7.04 (dd, J = 7.5, 0.8 Hz, 1H), 6.85 (d, J = 7.7 Hz, 1H), 4.10 (d, J = 12.5 Hz, 1H), 2.86 (d, J = 12.5 Hz, 1H), 2.43 (s, 3H), 2.28 (s, 3H), 1.26 (s, 9H). 13C NMR (75 MHz, CDCl3) δ 177.8 (C), 164.1 (C), 163.4 (C), 154.5 (C), 149.1 (C), 139.9 (C), 137.8 (C), 132.1 (C), 129.3 (C), 129.0 (CH), 128.9 (CH), 128.7 (CH), 125.4 (CH), 122.8 (CH), 119.7 (CH), 110.3 (CH), 80.3 (C), 64.2 (C), 42.3 (CH_2), 28.1 (CH_3), 26.1 (CH_3), 19.3 (CH_3). **HRMS (ESI/Q-TOF)** m/z: $[M+H]^+$ $C_{26}H_{29}N_4O_4^+$ Calcd for 461.2183; Found 461.2174.

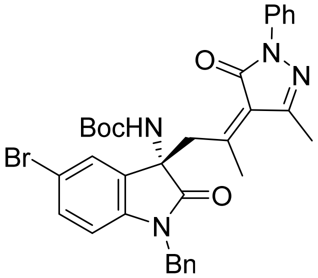

3ga

Following the General Procedure **iii**, product **3ga** was obtained as a yellow solid (42.3 mg, 67% yield) using as eluent mixtures of DCM/Et_2O 98:2 to DCM/Et_2O 90:10. Enantiomeric excess (92%) was determined by chiral HPLC (Chiralpak® ADH *i*PrOH/hexane = 20/80, flow rate= 1.0 mL/min, λ = 254 nm), t_R = 19.63 min (major), 15.37 min (minor). $[\alpha_D^{20}]$= -46.4 (c 0.6, $CHCl_3$). m.p.= 114-115 ºC.

^{1}H NMR (300 MHz, $CDCl_3$) δ 7.92 (dd, J = 8.7, 1.1 Hz, 2H), 7.52 (s, 1H), 7.48 – 7.25 (m, 9H), 7.28 – 7.16 (m, 1H), 6.62 (d, J = 8.3 Hz, 1H), 5.09 (s, 1H), 4.84 (s, 1H), 4.08 (d, J = 12.5 Hz, 1H), 2.84 (d, J = 12.1 Hz, 1H), 2.42 (s, 3H), 2.19 (s, 3H), 1.30 (s, 9H). **^{13}C NMR (75 MHz, $CDCl_3$)** δ 175.4 (C), 169.5 (C), 164.0 (C), 149.0 (C), 140.8 (C), 137.7 (C), 135.5 (C), 131.7 (CH), 129.5 (C), 129.0 (C), 128.8 (CH), 127.8 (CH), 127.4 (CH), 125.9 (CH), 125.4 (CH), 121.7 (CH), 119.6 (CH), 119.0 (CH), 115.6 (C), 110.7 (C), 80.3 (C), 63.8 (C), 44.3 (CH_2), 42.3 (CH_2), 28.1 (CH_3), 26.1 (CH_3), 19.3 (CH_3). **HRMS (ESI/Q-TOF)** m/z: $[M+H]^+$ $C_{33}H_{34}BrN_4O_4^+$ Calcd for 629.1758; Found 629.1764.

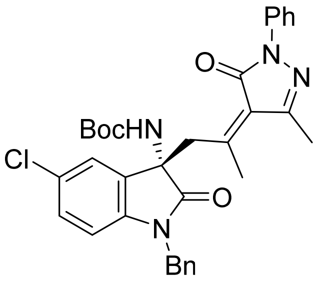

3ha

Following the General Procedure **iii**, product **3ha** was obtained as a yellow solid (41.6 mg, 71% yield) using as eluent mixtures of DCM/Et_2O 98:2 to DCM/Et_2O 90:10. Enantiomeric excess (93%) was determined by chiral HPLC (Chiralpak® ADH *i*PrOH/hexane = 20/80, flow rate= 1.0 mL/min, λ = 254 nm), t_R =17.60 min (major), 14.07 min (minor). [α_D^{20}]= -164.3 (c 0.4, $CHCl_3$). m.p.= 111-112 ºC.

^{1}H NMR (300 MHz, $CDCl_3$) δ 7.92 (dd, *J* = 8.8, 1.1 Hz, 2H), 7.52 (s, 1H, NH), 7.46 – 7.28 (m, 8H), 7.22 (t, *J* = 7.4 Hz, 1H), 7.15 (dd, *J* = 8.0, 1.9 Hz, 1H), 6.66 (d, *J* = 8.2 Hz, 1H), 5.09 (s, 1H), 4.84 (s, 1H), 4.08 (d, *J* = 12.5 Hz, 1H), 2.83 (d, *J* = 12.1 Hz, 1H), 2.41 (s, 3H), 2.19 (s, 3H), 1.30 (s, 9H). **^{13}C NMR (75 MHz, $CDCl_3$)** δ 175.5 (C), 164.0 (C), 162.3 (C), 154.2 (C), 149.0 (C), 140.3 (C), 137.7 (C), 135.5 (C), 133.3 (C), 129.4 (C), 128.79 (CH), 128.76 (CH), 128.3 (C), 127.7 (CH), 127.32 (CH), 127.26 (CH), 125.4 (CH), 123.1 (CH), 119.6 (CH), 110.2 (CH), 80.2 (C), 63.8 (C), 44.2 (CH_2), 42.2 (CH_2), 28.1 (CH_3), 26.0 (CH_3), 19.2 (CH_3). **HRMS (ESI/Q-TOF)** m/z: $[M+H]^+$ $C_{33}H_{34}ClN_4O_4^+$ Calcd for 585.2263; Found 585.2259.

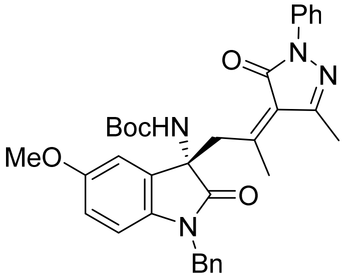

3ia

Following the General Procedure **iii**, product **3ia** was obtained as a yellow solid (44.3 mg, 76% yield) using as eluent mixtures of DCM/Et_2O 98:2 to DCM/Et_2O 90:10. Enantiomeric excess (96%) was determined by chiral HPLC (Chiralpak® ADH *i*PrOH/hexane = 20/80, flow rate= 1.0 mL/min, λ = 254 nm) t_R = 61.92 min (major), 24.01 min (minor). [α_D^{20}]= -168.0 (c 0.5, $CHCl_3$). m.p.= 114-115 ºC.

^{1}H NMR (300 MHz, $CDCl_3$) δ 7.92 (dd, *J* = 8.8, 1.1 Hz, 2H), 7.47 (s, 1H, NH), 7.46-7.26 (m, 7H), 7.25 – 7.17 (m, 1H), 7.02 (d, *J* = 2.4 Hz, 1H), 6.83-6.58 (m, 2H), 5.13 (s, 1H), 4.82 (s, 1H), 4.10 (d, *J* = 11.5 Hz, 1H), 3.73 (s, 3H), 2.88 (s, 1H), 2.41 (s, 3H), 2.19 (s, 3H), 1.25 (s, 9H). **^{13}C NMR (75 MHz, $CDCl_3$)** δ 175.7 (C), 164.1 (C), 156.2 (C), 154.2 (C), 149.1 (C), 137.8 (C), 136.1 (C), 135.1 (C), 129.2 (C), 128.9 (CH), 128.81 (CH), 128.75 (CH), 127.8 (C), 127.6 (CH), 127.4 (CH), 125.4 (CH), 119.6 (CH), 113.8 (CH), 109.7 (C), 109.4 (CH), 80.0 (C), 64.3 (C), 55.9 (CH_3), 44.3 (CH_2), 42.6 (CH_2), 28.1 (CH_3), 26.0 (CH_3), 19.3 (CH_3). **HRMS (ESI/Q-TOF)** m/z: $[M+H]^+$ $C_{34}H_{37}N_4O_5^+$ Calcd for 581.2758; Found 581.2763.

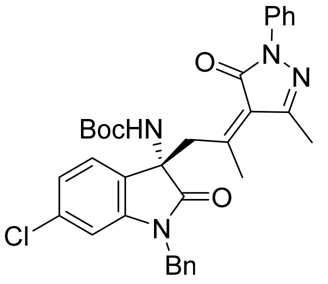

3ja

Following the General Procedure **iii**, product **3ja** was obtained as a yellow oil (38.0 mg, 65% yield) using as eluent mixtures of DCM/Et_2O 98:2 to DCM/Et_2O 90:10. Enantiomeric excess (94%) was determined by chiral HPLC (Lux® 5μm i-Amilose-1 *i*PrOH/hexane = 20/80, flow rate= 1.0 mL/min, λ = 254 nm), t_R = 16.84 min (major), 15.72 min (minor). [α_D^{20}]= -168.2 (c 0.5, $CHCl_3$).

^{1}H NMR (300 MHz, $CDCl_3$) δ 7.91 (dd, *J* = 8.8, 1.1 Hz, 2H), 7.47 (s, 1H, NH), 7.46-7.28 (m, 8H), 7.22 (t, *J* = 7.4 Hz, 1H), 7.01 (dd, *J* = 7.9, 1.6 Hz, 1H), 6.76 (d, *J* = 1.5 Hz, 1H), 5.14 (s, 1H), 4.79 (s, 1H), 4.05 (d, *J* = 12.7 Hz, 1H), 2.87 (d, *J* = 9.8 Hz, 1H), 2.42 (s, 3H), 2.18 (s, 3H), 1.26 (s, 9H). **^{13}C NMR (75 MHz, $CDCl_3$)** δ 175.9 (C), 164.0 (C), 162.4 (C), 154.2 (C), 149.0 (C), 143.1 (C), 137.8 (C), 135.5 (C), 134.6 (C), 130.3 (C), 129.4 (C), 128.9 (CH), 128.8 (CH), 127.8 (CH), 127.4 (CH), 125.4 (CH), 123.5 (CH), 122.7 (CH), 119.6 (CH), 109.8 (CH), 80.2 (C), 63.4 (C), 44.3 (CH_2), 42.2 (CH_2), 28.1 (CH_3), 26.0 (CH_3), 19.3 (CH_3). **HRMS (ESI/Q-TOF)** m/z: $[M+H]^+$ $C_{33}H_{34}ClN_4O_4^+$ Calcd for 585.2263; Found 585.2253.

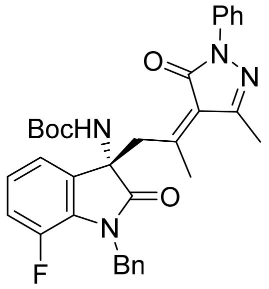

3ka

Following the General Procedure **iii**, product **3ka** was obtained as a yellow solid (42.8 mg, 75% yield) using as eluent mixtures of DCM/Et_2O 98:2 to DCM/Et_2O 90:10. Enantiomeric excess (94%) was determined by chiral HPLC (Chiralpak® ADH *i*PrOH/hexane = 20/80, flow rate= 1.0 mL/min, λ = 254 nm), t_R = 29.49 min (major), 14.74 min (minor). [α_D^{20}]= -101.1 (c 0.5, $CHCl_3$). m.p.= 207-208 ºC.

^{1}H NMR (300 MHz, $CDCl_3$) δ 7.91 (dd, *J* = 8.8, 1.1 Hz, 2H), 7.56 (s, 1H, NH), 7.50-7.28 (m, 7H), 7.24-7.15 (m, 2H), 7.11-6.94 (m, 2H), 5.19 (s, 1H), 5.05 (s, 1H), 4.08 (d, *J* = 12.5 Hz, 1H), 2.74 (d, *J* = 12.6 Hz, 1H), 2.40 (s, 3H), 2.09 (s, 3H), 1.26 (s, 9H). **^{13}C NMR (75 MHz, $CDCl_3$)** δ 175.6 (C), 164.0 (C), 162.8 (C), 154.2 (C), 149.0 (C), 145.8 (C), 137.7 (C), 137.2 (C), 134.8 (C), 129.4 (C), 128.8 (CH), 128.5 (CH), 127.8 (CH), 127.5 (CH), 125.4 (CH), 123.5 (d, *J* = 6.6 Hz), 119.6 (CH), 118.4 (d, *J* = 1.7 Hz), 117.12 (d, *J* = 19.4 Hz), 80.2 (C), 64.0 (C), 45.8 (CH_2), 42.4 (CH_2), 28.1 (CH_3), 26.0 (CH_3), 19.2 (CH_3).**^{19}F NMR (282 MHz, $CDCl_3$)** δ -133.59. **HRMS (ESI/Q-TOF)** m/z: $[M+H]_+$ $C_{33}H_{34}FN_4O_4^+$ Calcd for 569.2559; Found 569.2551.

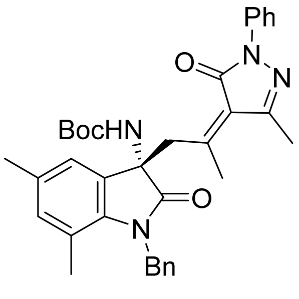

3la

Following the General Procedure **iii**, product **3la** was obtained as a yellow solid (38.1 mg, 66% yield) using as eluent mixtures of DCM/Et_2O 98:2 to DCM/Et_2O 90:10. Enantiomeric excess (98%) was determined by chiral HPLC (Lux® 5μm i-Amilose-1 *i*PrOH/hexane = 20/80, flow rate= 1.0 mL/min, λ = 254 nm), t_R = 22.50 min (major), 17.03 min (minor). $[\alpha_D^{20}]$= -159.1 (c 0.2, $CHCl_3$). m.p.= 119-120 ºC.

^{1}H NMR (300 MHz, $CDCl_3$) δ 7.94 (dd, *J* = 8.8, 1.1 Hz, 2H), 7.62 (s, 1H, NH), 7.47-7.39 (m, 2H), 7.37-7.29 (m, 4H), 7.22 (t, *J* = 7.4 Hz, 2H), 7.11 (s, 1H), 6.77 (s, 1H), 5.24 (s, 2H), 4.21 (s, 1H), 2.69 (s, 1H), 2.41 (s, 3H), 2.26 (s, 3H), 2.24 (s, 3H), 2.18 (s, 3H), 1.30 (s, 9H). **^{13}C NMR (75 MHz, $CDCl_3$)** δ 176.6 (C), 164.2 (C), 163.8 (C), 154.2 (C), 149.2 (C), 138.1 (C), 137.8 (C), 137.2 (C), 133.3 (CH), 132.7 (C), 132.4 (C), 129.2 (C), 128.8 (CH), 128.7 (CH), 127.1 (CH), 125.8 (CH), 125.3 (CH), 121.0 (CH), 119.6 (CH), 119.4 (C), 79.8 (C), 63.7 (C), 45.3 (CH_2), 43.3 (CH_2), 28.1 (CH_3), 26.2 (CH_3), 20.7 (CH_3), 19.3 (CH_3), 18.6 (CH_3). **HRMS (ESI/Q-TOF)** m/z: $[M+H]^+$ $C_{35}H_{39}N_4O_4^+$ Calcd for 579.2966; Found 579.2973.

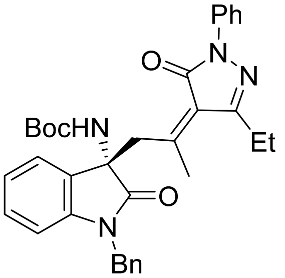

3bb

Following the General Procedure **iii**, product **3bb** was obtained as a yellow solid (53.1 mg, 94% yield) using as eluent mixtures of DCM/Et_2O 98:2 to DCM/Et_2O 90:10. Enantiomeric excess (95%) was determined by chiral HPLC (Chiralpak® ADH *i*PrOH/hexane = 20/80, flow rate= 1.0 mL/min, λ = 254 nm), t_R = 24.70 min (major), 18.15 min (minor). $[\alpha_D^{20}]$= -141.1 (c 0.6, $CHCl_3$). m.p.= 102-103 ºC.

^{1}H NMR (300 MHz, $CDCl_3$) δ 7.96 (dd, *J* = 8.7, 1.2 Hz, 2H), 7.49 (s, 1H, NH), 7.47-7.27 (m, 8H), 7.25-7.15 (m, 2H), 7.02 (t, *J* = 7.4 Hz, 1H), 6.77 (d, *J* = 7.7 Hz, 1H), 5.15 (s, 1H), 4.79 (s, 1H), 4.05 (d, *J* = 11.4 Hz, 1H), 2.96 (s, 1H), 2.87-2.61 (m, 2H), 2.15 (s, 3H), 1.33 (t, *J* = 7.2 Hz, 3H), 1.26 (s, 9H). **^{13}C NMR (75 MHz, $CDCl_3$)** δ 176.0 (C), 164.3 (C), 162.2 (C), 154.2 (C), 153.2 (C), 141.8 (C), 138.0 (C), 136.0 (C), 131.6 (C), 128.9 (CH), 128.8 (CH), 128.7 (CH), 127.6 (CH), 127.4 (CH), 127.3 (C), 125.2 (CH), 122.7 (CH), 122.6 (CH), 119.5 (CH), 109.1 (CH), 79.9 (C), 63.8 (C), 44.1 (CH_2), 42.5 (CH_2), 28.1 (CH_3), 26.2 (CH_3), 25.8 (CH_2), 10.7 (CH_3). **HRMS (ESI/Q-TOF)** m/z: $[M+H]^+$ $C_{34}H_{37}N_4O_4^+$ Calcd for 565.2809; Found 565.2801.

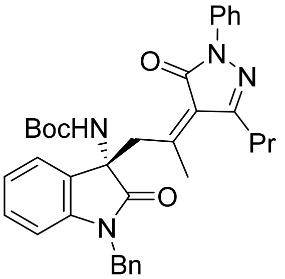

3bc

Following the General Procedure **iii**, product **3bc** was obtained as a yellow solid (48.7 mg, 84% yield) using as eluent mixtures of DCM/Et_2O 98:2 to DCM/Et_2O 90:10. Enantiomeric excess (96%) was determined by chiral HPLC (Lux® 5μm i-amilose-1 *i*PrOH/hexane = 20/80, flow rate= 1.0 mL/min, λ = 254 nm), t_R = 21.38 min (major), 14.61 min (minor). $[\alpha_D^{20}]$= -143.7 (c 0.6, $CHCl_3$). m.p.= 98-99 ºC.

^{1}H NMR (400 MHz, CDCl$_3$) δ 7.94 (dd, *J* = 8.8, 1.1 Hz, 2H), 7.48 (s, 1H), 7.45-7.27 (m, 9H), 7.25-7.15 (m, 2H), 7.02 (t, *J* = 7.5 Hz, 1H), 6.77 (d, *J* = 7.7 Hz, 1H), 5.16 (s, 1H), 4.82 (s, 1H), 4.01 (s, 1H), 3.01 (s, 1H), 2.75-2.59 (m, 2H), 2.14 (s, 3H), 1.78 (h, *J* = 7.5 Hz, 2H), 1.26 (s, 9H), 1.05 (t, *J* = 7.3 Hz, 3H). **^{13}C NMR (75 MHz, CDCl$_3$)** δ 176.1 (C), 164.2 (C), 162.3 (C), 154.2 (C), 152.1 (C), 141.8 (C), 138.0 (C), 136.0 (C), 131.5 (C), 128. (CH), 128.8 (CH), 128.7 (CH), 127.6 (CH), 127.4 (CH), 125.2 (CH), 122.7 (CH), 122.6 (CH), 122.0 (C), 119.5 (CH), 109.1 (CH), 79.9 (C), 63.8 (C), 44.1 (CH$_2$), 42.4 (CH$_2$), 34.3 (CH$_2$), 28.0 (CH$_3$), 26.1 (CH$_3$), 20.0 (CH$_2$), 13.9 (CH$_3$). **HRMS (ESI/Q-TOF)** m/z: [M+H]$^+$ $C_{35}H_{39}N_4O_4^+$ Calcd for 579.2966; Found 579.2952.

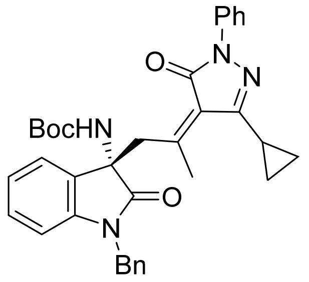

3bd

Following the General Procedure **iii**, product **3bd** was obtained as a yellow solid (56.6 mg, 98% yield) using as eluent mixtures of DCM/Et$_2$O 98:2 to DCM/Et$_2$O 90:10. Enantiomeric excess (96%) was determined by chiral HPLC (Chiralpak® ADH *i*PrOH/hexane = 20/80, flow rate= 1.0 mL/min, λ = 254 nm), t_R = 26.69 min (major), 14.79 min (minor). [α_D^{20}]= -139.7 (c 0.8, CHCl$_3$). m.p.= 104-105 ºC.

^{1}H NMR (300 MHz, CDCl$_3$) δ 7.93 (dd, *J* = 8.8, 1.1 Hz, 2H), 7.48 (s, 1H, NH), 7.45-7.26 (m, 9H), 7.20 (t, *J* = 7.4 Hz, 2H), 7.02 (t, *J* = 7.6 Hz, 1H), 6.77 (d, *J* = 7.8 Hz, 1H), 5.18 (s, 1H), 4.80 (s, 1H), 3.99 (d, *J* = 11.8 Hz, 1H), 3.06 (s, 1H), 2.34 (s, 3H), 1.92-1.65 (m, 1H), 1.26 (s, 9H), 1.01-0.86 (m, 4H). **^{13}C NMR (75 MHz, CDCl$_3$)** δ 176.1 (C), 164.1 (C), 163.1 (C), 154.2 (C), 152.9 (C), 141.8 (C), 138.0 (C), 136.0 (C), 131.6 (C), 129.2 (C), 128.8 (CH), 128.7 (CH), 128.7 (CH), 127.6 (CH), 127.4 (CH), 125.2 (CH), 122.7 (CH), 122.6 (CH), 119.4 (C), 109.1 (C), 79.9 (C), 63.8 (C), 44.1 (CH$_2$), 42.5 (CH$_2$), 28.1 (CH$_3$), 26.3 (CH$_3$), 12.9 (CH), 7.3 (CH$_2$), 6.4 (CH$_2$). **HRMS (ESI/Q-TOF)** m/z: [M+H]$^+$ $C_{35}H_{37}N_4O_4^+$ Calcd for 577.2809; Found 577.2812.

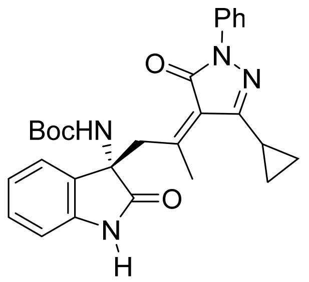

3fd

Following the General Procedure **iii**, product **3fd** was obtained as a yellow solid (38.1 mg, 78% yield) using as eluent mixtures of DCM/Et$_2$O 98:2 to DCM/Et$_2$O 90:10. Enantiomeric excess (94%) was determined by chiral HPLC (Chiralpak® ADH *i*PrOH/hexane = 20/80, flow rate= 1.0 mL/min, λ = 254 nm) t_R = 13.29 min (major), 9.61 min (minor). [α_D^{20}]= -164.4 (c 0.7, CHCl$_3$). m.p.= 122-123 ºC.

^{1}H NMR (300 MHz, CDCl$_3$) δ 8.30 (s, 1H, NH), 7.91 (dd, *J* = 8.8, 1.1 Hz, 2H), 7.53 (s, 1H, NH), 7.40 (t, *J* = 8.0 Hz, 2H), 7.33 (d, *J* = 7.5 Hz, 1H), 7.20 (t, *J* = 7.4 Hz, 2H), 7.01 (td, *J* = 7.5, 0.9 Hz, 1H), 6.84 (d, *J* = 7.5 Hz, 1H), 3.96 (d, *J* = 12.5 Hz, 1H), 3.06 (d, *J* = 12.5 Hz, 1H), 2.43 (s, 3H), 1.95-1.79 (m, 1H), 1.26 (s, 9H), 1.06-0.74 (m, 4H). **^{13}C NMR (75 MHz, CDCl$_3$)** δ 178.1 (C), 164.1 (C), 163.4 (C), 154.4 (C), 153.0 (C), 139.9 (C), 137.9 (C), 131.9 (C), 129.1 (C), 128.9 (CH), 128.7 (CH), 125.2 (CH), 122.8 (CH), 122.6 (CH), 119.5 (CH), 110.2 (CH), 80.2 (C), 64.0 (C), 42.3 (CH$_2$), 28.1 (CH$_3$), 26.2 (CH$_3$), 12.9 (C$_H$), 7.3 (CH$_2$), 6.5 (CH$_2$). **HRMS (ESI/Q-TOF)** m/z: [M+H]$^+$ $C_{28}H_{31}N_4O_4^+$ Calcd for 487.2345; Found 487.2340.

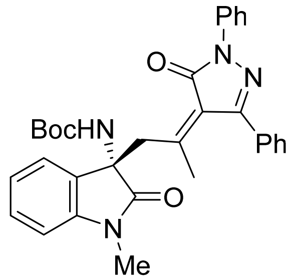

3ae

Following the General Procedure **iii**, product **3ae** was obtained as a yellow solid (36.5 mg, 68% yield) using as eluent mixtures of DCM/Et_2O 98:2 to DCM/Et_2O 90:10. Enantiomeric excess (94%) was determined by chiral HPLC (Chiralpak® ADH *i*PrOH/hexane = 20/80, flow rate= 1.0 mL/min, λ = 254 nm), t_R = 32.40 min (major), 24.25 min (minor). $[\alpha_D^{20}]$= -151.5 (c 0.4, $CHCl_3$). m.p.= 128-129 ºC.

^{1}H NMR (300 MHz, $CDCl_3$) δ 7.98 (dd, *J* = 8.8, 1.3 Hz, 2H), 7.57-7.34 (m, 9H), 7.31 (td, *J* = 7.8, 1.3 Hz, 1H), 7.23 (t, *J* = 7.4 Hz, 1H), 7.07 (t, *J* = 7.5 Hz, 1H), 6.86 (d, *J* = 7.7 Hz, 1H), 3.93 (s, 1H), 3.26 (s, 3H), 3.04 (s, 1H), 1.84 (s, 3H), 1.25 (s, 9H). **^{13}C NMR (75 MHz, $CDCl_3$)** δ 175.8 (C), 165.1 (C), 163.9 (C), 154.2 (C), 152.0 (C), 148.2 (C), 142.6 (C), 137.9 (C), 133.6 (C), 129.5 (CH), 129.0 (CH), 128.8 (CH), 128.72 (CH), 128.69 (CH), 128.2 (C), 125.5 (CH), 122.7 (CH), 122.6 (CH), 119.8 (CH), 108.1 (CH), 80.0 (C), 63.7 (C), 42.1 (CH_2), 28.0 (CH_3), 27.6 (CH_3), 26.5 (CH_3). **HRMS (ESI/Q-TOF)** m/z: $[M+H]^+$ $C_{32}H_{33}N_4O_4^+$ Calcd for 537.2496; Found 537.2502.

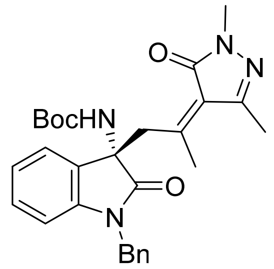

3bf

Following the General Procedure **iii**, product **3bf** was obtained as a yellow solid (35.6 mg, 73% yield) using as eluent mixtures of DCM/Et_2O 98:2 to DCM/Et_2O 90:10. Enantiomeric excess (99.5%) was determined by chiral HPLC (Chiralpak® ADH) *i*PrOH/hexane = 20/80, flow rate= 1.0 mL/min, λ = 254 nm), t_R = 27.43 min (major), 19.45 min (minor). $[\alpha_D^{20}]$= -92.0 (c 0.5, $CHCl_3$). m.p.= 123-124 ºC.

^{1}H NMR (300 MHz, $CDCl_3$) δ 7.62 (s, 1H), 7.39-7.21 (m, 6H), 7.17-7.05 (m, 1H), 7.00-6.90 (m, 1H), 6.68 (d, *J* = 8.1 Hz, 1H), 5.09 (s, 1H), 4.76 (s, 1H), 3.98 (d, *J* = 11.6 Hz, 1H), 3.30 (s, 3H), 2.66 (d, *J* = 13.5 Hz, 1H), 2.24 (s, 3H), 2.04 (s, 3H), 1.18 (s, 9H). **^{13}C NMR (75 MHz, $CDCl_3$)** δ 176.0 (C), 165.2 (C), 162.2 (C), 154.2 (C), 147.7 (C), 141.8 (C), 136.1 (C), 132.0 (C), 129.2 (C), 128.8 (CH), 128.7 (CH), 127.6 (CH), 127.4 (CH), 122.7 (CH), 122.3 (CH), 109.0 (CH), 79.8 (C), 63.9 (C), 44.1 (CH_2), 42.5 (CH_2), 31.2 (CH_3), 28.1 (CH_3), 25.9 (CH_3), 19.0 (CH_3). **HRMS (ESI/Q-TOF)** m/z $[M+H]^+$ $C_{28}H_{33}N_4O_4^+$ Calcd for 489.2496; Found 489.2504.

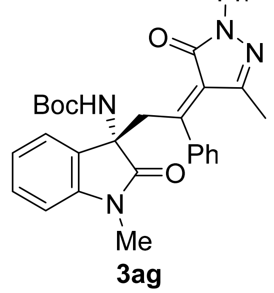

3ag

Following the General Procedure **iii**, product **3ag** was obtained as a yellow solid (43.6 mg, 81% yield) using as eluent mixtures of DCM/Et_2O 98:2 to DCM/Et_2O 90:10. Enantiomeric excess (96%) was determined by chiral HPLC (Chiralpak® ADH *i*PrOH/hexane = 20/80, flow rate= 1.0 mL/min, λ = 254 nm), t_R = 23.35 min (major), 10.10 min (minor). $[\alpha_D^{20}]$= -407.0 (c 0.7, $CHCl_3$). m.p.= 107-108 ºC.

^{1}H NMR (300 MHz, $CDCl_3$) δ 7.89 (dd, *J* = 8.8, 1.3 Hz, 2H), 7.49-7.36 (m, 7H), 7.25 (td, *J* = 7.7, 1.3 Hz, 2H), 7.18 (ddt, *J* = 7.9, 7.0, 1.2 Hz, 1H), 6.66 (d, *J* = 7.7 Hz, 1H), 5.42 (s, 1H), 4.66 (d, *J* = 12.8 Hz, 1H), 3.49 (d, *J* = 13.0 Hz, 1H), 2.77 (s, 3H), 1.44 (s, 3H), 1.17 (s, 9H). **^{13}C NMR (75 MHz, $CDCl_3$)** δ 174.9 (C), 163.2 (C), 161.3 (C), 153.4 (C), 148.4 (C), 143.4 (C), 138.0 (C), 137.7 (C), 130.3 (CH), 129.8 (C), 129.1 (CH), 128.8 (CH), 127.7 (CH), 125.0 (CH), 123.4 (CH), 122.5 (CH), 119.0 (CH), 108.0 (C), 80.0 (C), 62.2 (C), 39.4 (CH_2), 28.0 (CH_3), 26.0 (CH_3), 17.3 (CH_3). **HRMS (ESI/Q-TOF)** m/z: $[M+H]^+$ $C_{32}H_{33}N_4O_4^+$ Calcd for 537.2496; Found 537.2504.

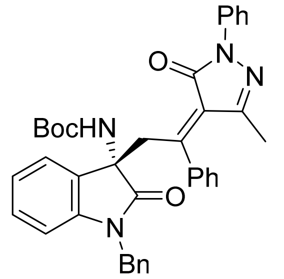

3bg

Following the General Procedure **iii**, product **3bg** was obtained as a yellow solid (44.6 mg, 73% yield) using as eluent mixtures of DCM/Et_2O 98:2 to DCM/Et_2O 90:10. Enantiomeric excess (96%) was determined by chiral HPLC (Chiralpak® ADH *i*PrOH/hexane = 20/80, flow rate= 1.0 mL/min, λ = 254 nm), t_R = 20.16 min (major), 9.79 min (minor). $[\alpha_D^{20}]$= -179.7 (c 0.5, $CHCl_3$). m.p.= 121-122 ºC.

^{1}H NMR (300 MHz, $CDCl_3$) δ 7.92 (dd, *J* = 8.8, 1.1 Hz, 2H), 7.51-7.37 (m, 8H), 7.25-7.16 (m, 6H), 7.13 (td, *J* = 7.7, 1.3 Hz, 1H), 7.02 (s, 1H), 6.96 (td, *J* = 7.6, 1.0 Hz, 1H), 6.57 (d, *J* = 7.7 Hz, 1H), 4.72-4.20 (m, 3H), 3.81 (d, *J* = 13.0 Hz, 1H), 1.50 (s, 3H), 1.29-1.10 (m, 9H**)**. **^{13}C NMR (75 MHz, $CDCl_3$)** δ 175.1 (C), 163.3, 161.6 (C), 153.6 (C), 148.7 (C), 142.4 (C), 138.6 (C), 138.0 (C), 135.6 (C), 134.6 (C), 130.6 (C), 130.1 (CH), 129.2 (C), 128.9 (CH), 128.8 (CH), 128.5 (CH), 128.0 (CH), 127.5 (CH) 127.33 (CH), 127.25 (CH), 125.0 (CH), 123.2 (CH), 122.6 (CH), 119.1 (CH), 109.2 (CH), 79.9 (C), 62.9 (C), 45.0 (CH_2), 44.1 (CH_2), 28.0 (CH_3), 17.4 (CH_3). **HRMS (ESI/Q-TOF)** m/z: $[M+H]^+$ $C_{38}H_{37}N_4O_4^+$ Calcd for 613.2809; Found 613.2798.

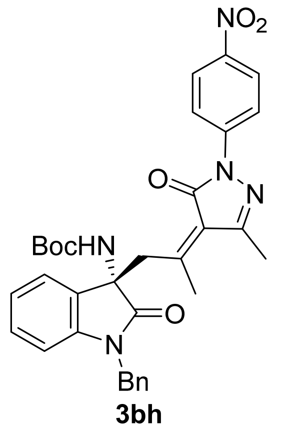

3bh

Following the General Procedure **iii**, product **3bh** was obtained as a yellow solid (42.9 mg, 72% yield) using as eluent mixtures of DCM/Et_2O 98:2 to DCM/Et_2O 90:10. Enantiomeric excess (97%) was determined by chiral HPLC (Chiralpak® IC, *i*PrOH/hexane = 20/80, flow rate= 1.0 mL/min, λ = 254 nm), t_R= 30.57 min (major), 23.75 min (minor). $[\alpha_D^{20}]$= -10.6 (c 0.5, $CHCl_3$). m.p.= 126-127 ºC.

^{1}H NMR (300 MHz, CDCl$_3$) δ 8.27 (d, *J* = 9.5 Hz, 2H), 8.20 (d, *J* = 9.7 Hz, 2H), 7.44 -7.26 (m, 6H), 7.24-7.17 (m, 1H), 7.08 (s, 1H), 7.04 (td, *J* = 7.6, 0.8 Hz, 1H), 6.79 (d, *J* = 7.5 Hz, 1H), 5.14 (s, 1H), 4.75 (s, 1H), 4.02 (d, *J* = 12.3 Hz, 1H), 3.04 (d, *J* = 12.3 Hz, 1H), 2.43 (s, 3H), 2.21 (s, 3H), 1.25 (s, 9H). **^{13}C NMR (75 MHz, CDCl$_3$)** δ 175.7 (C), 165.1 (C), 164.5 (C), 154.1 (C), 150.5 (C), 143.9 (C), 142.9 (C), 141.9 (C), 135.8 (C), 131.3 (C), 129.1 (C), 128.8 (CH), 128.5 (CH), 127.7 (CH), 127.4 (CH), 124.7 (CH), 122.8 (CH), 122.7 (CH), 118.2 (CH), 109.2 (CH), 80.2 (C), 63.8 (C), 44.2 (CH$_2$), 42.4 (CH$_2$), 28.0 (CH$_3$), 26.1 (CH$_3$), 19.2 (CH$_3$). **HRMS (ESI/Q-TOF)** m/z: $[M+H]^+$ $C_{33}H_{34}N_5O_6^+$ Calcd for 596.2504; Found 596.2394.

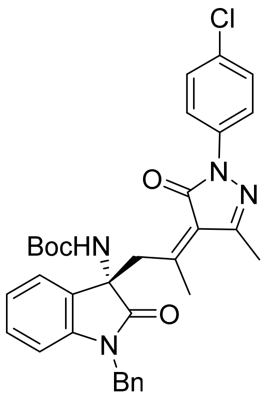

3bi

Following the General Procedure **iii**, product **3bi** was obtained as a yellow solid (48.5 mg, 83% yield) using as eluent mixtures of DCM/Et$_2$O 98:2 to DCM/Et$_2$O 90:10. Enantiomeric excess (96%) was determined by chiral HPLC (Lux® 5µm i-Amilose-1 *i*PrOH/hexane = 20/80, flow rate= 1.0 mL/min, λ = 254 nm), t_R = 25.25 min (major), t_r = 22.81 min (minor). $[\alpha_D^{20}]$= -125.7 (c 0.3, CHCl$_3$). m.p.= 162-163 ºC.

^{1}H NMR (300 MHz, CDCl$_3$) δ 7.92 (d, *J* = 9.0 Hz, 2H), 7.44-7.27 (m, 9H), 7.20 (td, *J* = 7.9, 1.2 Hz, 1H), 7.03 (t, *J* = 7.3 Hz, 1H), 6.78 (d, *J* = 7.9 Hz, 1H), 5.18 (s, 1H), 4.81 (s, 1H), 4.06 (d, *J* = 12.3 Hz, 1H), 2.90 (d, *J* = 10.6 Hz, 1H), 2.40 (s, 3H), 2.17 (s, 3H), 1.26 (s, 9H). **^{13}C NMR (75 MHz, CDCl$_3$)** δ 175.9 (C), 164.0 (C), 163.7 (C), 154.1 (C), 149.3 (C), 141.8 (C), 136.4 (C), 136.0 (C), 131.5 (C), 130.3 (C), 129.0 (C), 128.92 (CH), 128.79 (CH), 128.75 (CH), 127.6 (CH), 127.4 (CH), 122.8 (CH), 122.5 (CH), 120.4 (CH), 109.1 (CH), 80.0 (C), 63.9 (C), 44.1 (CH$_2$), 42.45 (CH$_2$), 28.0 (CH$_3$), 26.1 (CH$_3$), 19.2 (CH$_3$). **HRMS (ESI/Q-TOF)** m/z: $[M+H]^+$ $C_{33}H_{34}ClN_4O_4^+$ Calcd for 585.2263; Found 584.2252.

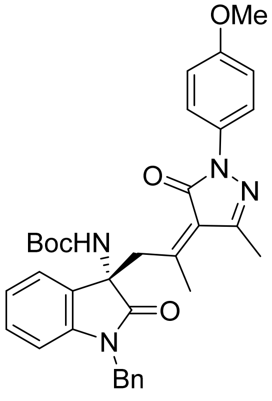

3bj

Following the General Procedure **iii**, product **3bj** was obtained as a yellow solid (36.5 mg, 63% yield) using as eluent mixtures of DCM/Et$_2$O 98:2 to DCM/Et$_2$O 90:10. Enantiomeric excess (93%) was determined by chiral HPLC (Chiralcel® ODH *i*PrOH/hexane = 20/80, flow rate= 1.0 mL/min, λ = 254 nm), t_R = 15.75 min (major), 22.79 min (minor). $[\alpha_D^{20}]$= -122.8 (c 0.3, CHCl$_3$). m.p.= 128-129 ºC.

^{1}H NMR (300 MHz, $CDCl_3$) δ 7.79 (d, *J* = 9.2 Hz, 2H), 7.60 (s, 1H), 7.43-7.27 (m, 6H), 7.19 (t, *J* = 7.6 Hz, 1H), 7.02 (t, *J* = 7.2 Hz, 1H), 6.95 (d, *J* = 9.2 Hz, 2H), 6.76 (d, *J* = 7.9 Hz, 1H), 5.18 (s, 1H), 4.78 (s, 1H), 4.11 (d, *J* = 12.5 Hz, 1H), 3.82 (s, 1H), 2.82 (d, *J* = 12.1 Hz, 1H), 2.40 (s, 3H), 2.17 (s, 3H), 1.26 (s, 3H). **^{13}C NMR (75 MHz, $CDCl_3$)** δ 176.0 (C), 163.7 (C), 162.9 (C), 157.2 (C), 154.2 (C), 148.8 (C), 141.8 (C), 136.0 (C), 131.7 (C), 131.1 (C), 129.2 (C), 128.8 (CH), 128.7 (CH), 127.6 (CH), 127.4 (CH), 122.8 (CH), 122.5 (CH), 121.5 (CH), 114.0 (CH), 109.1 (CH), 79.4 (C), 63.9 (C), 55.4 (CH_3), 44.1 (CH_2), 42.5 (CH_2), 28.1 (CH_3), 26.1 (CH_3), 19.2 (CH_3). **HRMS (ESI/Q-TOF)** m/z: $[M+H]^+$ $C_{34}H_{37}N_4O_5^+$ Calcd for 581.2758; Found 581.2749.

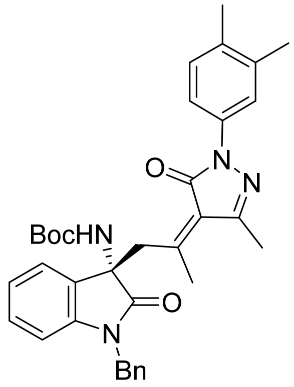

3bk

Following the General Procedure **iii**, product **3bk** was obtained as a yellow solid (50.9 mg, 90% yield) using as eluent mixtures of DCM/Et_2O 98:2 to DCM/Et_2O 90:10. Enantiomeric excess (95%) was determined by chiral HPLC (Chiralpak® ADH *i*PrOH/hexane = 20/80, flow rate= 1.0 mL/min, λ = 254 nm), t_R = 22.29 min (major), 15.09 min (minor). $[\alpha_D^{20}]$= -117.6 (c 0.3, $CHCl_3$). m.p.= 117-118 ºC.

^{1}H NMR (300 MHz, $CDCl_3$) δ 7.70-7.59 (m, 3H), 7.47-7.26 (m, 6H), 7.22-7.13 (m, 2H), 7.03 (t, *J* = 7.2 Hz, 1H), 6.77 (d, *J* = 7.7 Hz, 1H), 5.18 (s, 1H), 4.81 (s, 1H), 4.11 (d, *J* = 12.5 Hz, 1H), 2.84 (d, *J* = 11.7 Hz, 1H), 2.40 (s, 3H), 2.31 (s, 3H), 2.27 (s, 3H), 2.16 (s, 3H), 1.26 (s, 9H). **^{13}C NMR (75 MHz, $CDCl_3$)** δ 176.0 (C), 163.9 (C), 162.7 (C), 154.3 (C), 148.8 (C), 141.9 (C), 137.1 (C), 136.1 (C), 135.7 (C), 133.9 (C), 132.0 (C), 129.9 (CH), 129.4 (C), 128.9 (CH), 128.8 (CH), 127.6 (CH), 127.5 (CH), 122.8 (CH), 122.6 (CH), 120.9 (CH), 117.4 (CH), 109.1 (CH), 79.9 (C), 64.0 (C), 44.2 (CH_2), 42.6 (CH_2), 28.1 (CH_3), 26.1 (CH_3), 20.0 (CH_3), 19.4 (CH_3), 19.3 (CH_3). **HRMS (ESI/Q-TOF)** m/z: $[M+H]^+$ $C_{35}H_{39}N_4O_4^+$ Calcd for 579.2966; Found 579.2974.

<u>CHARACTERITZATION OF THE PRODUCT 4</u> <u>(transformation i)</u>

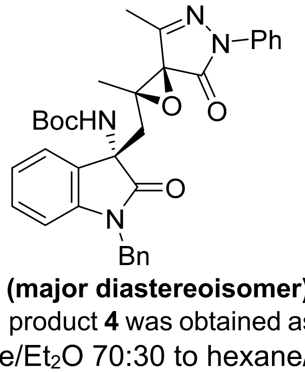

4 (major diastereoisomer)

Following the General Procedure **iv**, product **4** was obtained as a white solid (35.6 mg, 86% yield) using as eluent mixtures of hexane/Et_2O 70:30 to hexane/Et_2O 40:60. Enantiomeric excess (96%) was determined by chiral HPLC (Chiralpak® ADH *i*PrOH/hexane = 20/80, flow rate= 1.0 mL/min, λ = 254 nm) t_R = 21.74 min (major), 9.96 min (minor). $[\alpha_D^{20}]$= -119.2 (c 0.5, $CHCl_3$). m.p.= 182-183 ºC.

^{1}H NMR (300 MHz, $CDCl_3$) δ 7.90 (dd, *J* = 8.7, 1.1 Hz, 2H), 7.48-7.39 (m, 2H), 7.35 (d, *J* = 7.2 Hz, 1H), 7.32-7.18 (m, 6H), 7.12 (td, *J* = 7.8, 1.2 Hz, 1H), 6.86 (td, *J* = 7.6, 0.9 Hz, 1H), 6.67 (d,

J = 7.7 Hz, 1H), 5.76 (s, 1H), 4.76 (d, J = 14.8 Hz, 1H), 4.63 (d, J = 14.0 Hz, 1H), 3.05 (d, J = 14.8 Hz, 1H), 2.63 (d, J = 14.7 Hz, 1H), 2.13 (s, 3H), 1.67 (s, 3H), 1.31 (s, 9H). **^{13}C NMR (75 MHz, CDCl$_3$)** δ 175.7 (C), 167.7 (C), 155.9 (C), 153.7 (C), 141.8 (C), 138.0 (C), 135.5 (C), 130.7 (C), 128.9 (CH), 128.6 (CH), 127.5 (CH), 127.5 (CH), 125.4 (CH), 123.3 (CH), 122.1 (CH), 118.8 (CH), 109.3 (CH), 80.2 (C), 68.3 (C), 65.5 (C), 60.1 (C), 44.1 (CH$_2$), 28.2 (CH$_3$), 22.0 (CH$_3$), 16.8 (CH$_3$). **HRMS (ESI/Q-TOF)** m/z: $[M+H]^+$ $C_{33}H_{35}N_4O_5^+$ Calcd for 567.2602; Found 567.2591.

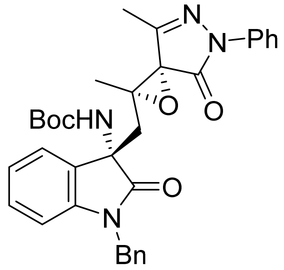

4 (minor diastereoisomer)

Following the General Procedure **iv**, product **4** was obtained as a white solid (3.7 mg, 9% yield) using as eluent mixtures of hexane/Et$_2$O 70:30 to hexane/Et$_2$O 40:60. Enantiomeric excess (98%) was determined by chiral HPLC (Chiralpak® ADH *i*PrOH/hexane = 20/80, flow rate= 1.0 mL/min, λ = 254 nm), t_R = 30.43 min (major), 16.40 min (minor). $[\alpha_D^{20}]$= +28.6 (c 0.5, CHCl$_3$). m.p.= 178-179 ºC.

^{1}H NMR (400 MHz, CDCl$_3$) δ 7.75 (d, J = 7.9 Hz, 2H), 7.48-7.33 (m, 7H), 7.20 (t, J = 7.4 Hz, 1H), 7.05-6.90 (m, 2H), 6.65 (d, J = 7.2 Hz, 1H), 6.40 (s, 1H), 5.16 (d, J = 15.9 Hz, 1H), 4.76 (d, J = 14.8 Hz, 1H), 2.89 (d, J = 15.0 Hz, 1H), 2.41 (d, J = 15.0 Hz, 1H), 2.22 (s, 3H), 1.56 (s, 3H), 1.28 (s, 9H).**^{13}C NMR (101 MHz, CDCl$_3$)** δ 177.1 (C), 167.4 (C), 155.4 (C), 153.9 (C), 142.5 (C), 137.7 (C), 135.8 (C), 129.0 (CH), 128.8 (C), 128.7 (CH), 127.5 (CH), 127.4 (C), 125.34 (CH), 123.63 (C), 123.55 (CH), 122.4 (CH), 118.6 (CH), 109.5 (CH), 80.1 (C), 68.8 (C), 65.4 (C), 60.7 (C), 44.1 (CH$_2$), 37.1 (CH$_2$), 28.1 (CH$_3$), 23.5 (CH$_3$), 16.8 (CH$_3$). **HRMS (ESI/Q-TOF)** m/z: $[M+H]^+$ $C_{33}H_{35}N_4O_5^+$ Calcd for 567.2602; Found 567.2612.

<u>CHARACTERITZATION OF THE PRODUCT 5</u> <u>(transformation ii)</u>

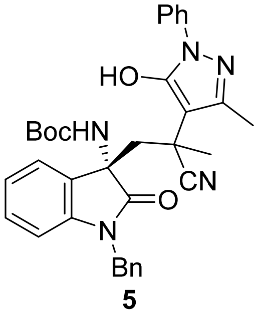

5

Following the General Procedure **v**, product **5** was obtained as a white solid (30.8 mg, 73% yield) using as eluent mixtures of DCM/Et$_2$O 95:5 to DCM/Et$_2$O 90:10. Enantiomeric excess (96%) was determined by chiral HPLC (Chiralpak® IC *i*PrOH/hexane = 20/80, flow rate= 1.0 mL/min, λ = 254 nm), t_R = 20.65 min (major), 9.34 min (minor). $[\alpha_D^{20}]$= +13.1 (c 0.1, CHCl$_3$). m.p.= 72-73 ºC.

^{1}H NMR (500 MHz, DMSO-*d$_6$*) δ δ 11.21 (s, 1H, NH), 7.67 (d, J = 7.8 Hz, 2H), 7.45 (t, J = 7.7 Hz, 4H), 7.22 (t, J = 7.9 Hz, 5H), 7.11 (t, J = 7.6 Hz, 1H), 6.89 (t, J = 7.4 Hz, 1H), 6.67 (s, 1H), 4.83 (s, 1H), 4.55 (s, 1H), 3.11 (d, J = 14.3 Hz, 1H), 2.52 (s, 1H), 2.05 (s, 3H), 1.61 (s, 3H), 1.23 (s, 9H). **^{13}C NMR (126 MHz, DMSO-*d$_6$*)** δ 160.9 (C), 153.2, 145.2 (C), 142.9 (C), 136.5 (C), 136.0 (C), 133.9 (C), 128.9 (CH), 128.7 (CH), 128.1 (CH), 127.6 (CH), 127.1 (CH), 124.9 (CH), 123.7 (CH), 122.0 (C), 121.8 (CH), 119.H1, 108.6 (CH), 101.0 (C), 79.2 (C), 78.5 (C), 60.2 (C), 43.1 (CH$_2$), 41.9 (CH$_2$), 28.0 (CH$_3$), 27.1 (CH$_3$), 11.9 (CH$_3$). **HRMS (ESI/Q-TOF)** m/z: $[M+H]^+$ $C_{34}H_{36}N_5O_4^+$ Calcd for 578.2762; Found 578.2749.

CHARACTERITZATION OF THE PRODUCT 6 (transformation iii)

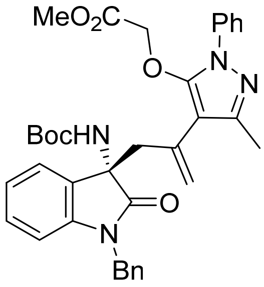

6

Following the General Procedure **vi**, product **6** was obtained as a white solid (40.6 mg, 89% yield) using as eluent mixtures of hexane/Et_2O 60:40 to hexane/Et_2O 50:50. Enantiomeric excess (98%) was determined by chiral HPLC (Chiralcel® ODH *i*PrOH/hexane = 10/90, flow rate= 1.0 mL/min, λ = 254 nm) t_R = 17.16 min (major), 24.15 min (minor). $[\alpha_D^{20}]$= +16.6 (c 0.6, $CHCl_3$). m.p.= 200-201 ºC.

^{1}H NMR (300 MHz, $CDCl_3$) δ 7.50 (d, *J* = 7.5 Hz, 2H), 7.41 – 7.30 (m, 4H), 7.26 – 7.15 (m, 4H), 7.05 – 6.89 (m, 2H), 6.73 (td, *J* = 7.6, 0.8 Hz, 1H), 6.54 (d, *J* = 7.9 Hz, 1H), 5.22 (s, 1H, NH), 5.14 (s, 1H), 4.91 (d, *J* = 1.5 Hz, 1H), 4.71 (s, 1H), 4.67 (s, 1H), 4.22 (s, 2H), 3.56 (s, 3H), 3.14 (d, *J* = 12.6 Hz, 1H), 3.08 (d, *J* = 12.8 Hz, 1H), 1.79 (s, 3H), 1.19 (s, 9H). **^{13}C NMR (75 MHz, $CDCl_3$)** δ 176.6 (C), 167.9 (C), 153.6 (C), 148.5 (C), 146.5 (C), 142.5 (C), 138.0 (C), 135.8 (C), 132.3 (C), 129.1 (CH), 128.6 (CH), 127.7 (CH), 127.4 (CH), 127.0 (CH), 125.5 (C), 123.3 (CH), 122.89 (CH), 122.86 (CH), 122.1 (CH), 120.7 (CH2), 109.0 (CH), 107.5 (C), 80.2 (C), 70.0 (CH_2), 61.8 (C), 52.1 (CH_3), 44.0 (CH_2), 42.8 (CH_2), 28.1 (CH_3), 13.4 (CH_3). **HRMS (ESI/Q-TOF)** m/z: $[M+H]^+$ $C_{36}H_{39}N_4O_6^+$ Calcd for 623.2864; Found 623.2851.

NMR SPECTRA (^{1}H-NMR and ^{13}C-NMR)

a. ALKYLIDENE PYRAZOLONES **2** SPECTRA

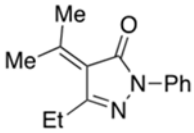

2b, ^{1}H NMR (300 MHz, CDCl$_3$)
^{13}C NMR (75 MHz, CDCl$_3$)

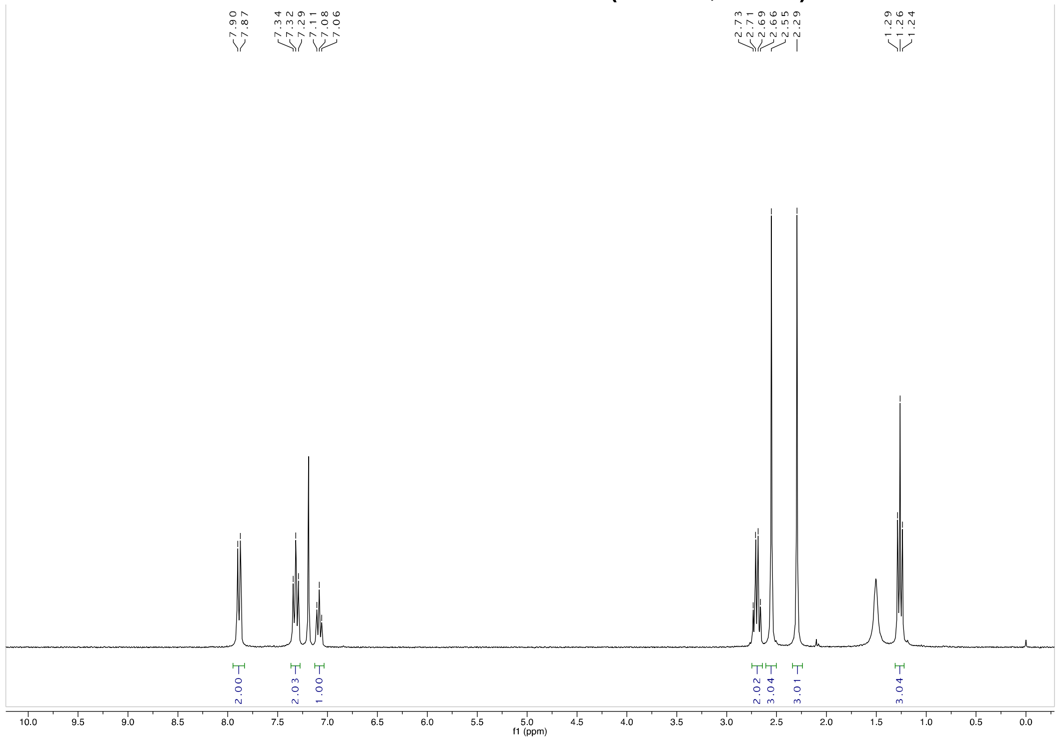

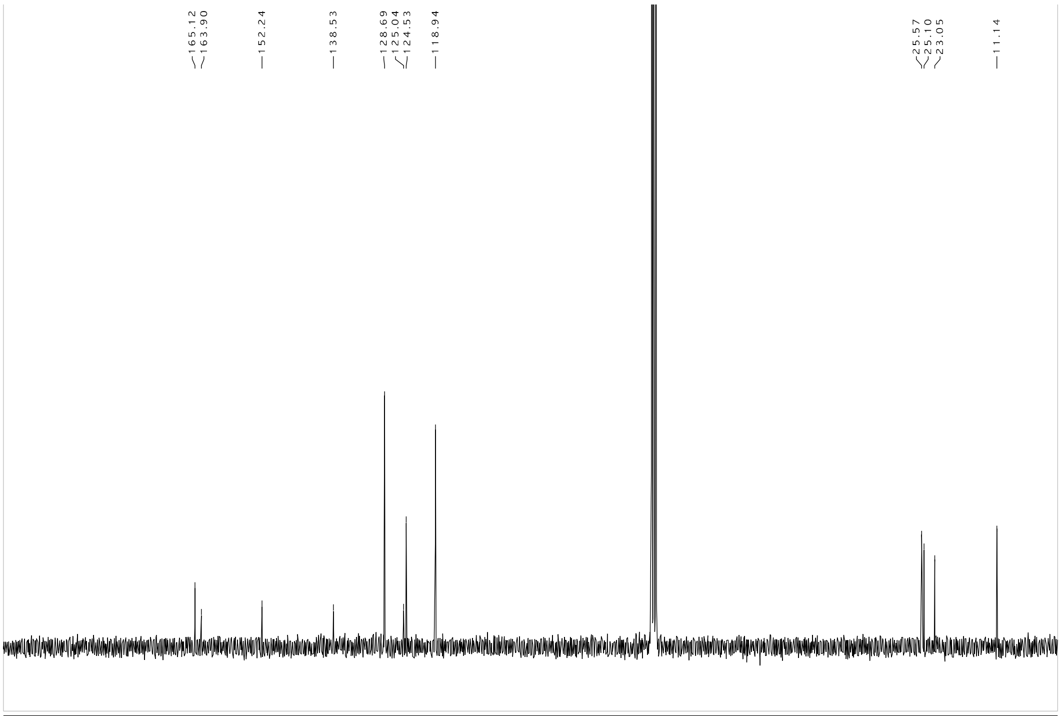

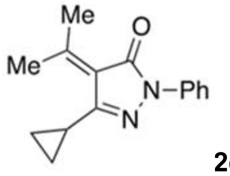

2d, ^{1}H NMR (300 MHz, $CDCl_3$)
^{13}C NMR (75 MHz, $CDCl_3$)

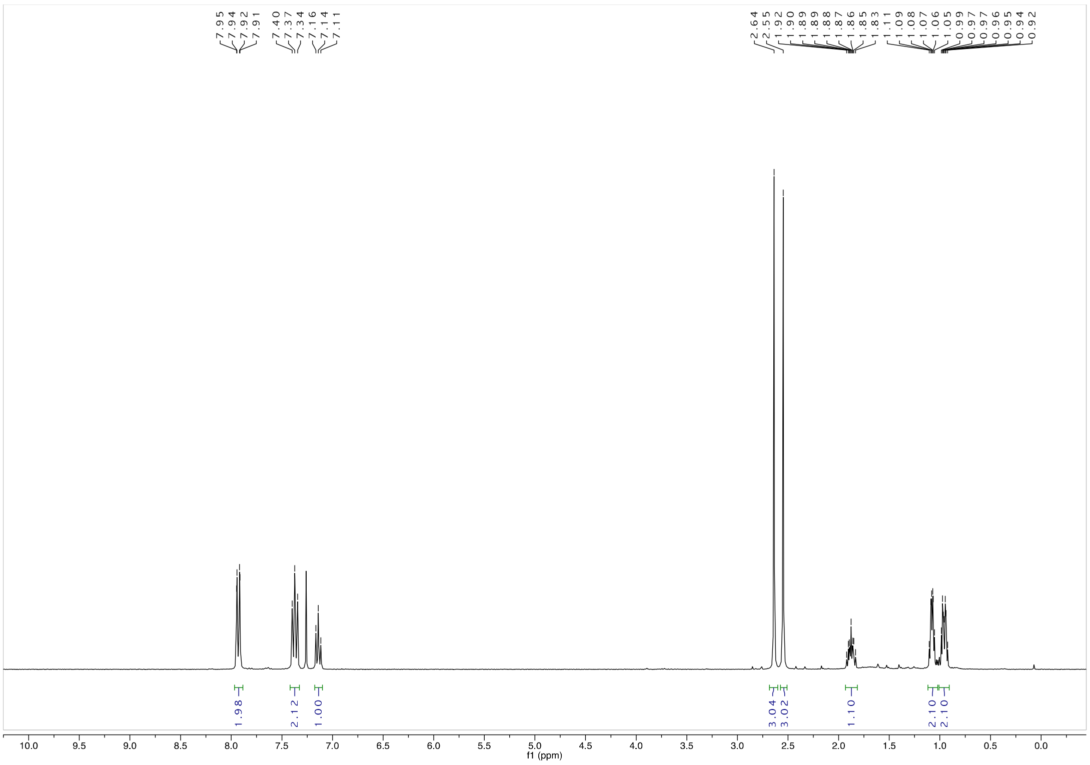

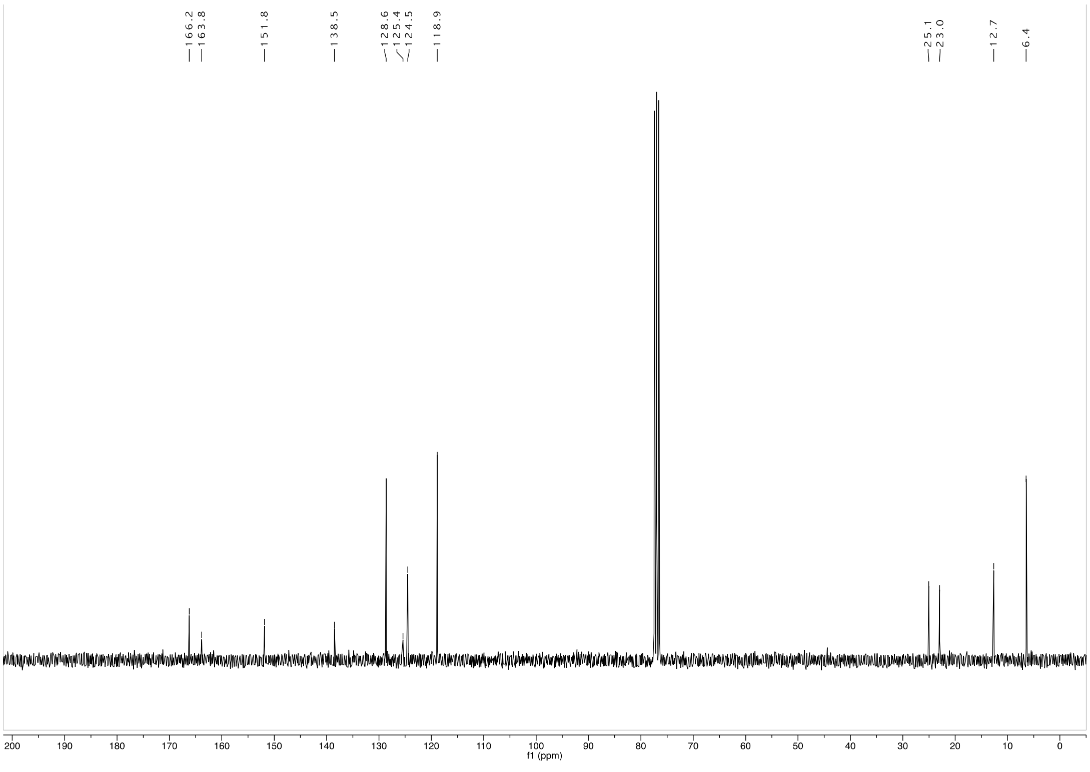

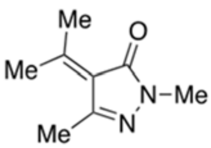

2f, ^{1}H NMR (300 MHz, $CDCl_3$)
^{13}C NMR (75 MHz, $CDCl_3$)

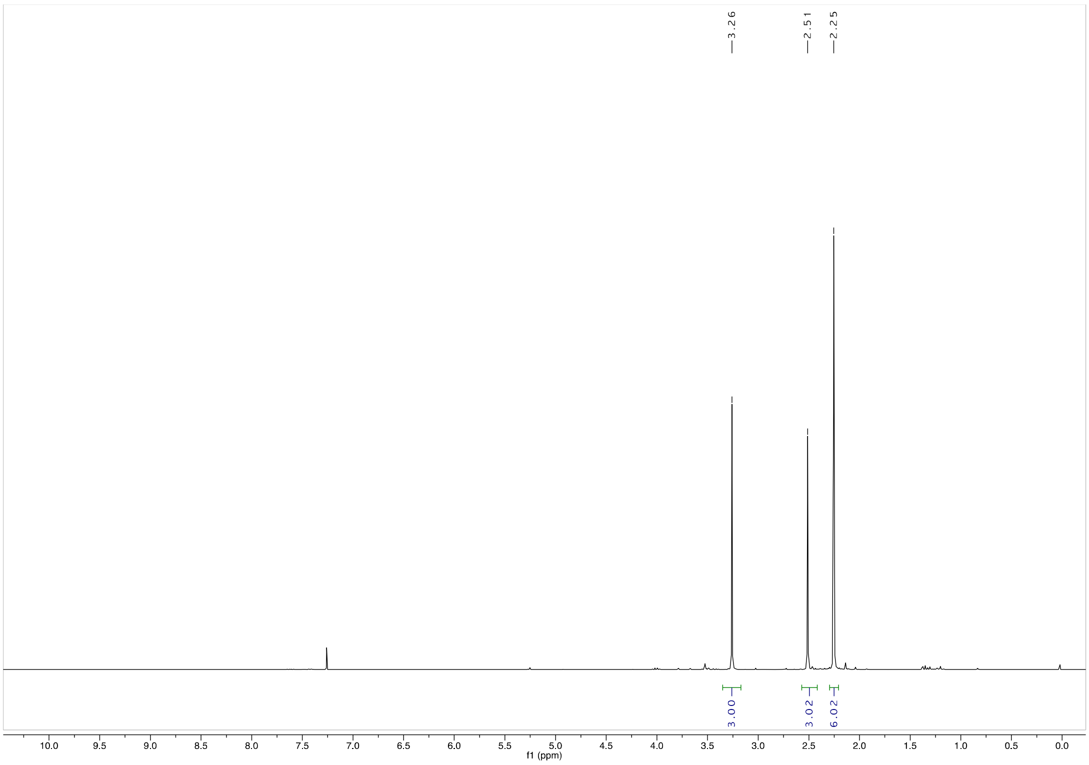

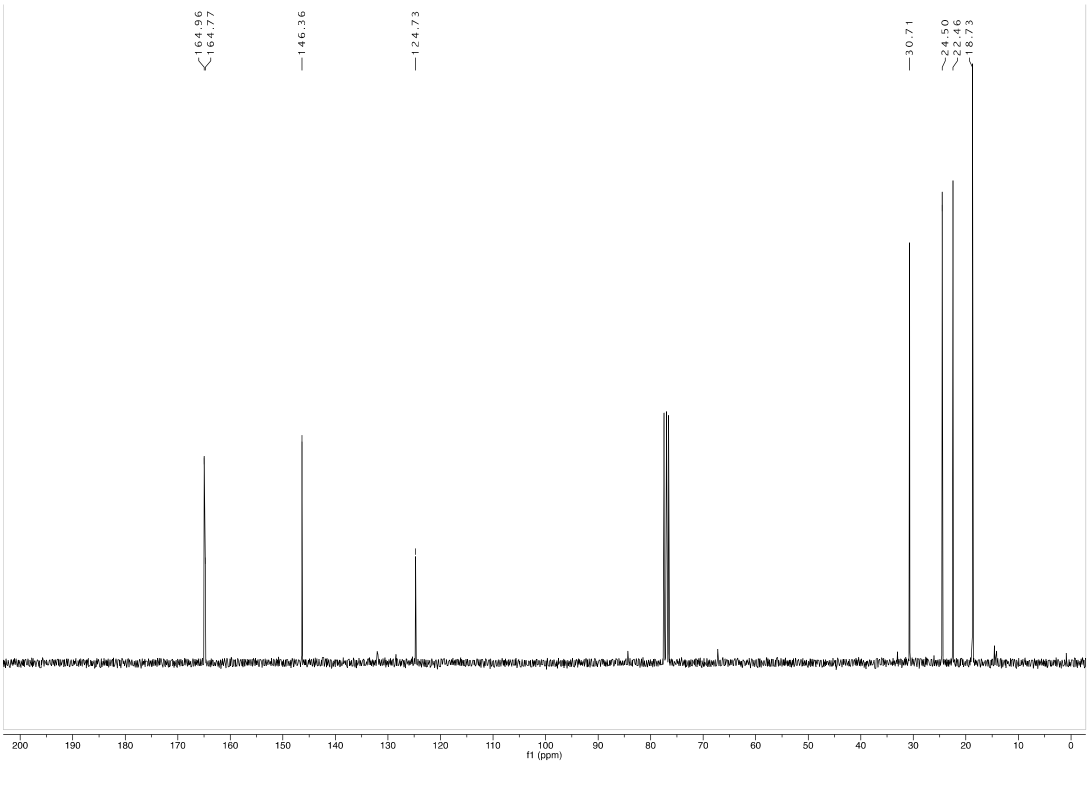

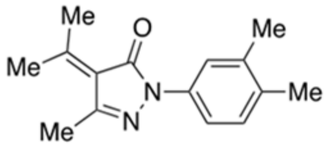

3k, ^{1}H NMR (300 MHz, $CDCl_3$)
^{13}C NMR (75 MHz, $CDCl_3$)

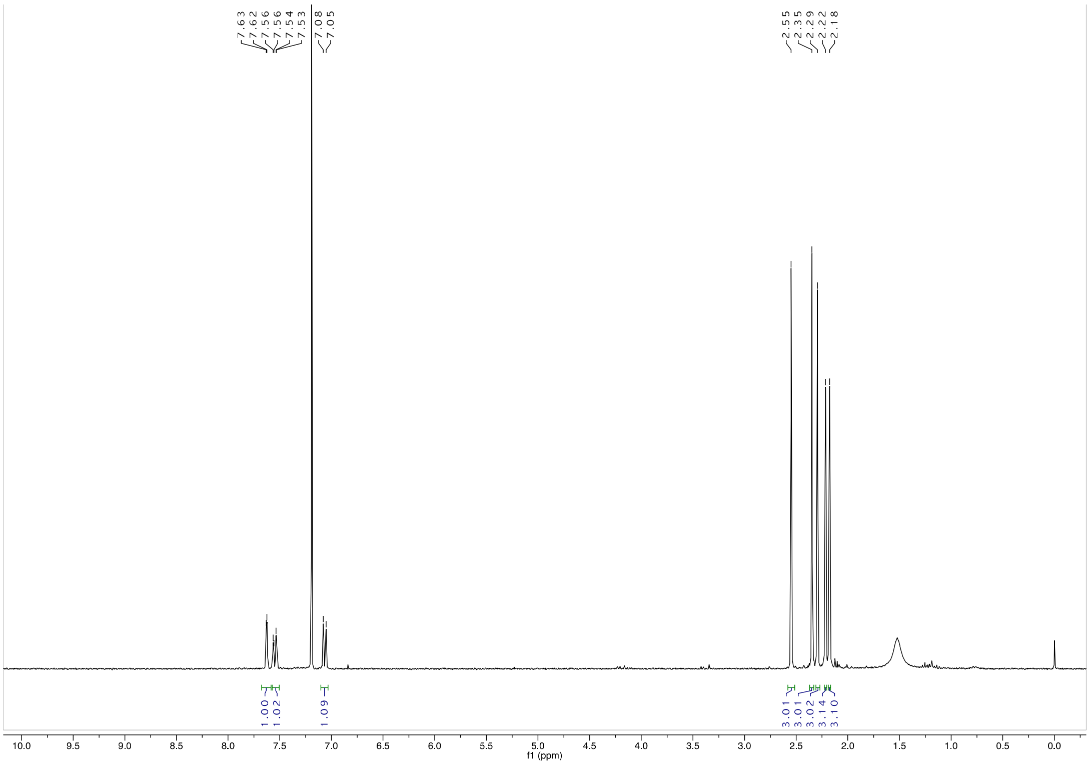

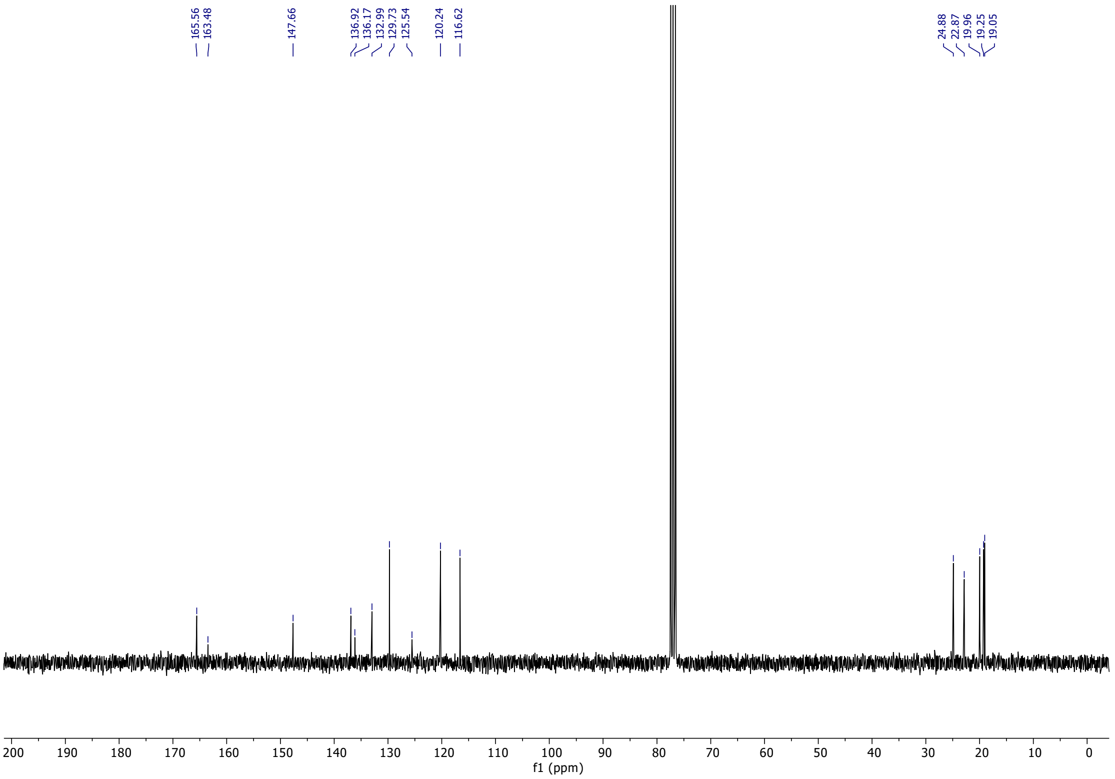

b. FINAL AMINES **3** SPECTRA

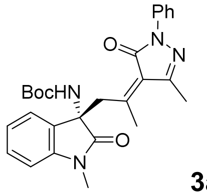

3aa, ¹H NMR (300 MHz, CDCl₃)
¹³C NMR (75 MHz, CDCl₃)

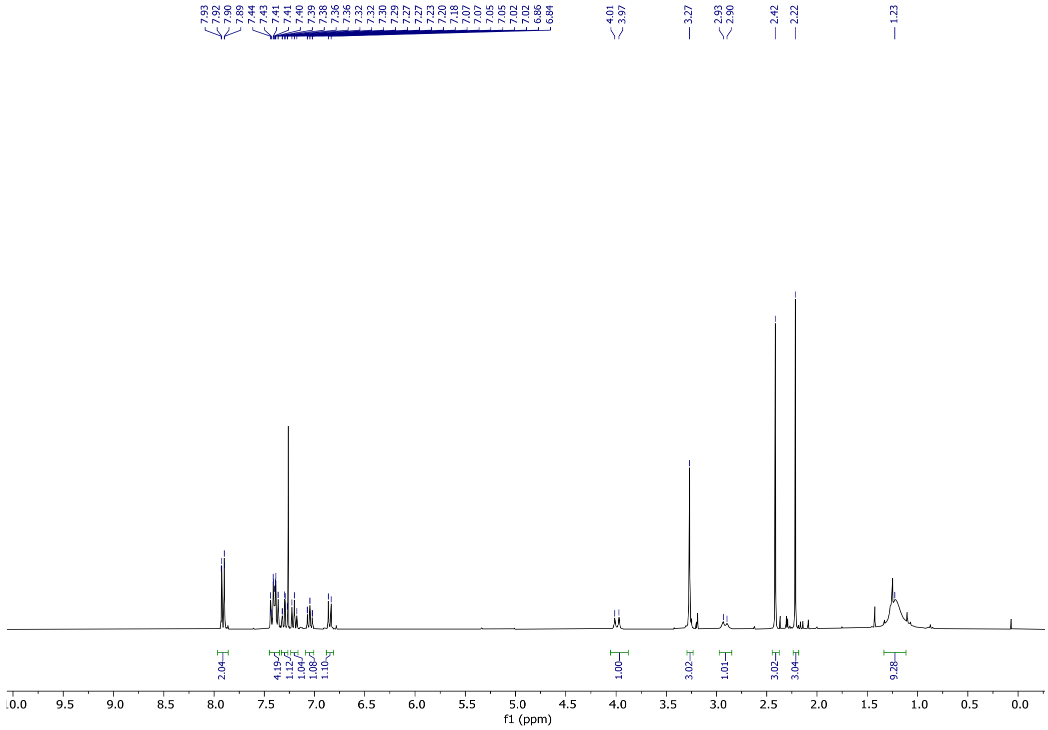

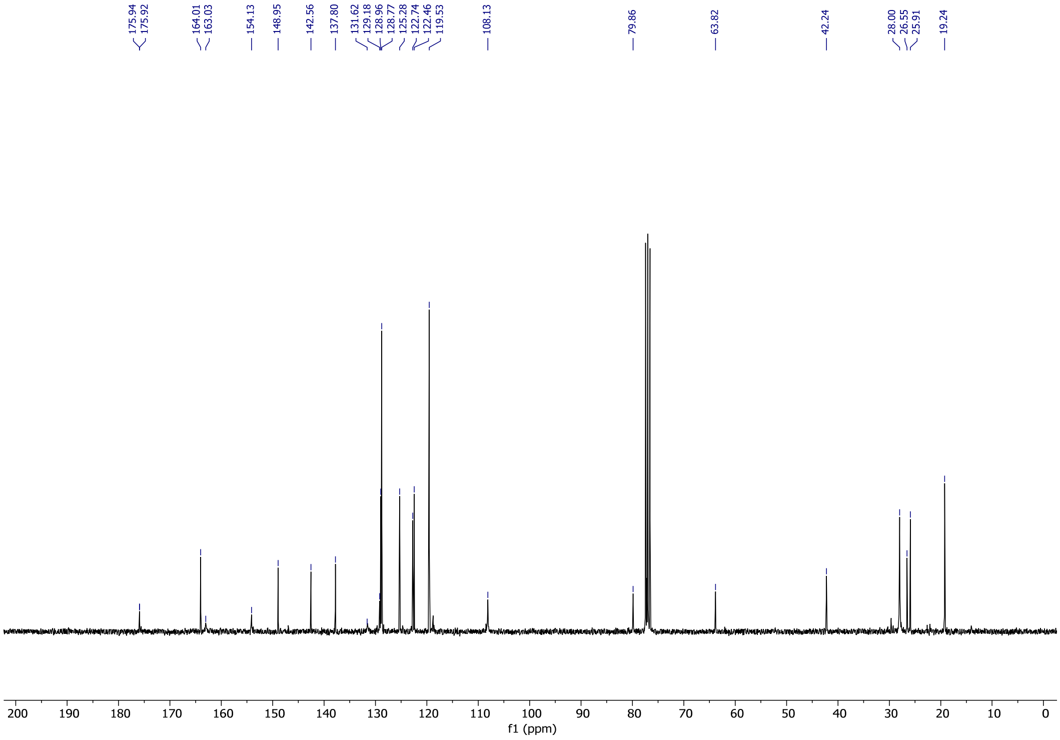

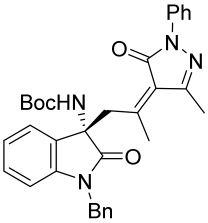

3ba, ^{1}H NMR (300 MHz, $CDCl_3$)
^{13}C NMR (75 MHz, $CDCl_3$)

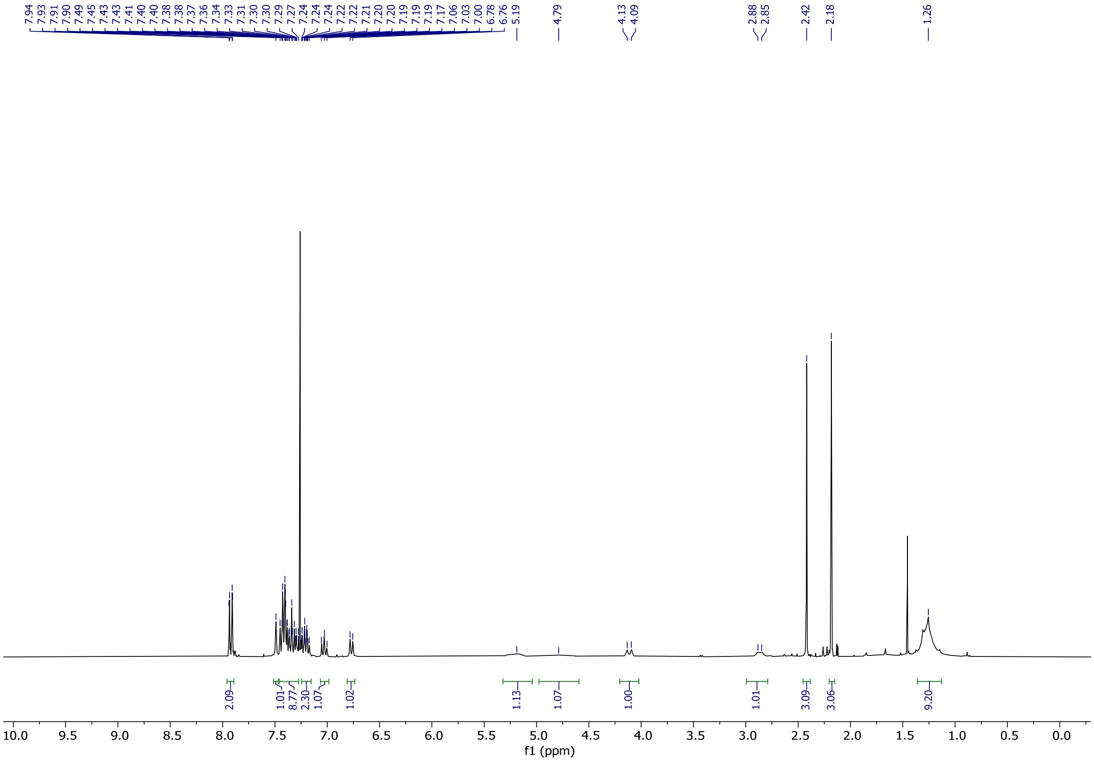

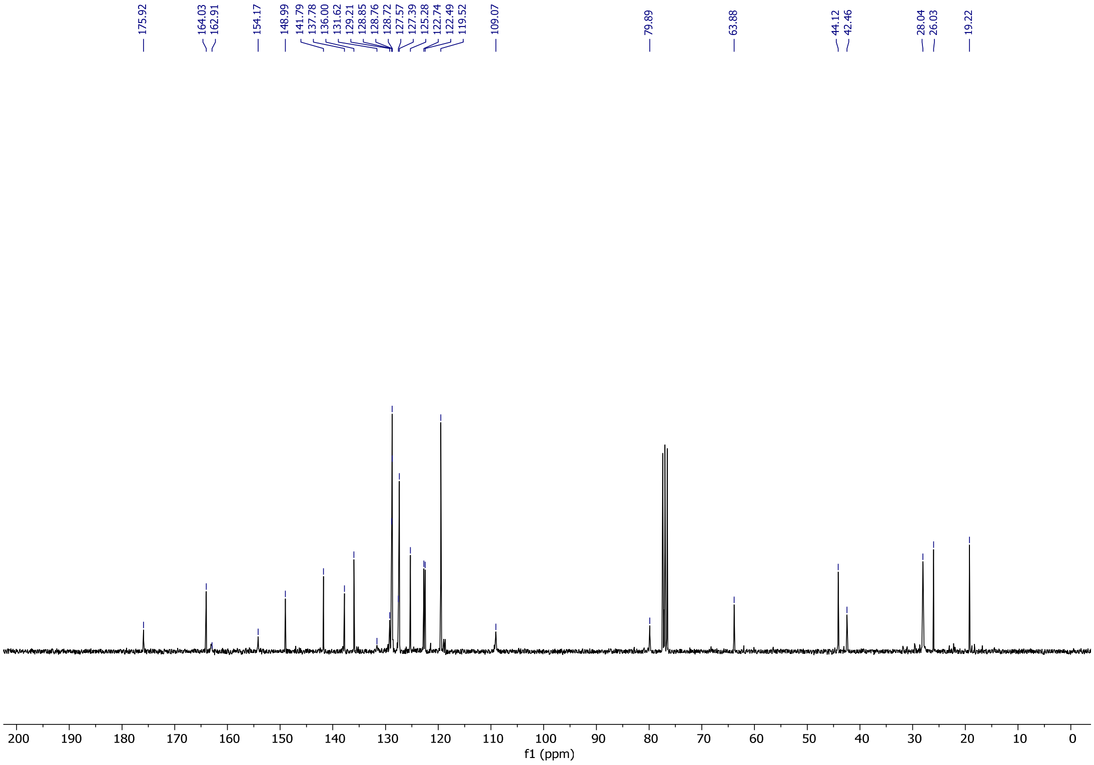

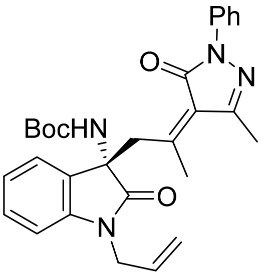

3ca, ^{1}H NMR (300 MHz, $CDCl_3$)
^{13}C NMR (75 MHz, $CDCl_3$)

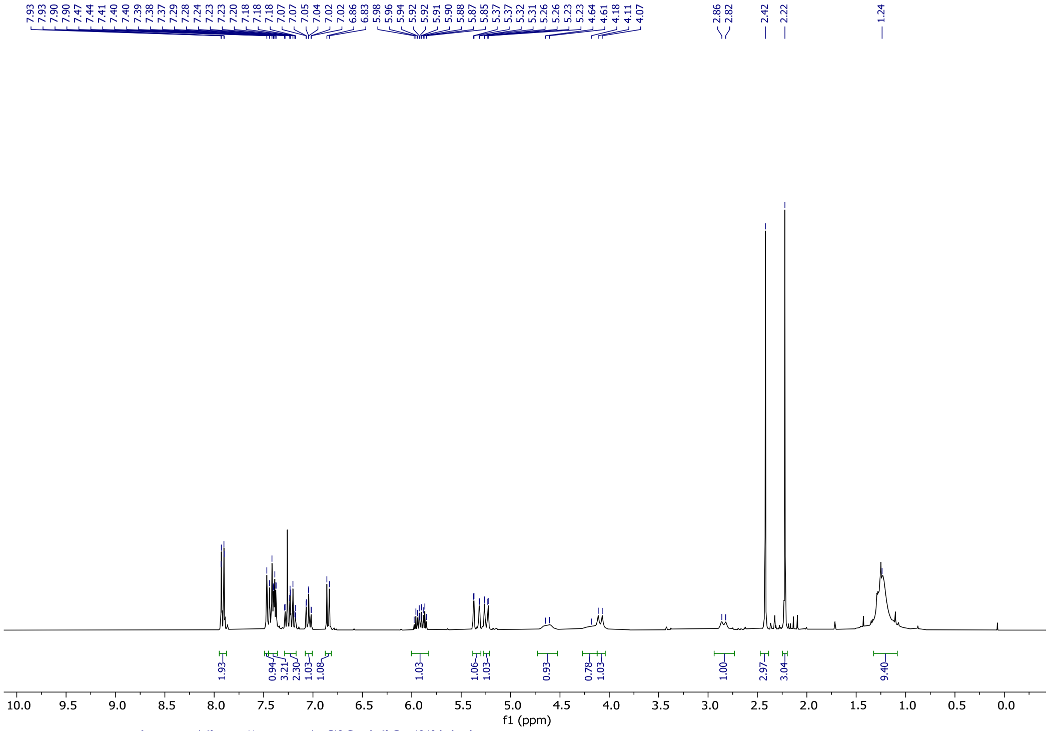

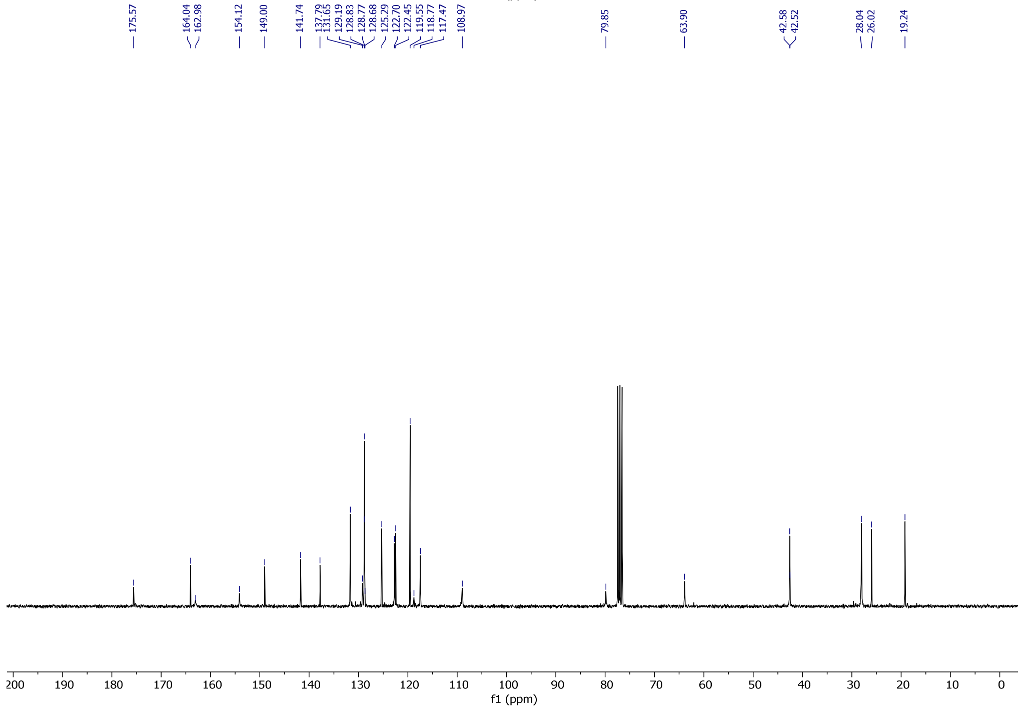

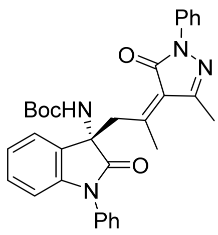

3da, ^{1}H NMR (300 MHz, CDCl$_3$)
^{13}C NMR (75 MHz, CDCl$_3$)

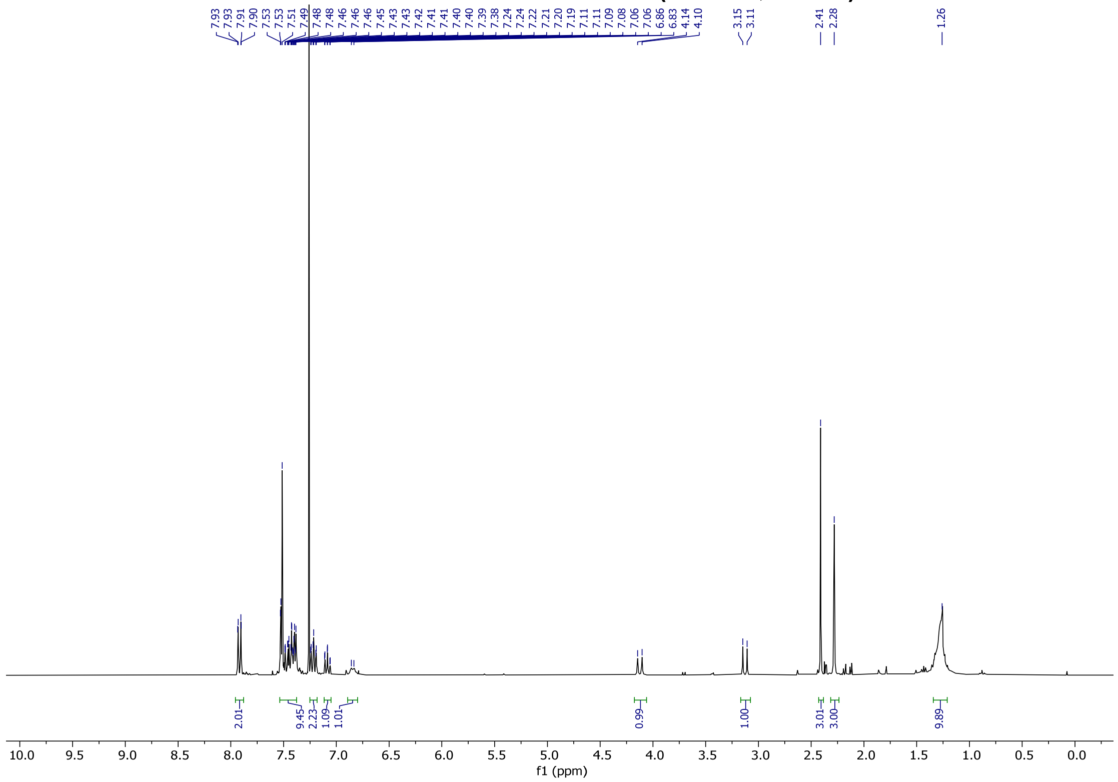

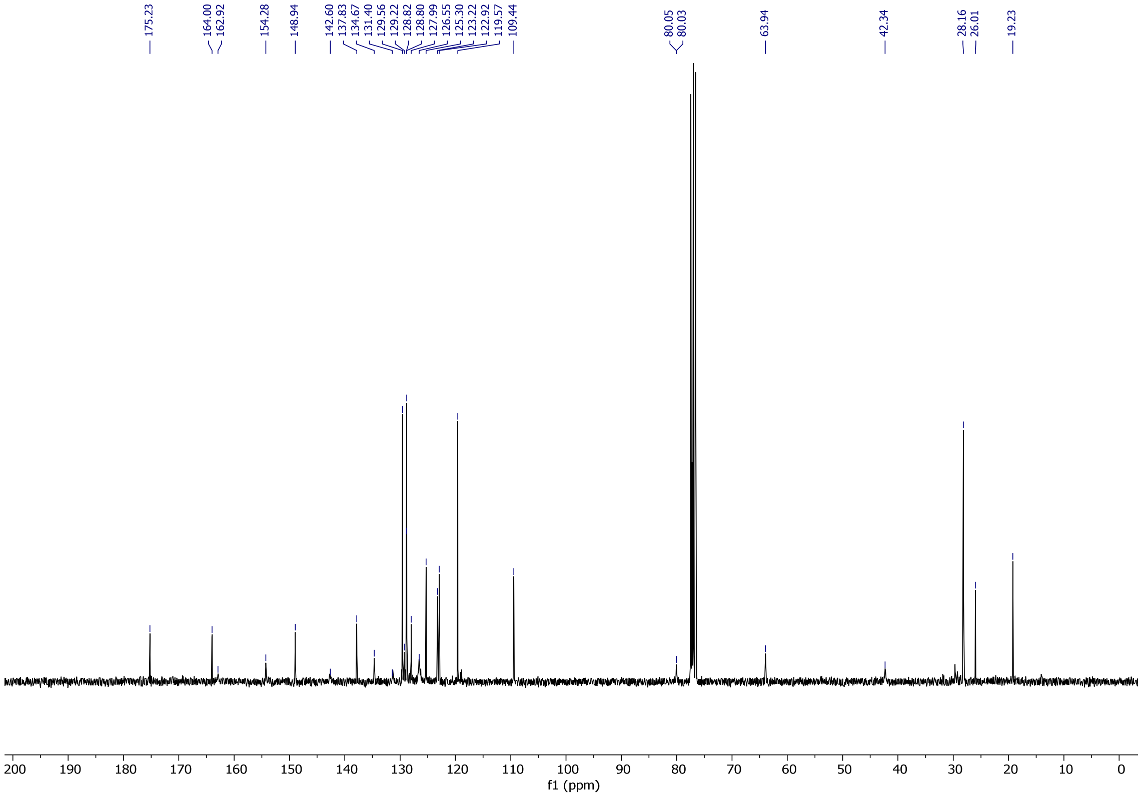

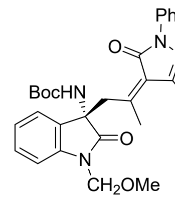

3ea, ^{1}H NMR (300 MHz, $CDCl_3$)
^{13}C NMR (75 MHz, $CDCl_3$)

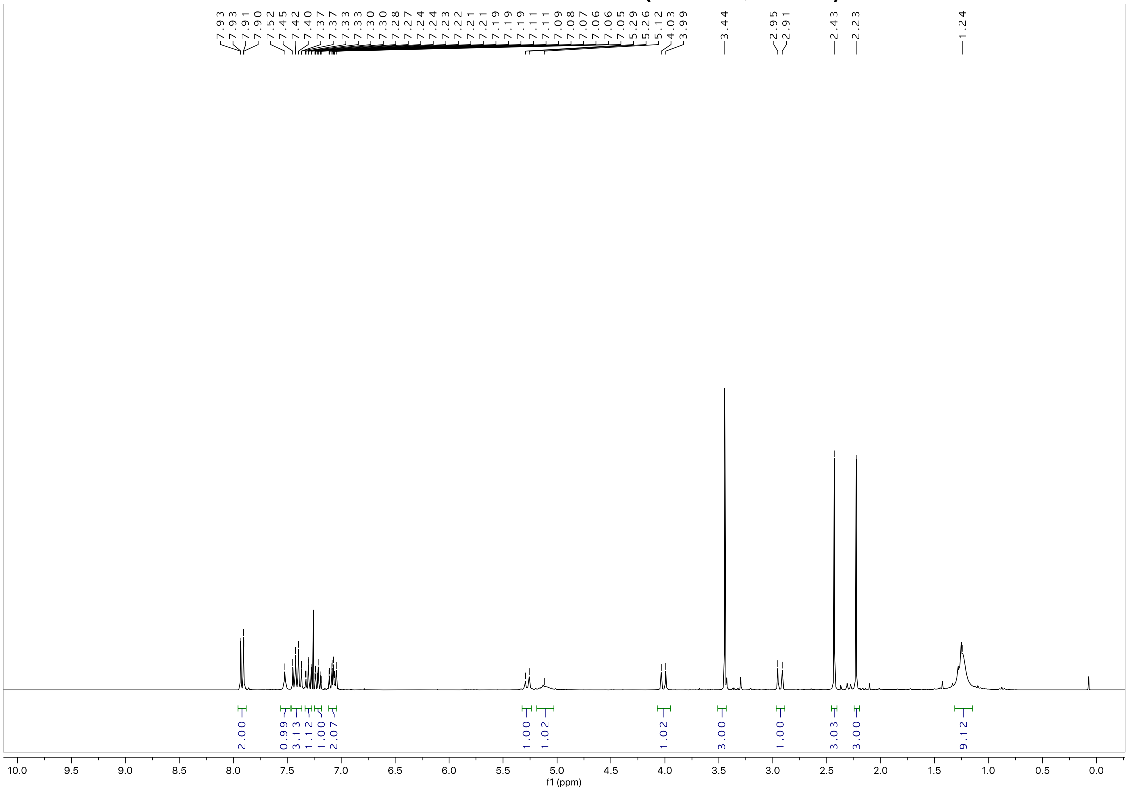

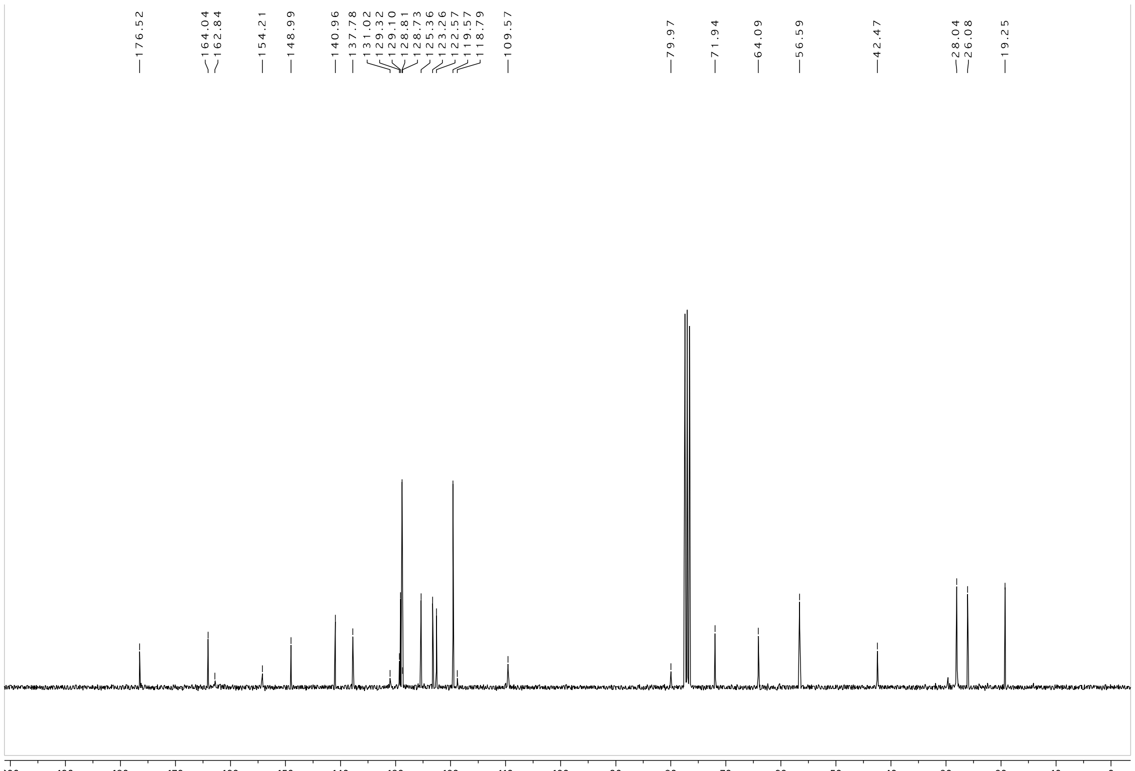

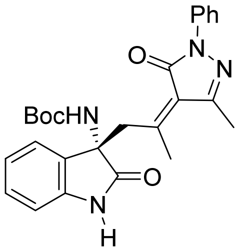

3fa, ^{1}H NMR (300 MHz, $CDCl_3$)
^{13}C NMR (75 MHz, $CDCl_3$)

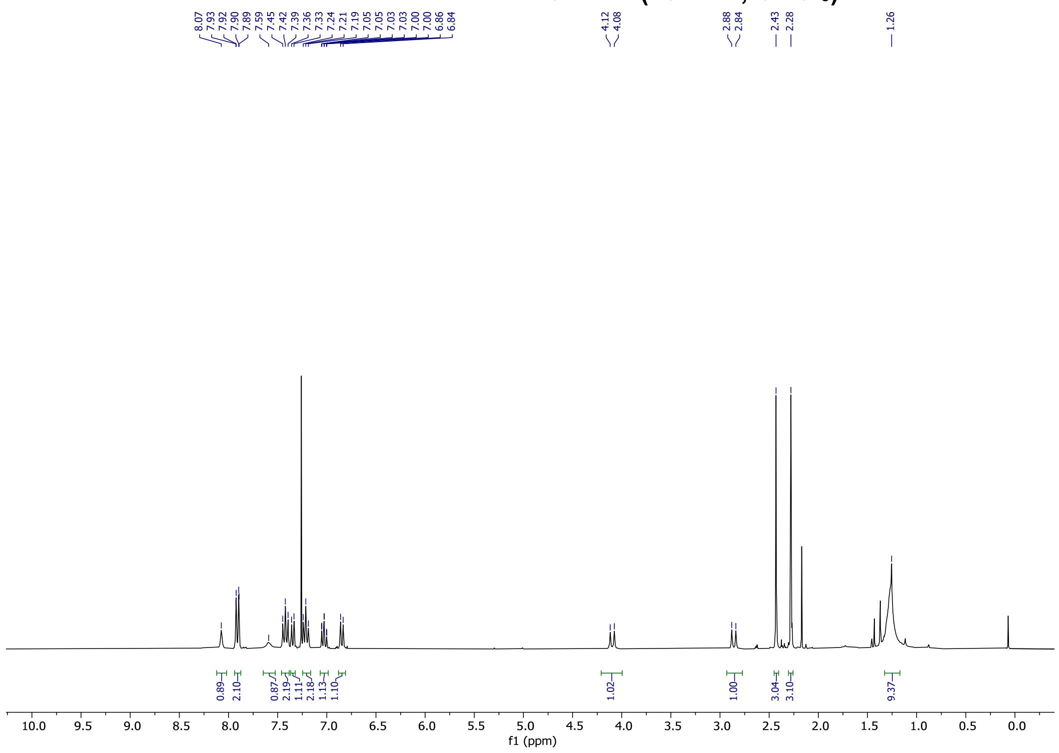

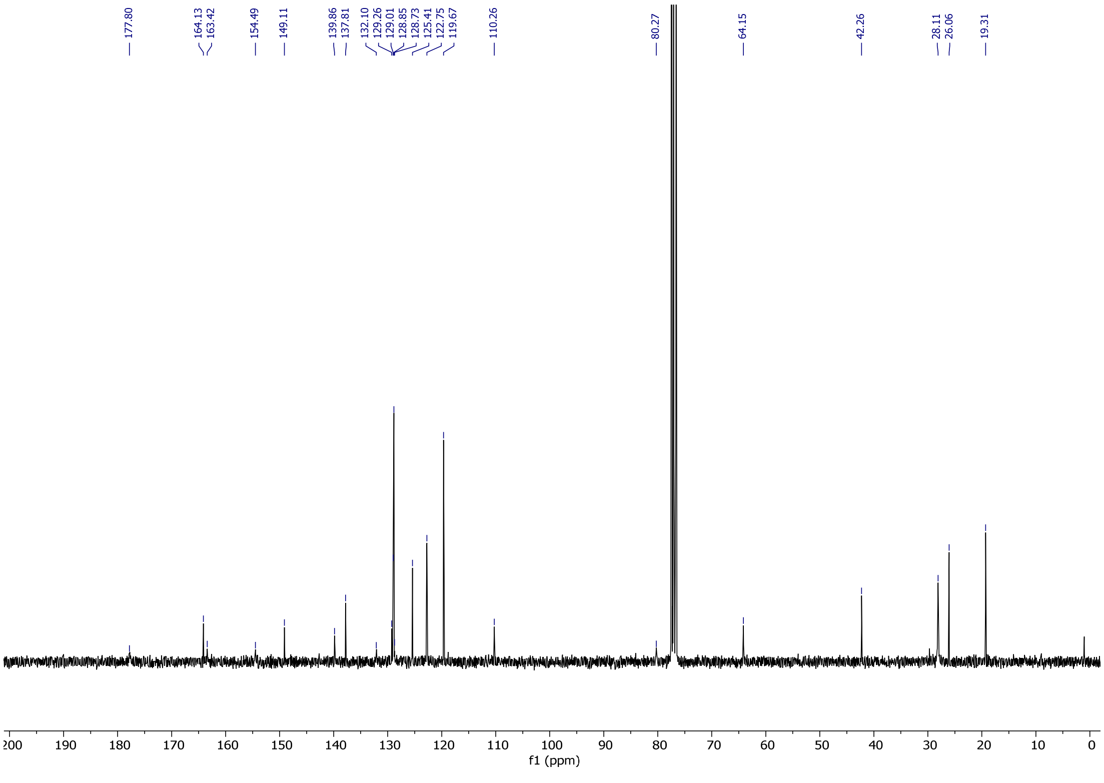

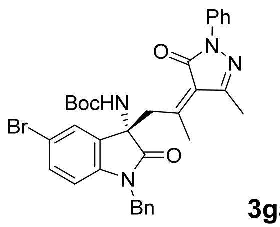

3ga, ^{1}H NMR (300 MHz, $CDCl_3$)
^{13}C NMR (75 MHz, $CDCl_3$)

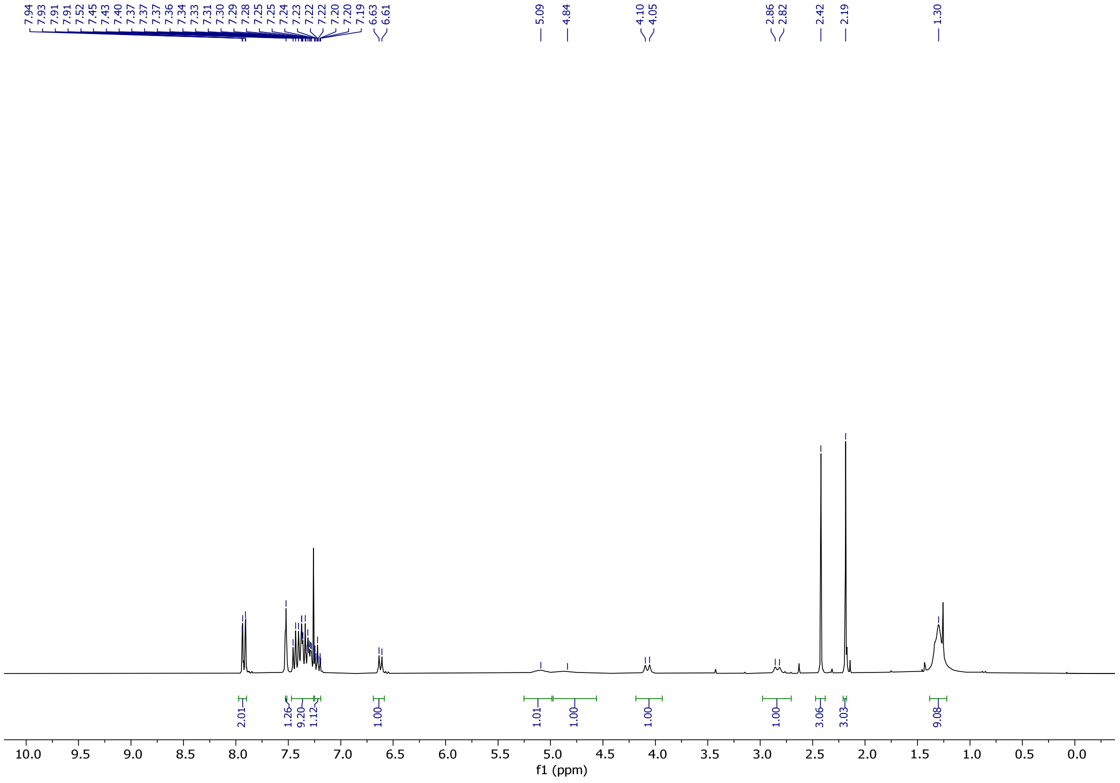

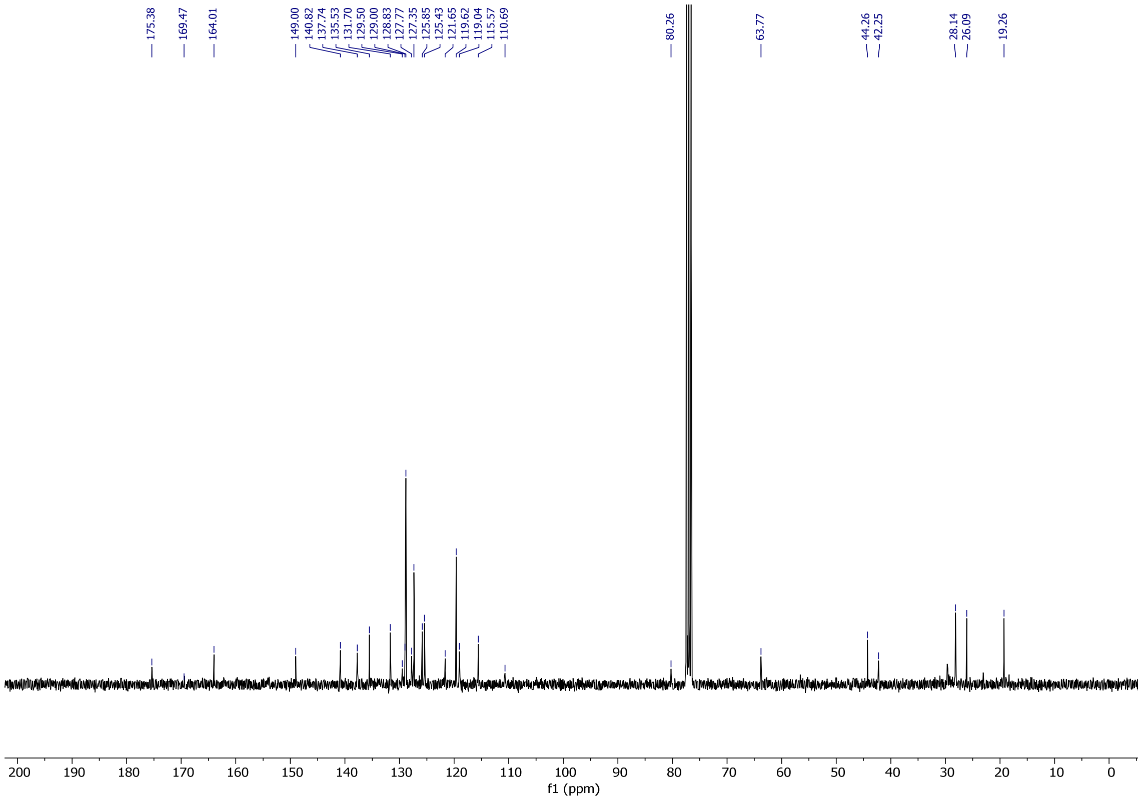

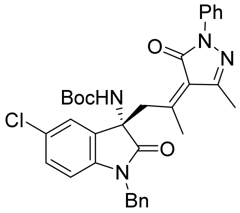

3ha, ^{1}H NMR (300 MHz, $CDCl_3$)
^{13}C NMR (75 MHz, $CDCl_3$)

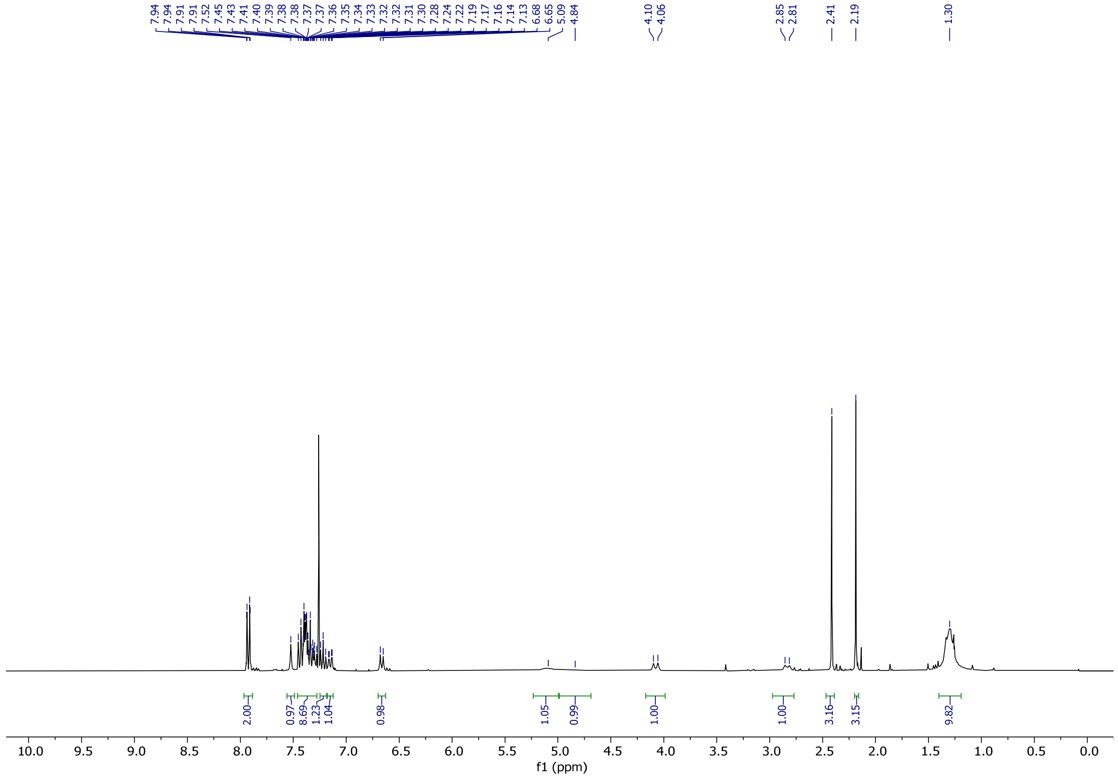

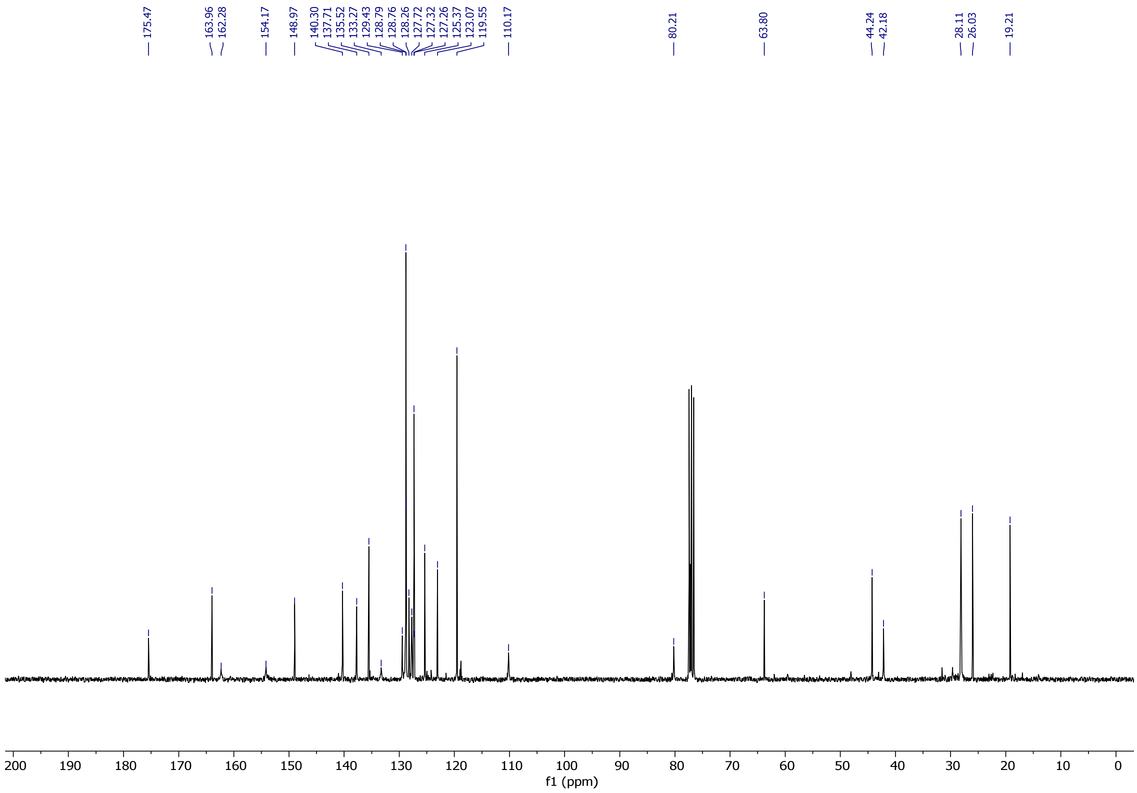

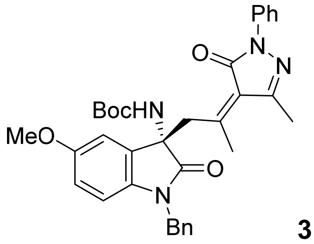

3ia, ^{1}H NMR (300 MHz, $CDCl_3$)
^{13}C NMR (75 MHz, $CDCl_3$)

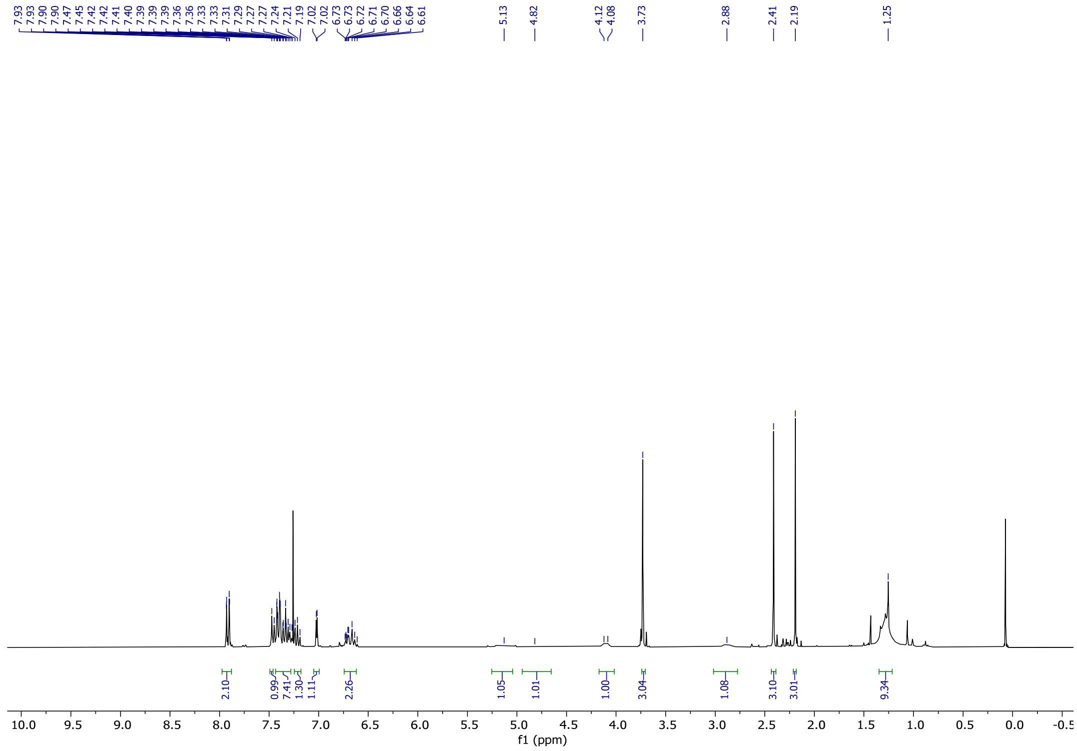

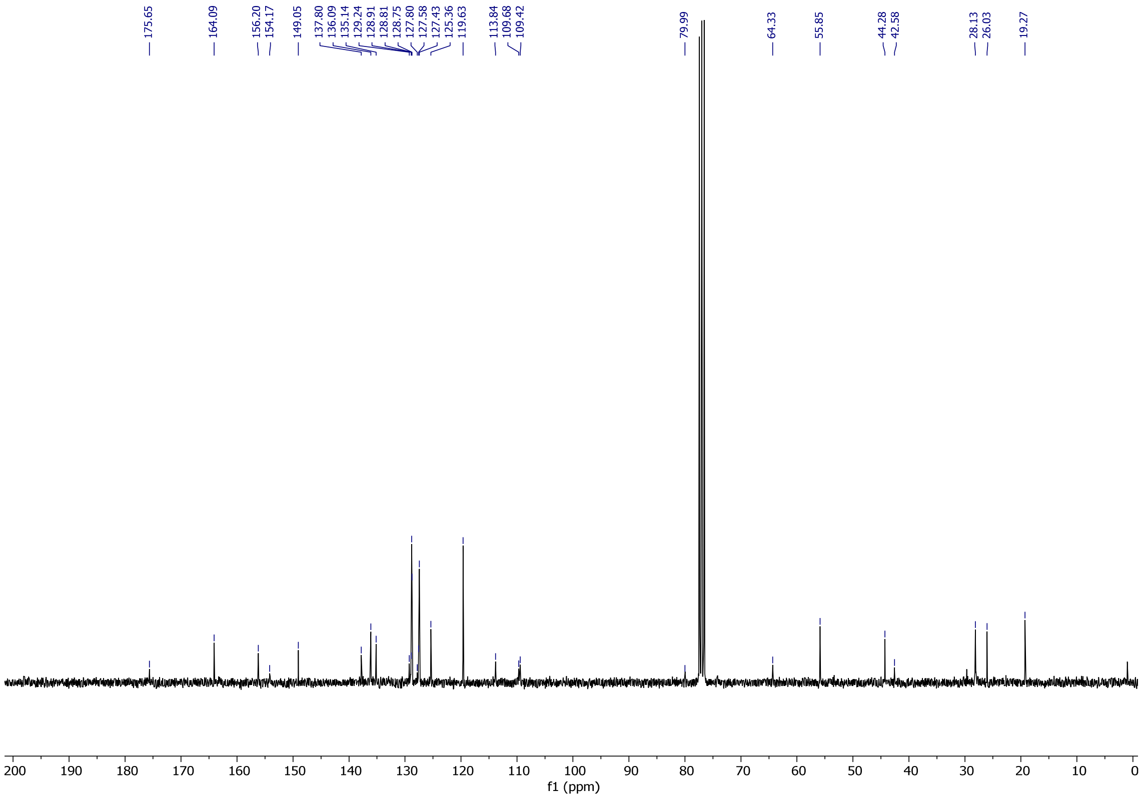

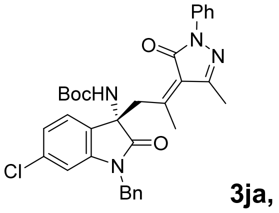

3ja, ^{1}H NMR (300 MHz, $CDCl_3$)
^{13}C NMR (75 MHz, $CDCl_3$)

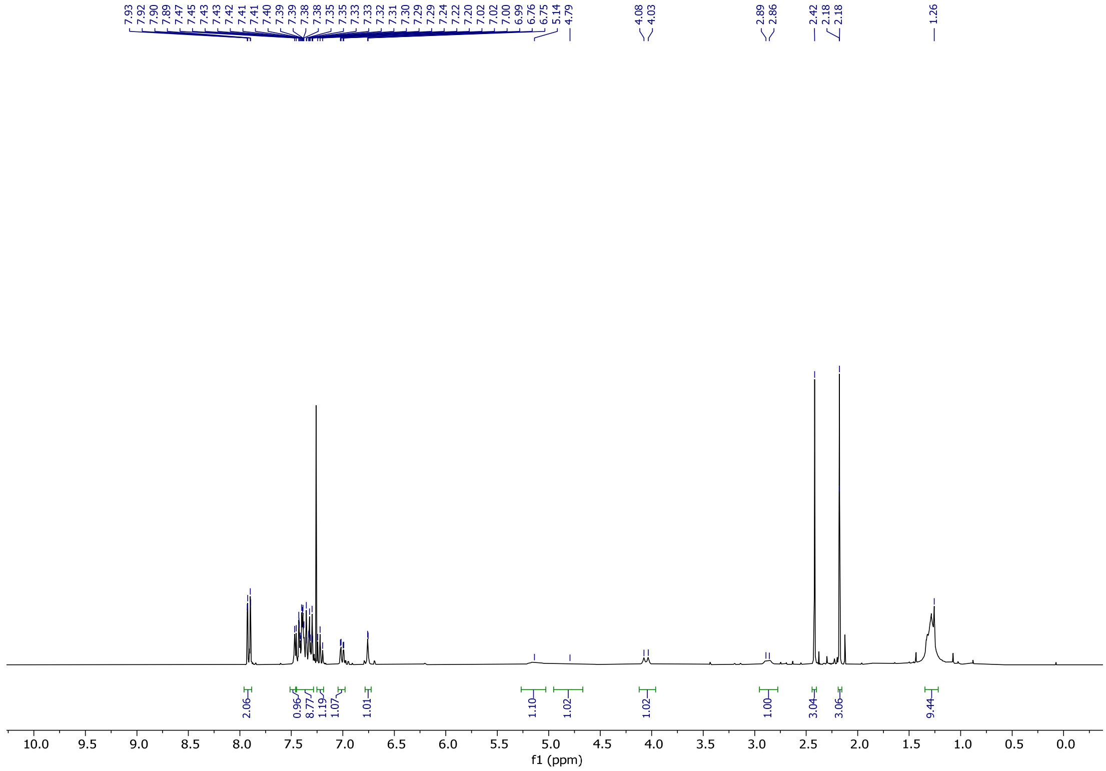

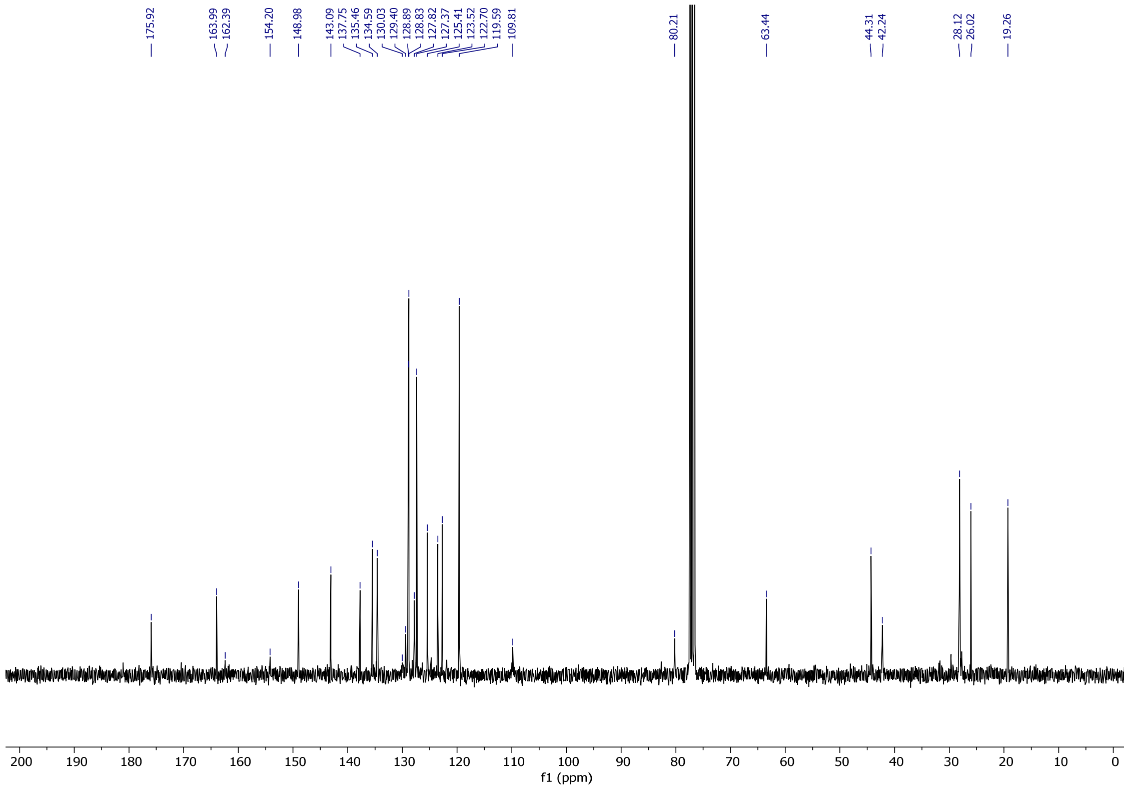

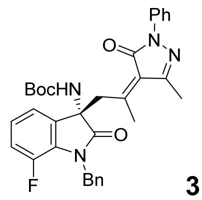

3ka, ^{1}H NMR (300 MHz, $CDCl_3$)
^{13}C NMR (75 MHz, $CDCl_3$)

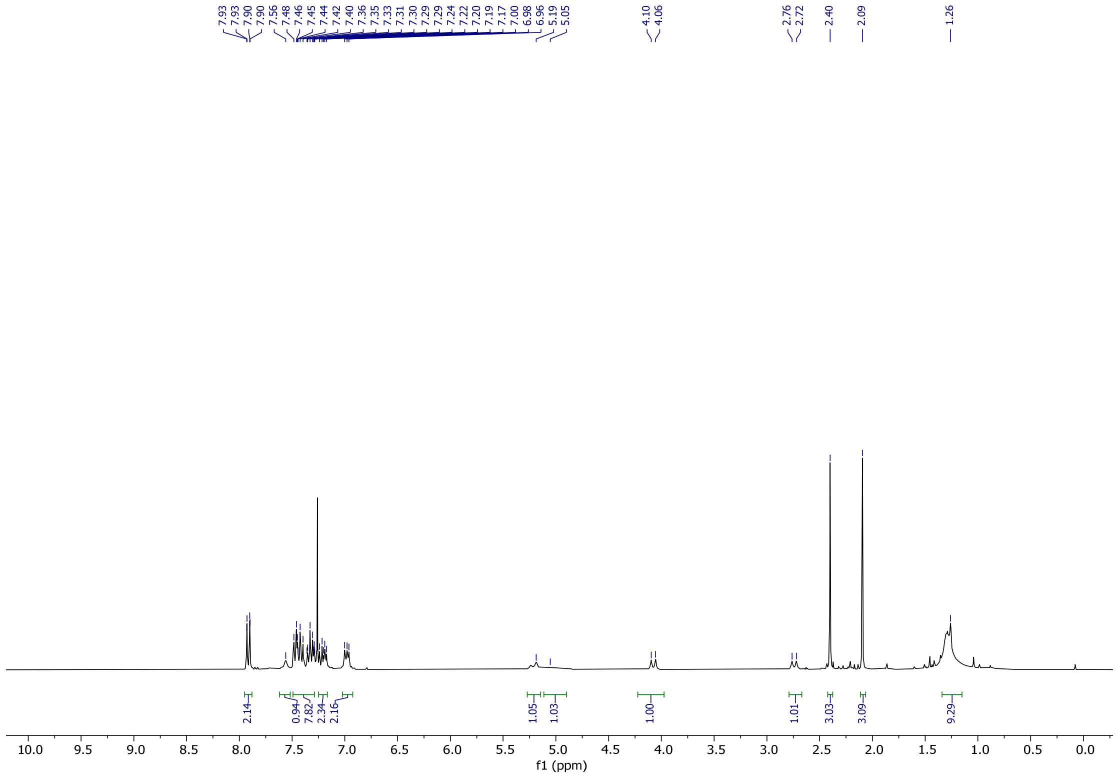

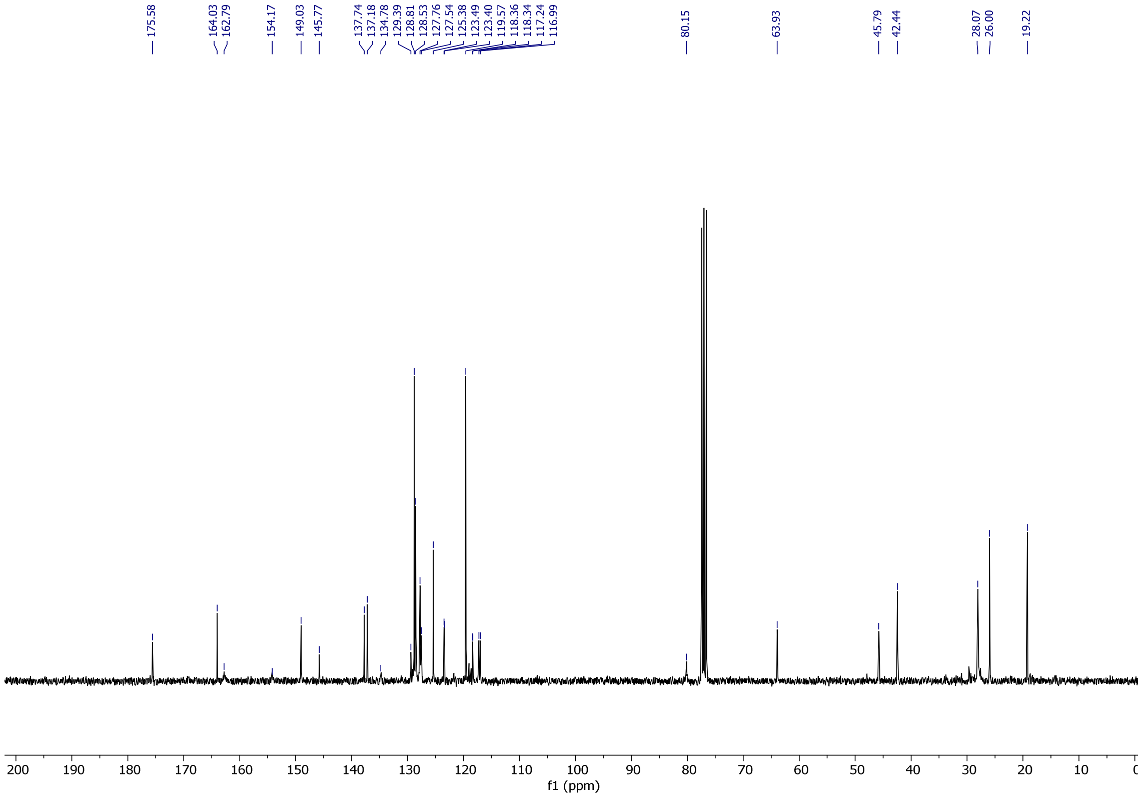

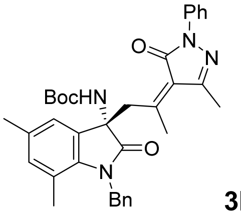

3la, ¹H NMR (300 MHz, CDCl₃)
¹³C NMR (75 MHz, CDCl₃)

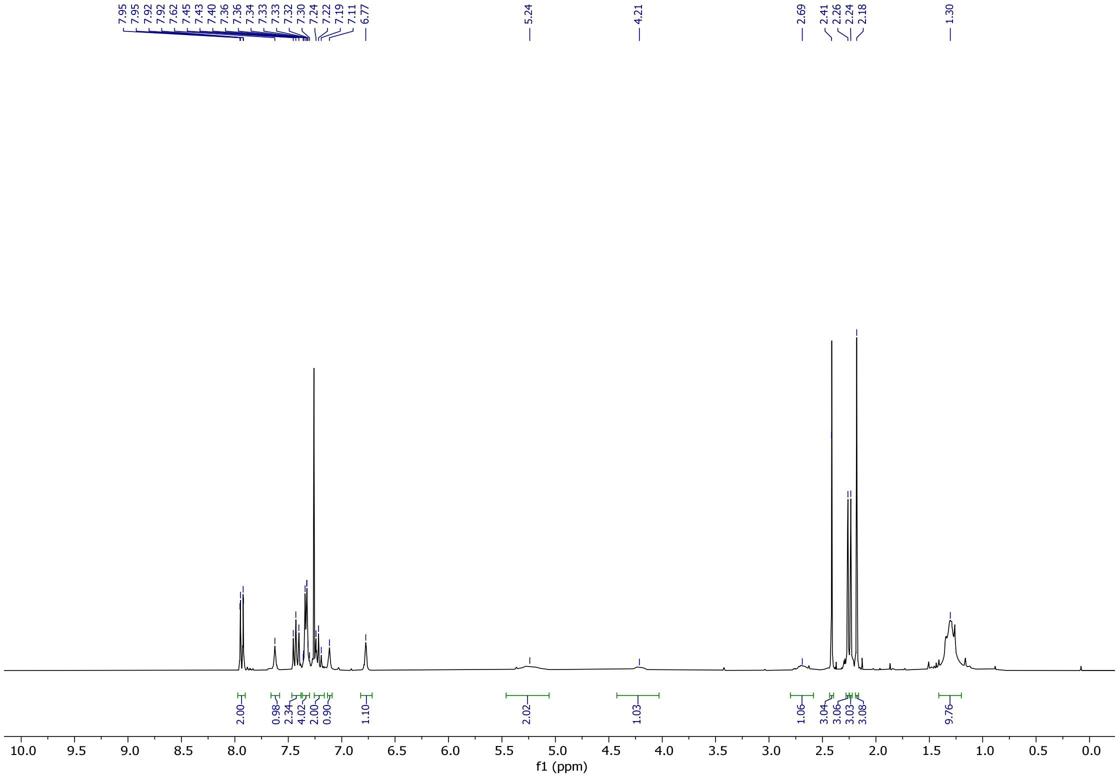

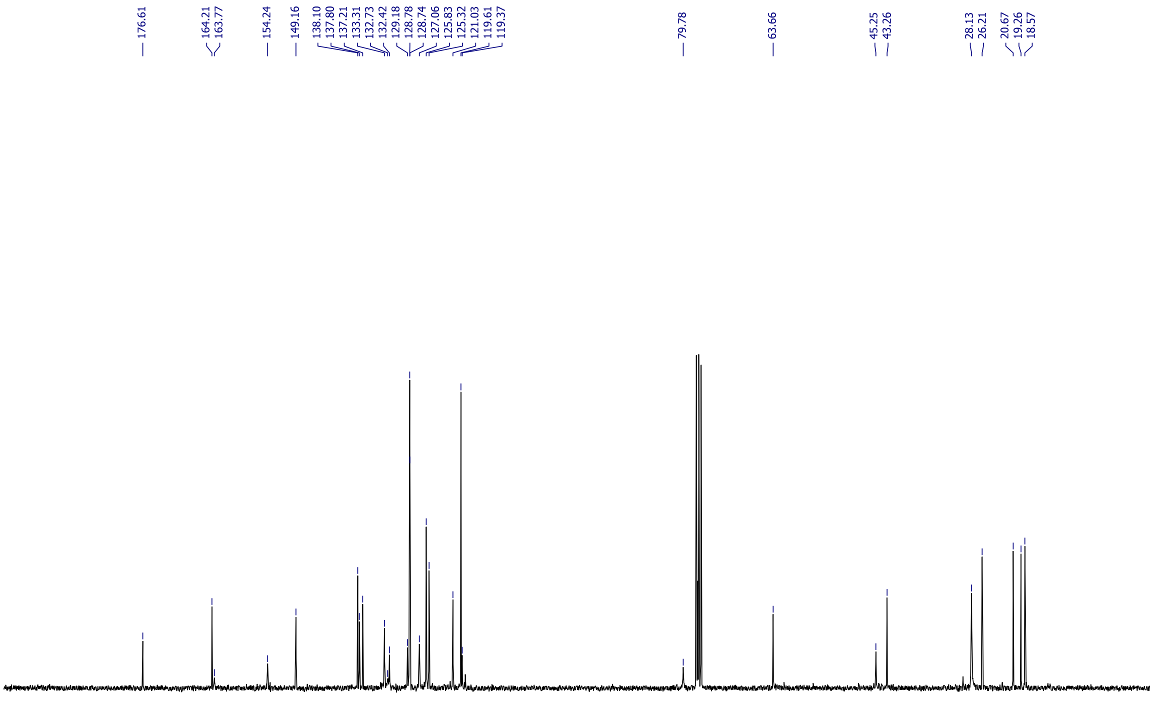

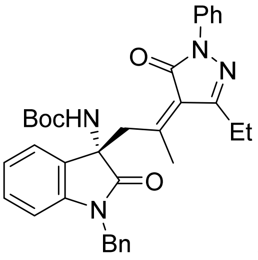

3bb, ^1H NMR (300 MHz, $CDCl_3$)
^{13}C NMR (75 MHz, $CDCl_3$)

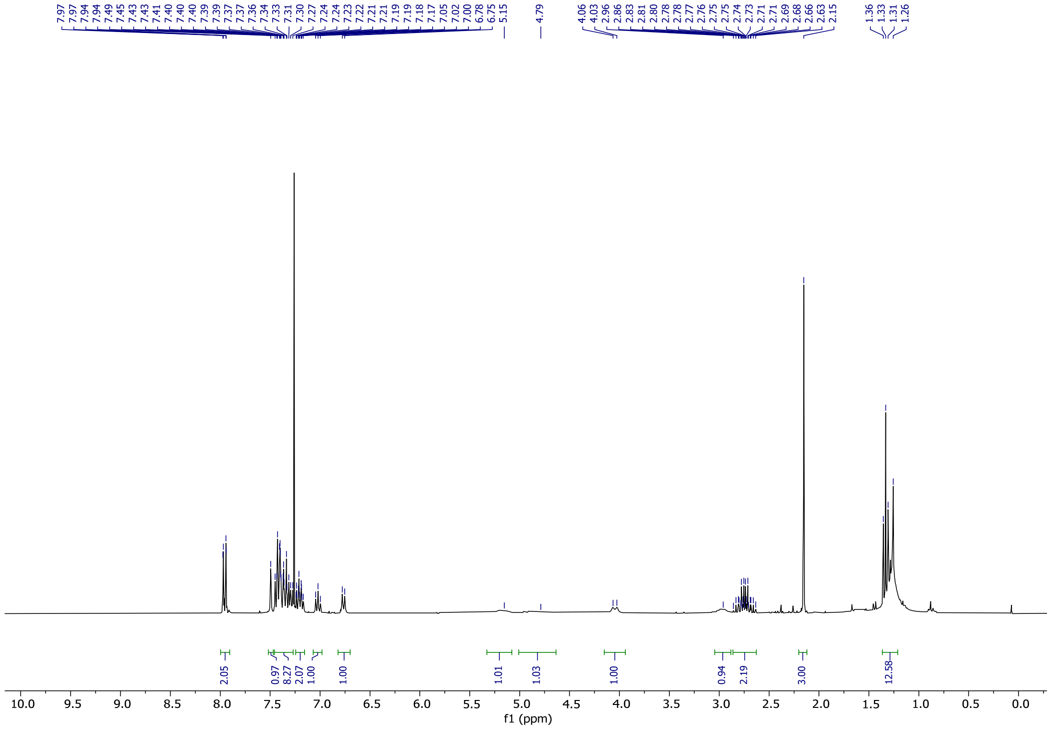

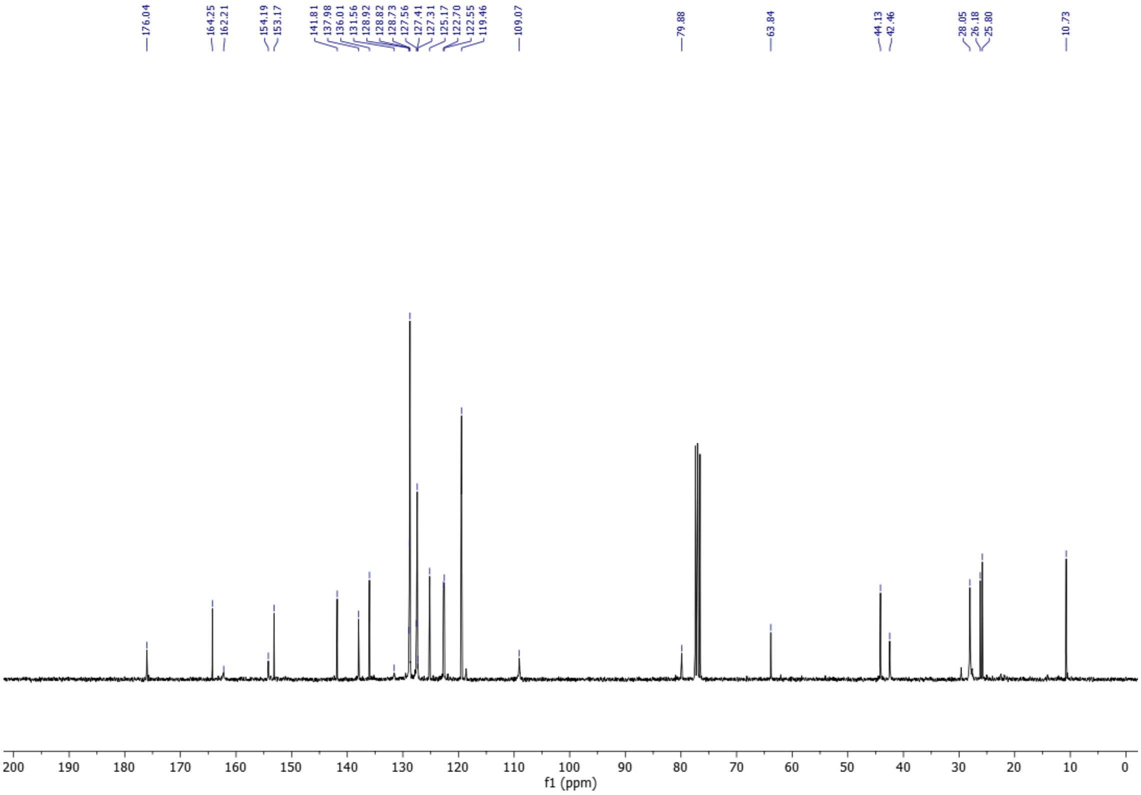

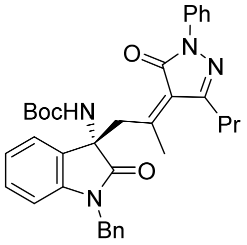

3bc, **^{1}H NMR (300 MHz, CDCl$_3$)**

^{13}C NMR (75 MHz, CDCl$_3$)

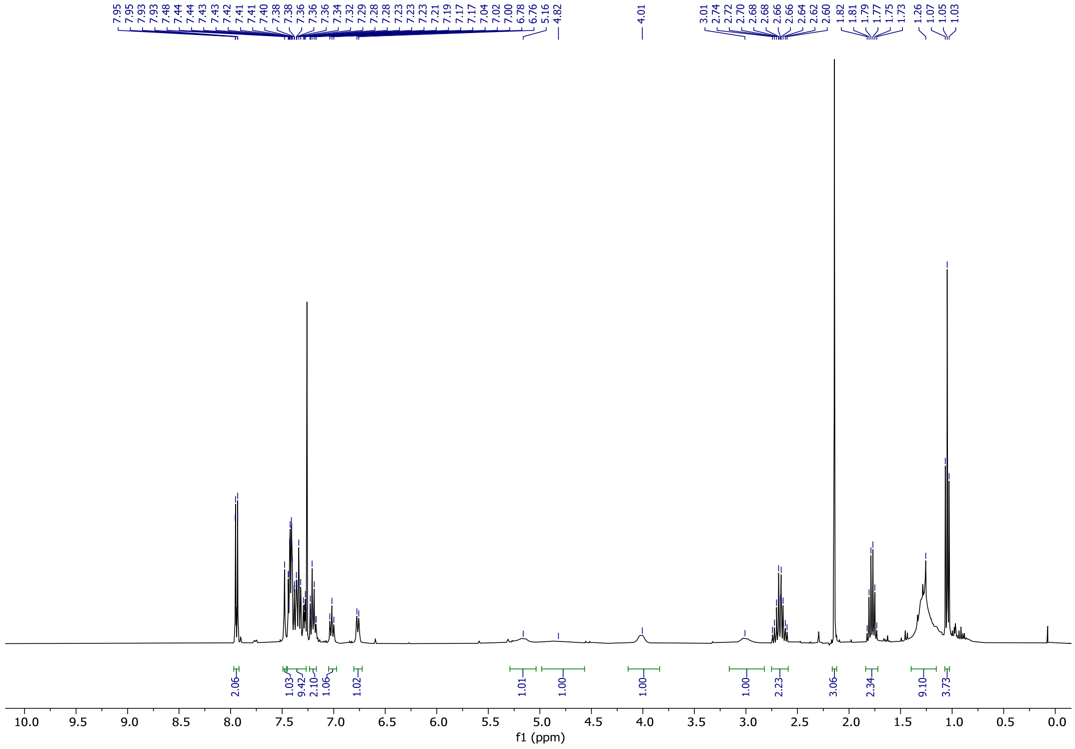

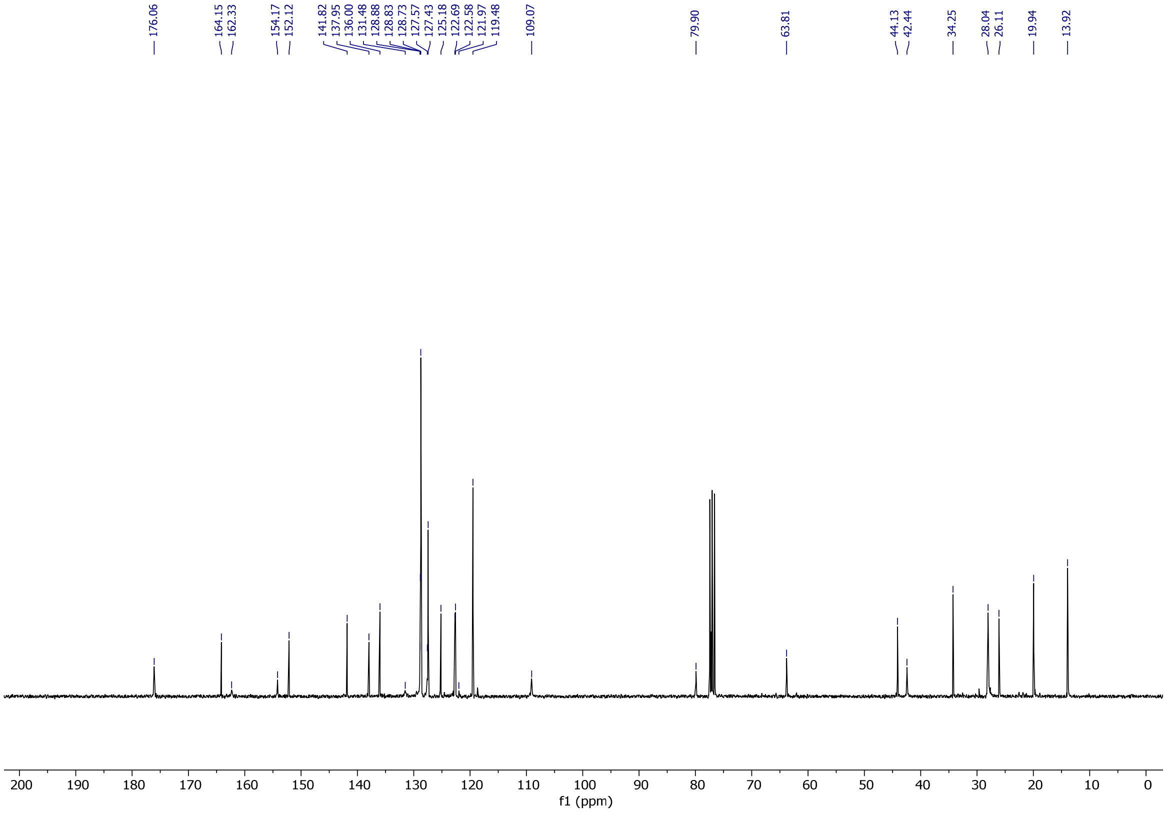

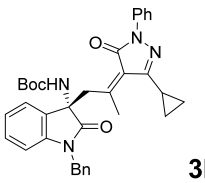

3bd, ^{1}H NMR (300 MHz, $CDCl_3$)
^{13}C NMR (75 MHz, $CDCl_3$)

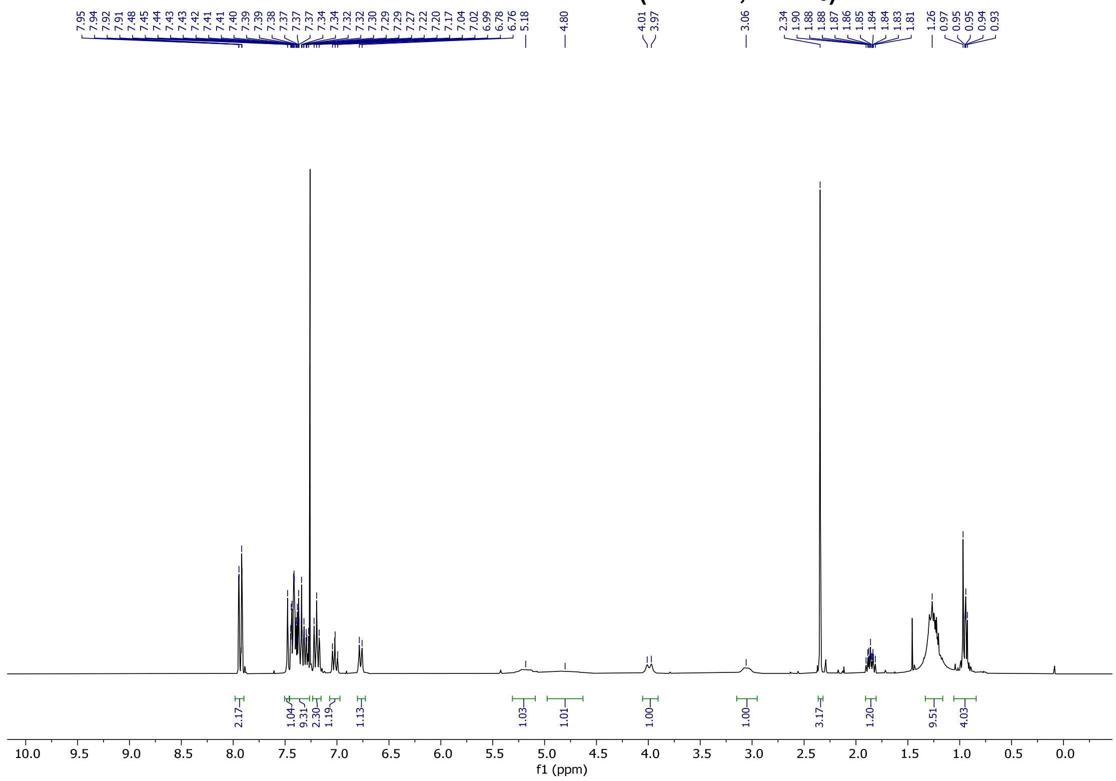

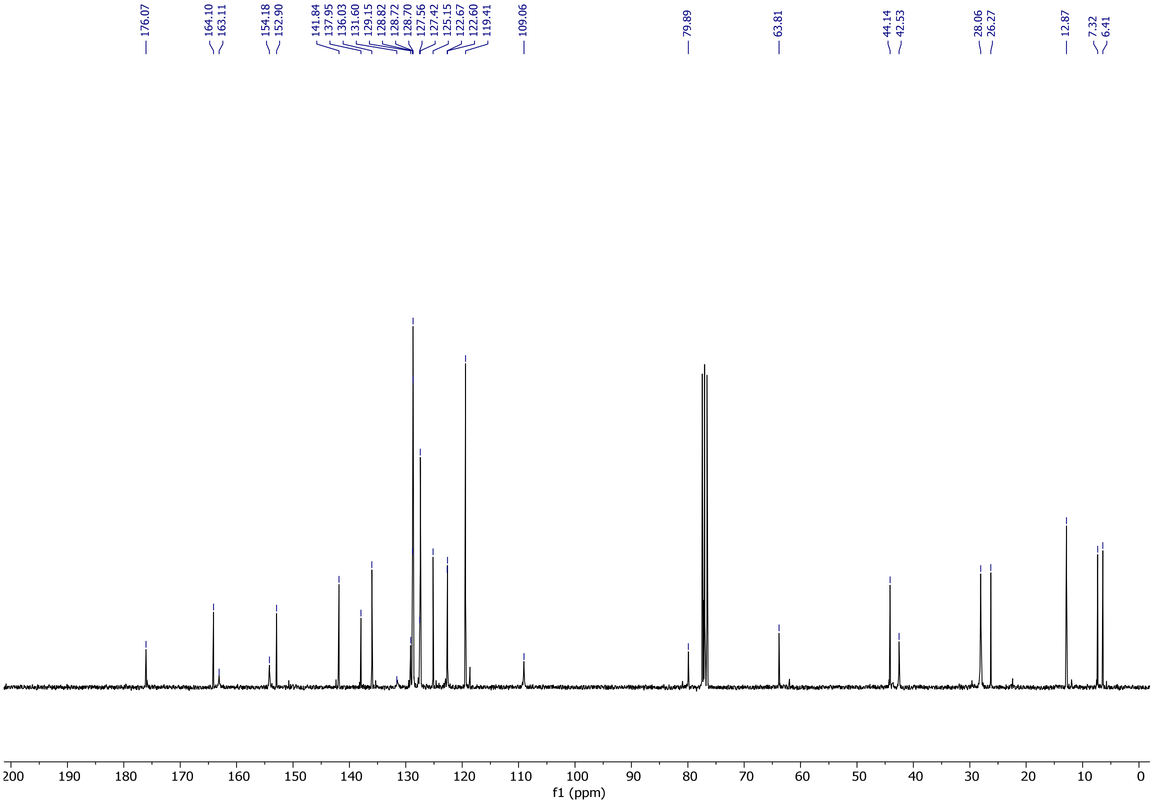

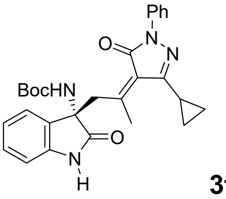

3fd, ^{1}H NMR (300 MHz, $CDCl_3$)
^{13}C NMR (75 MHz, $CDCl_3$)

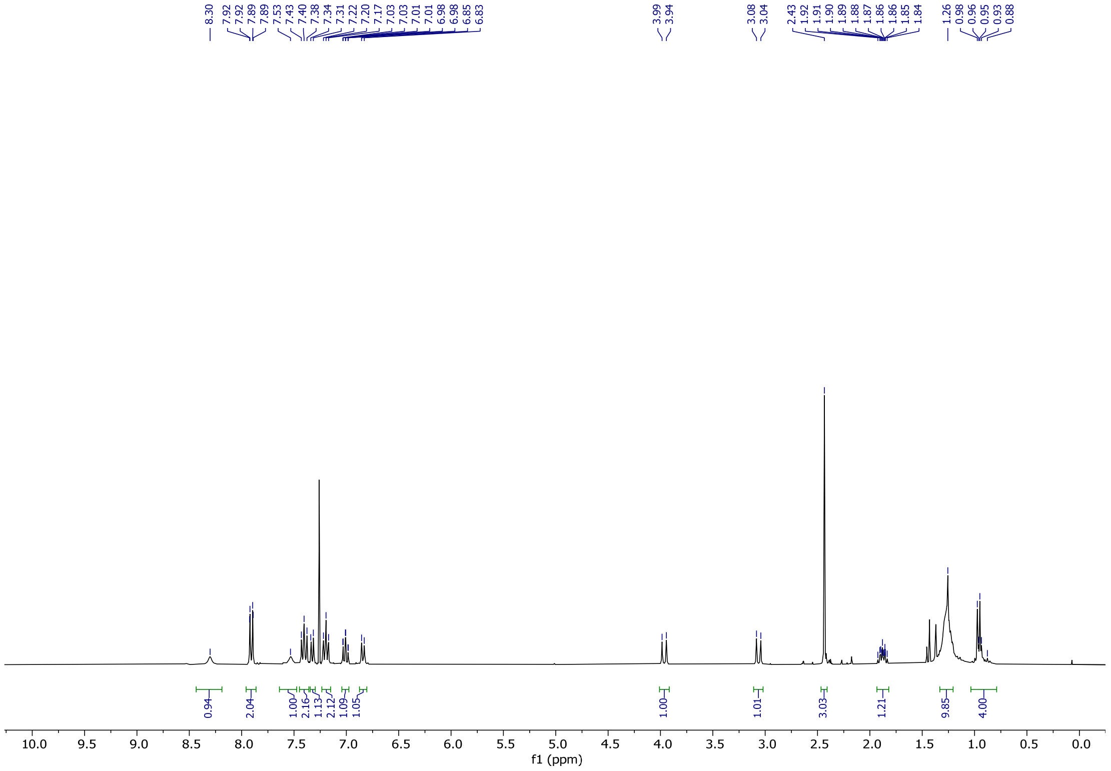

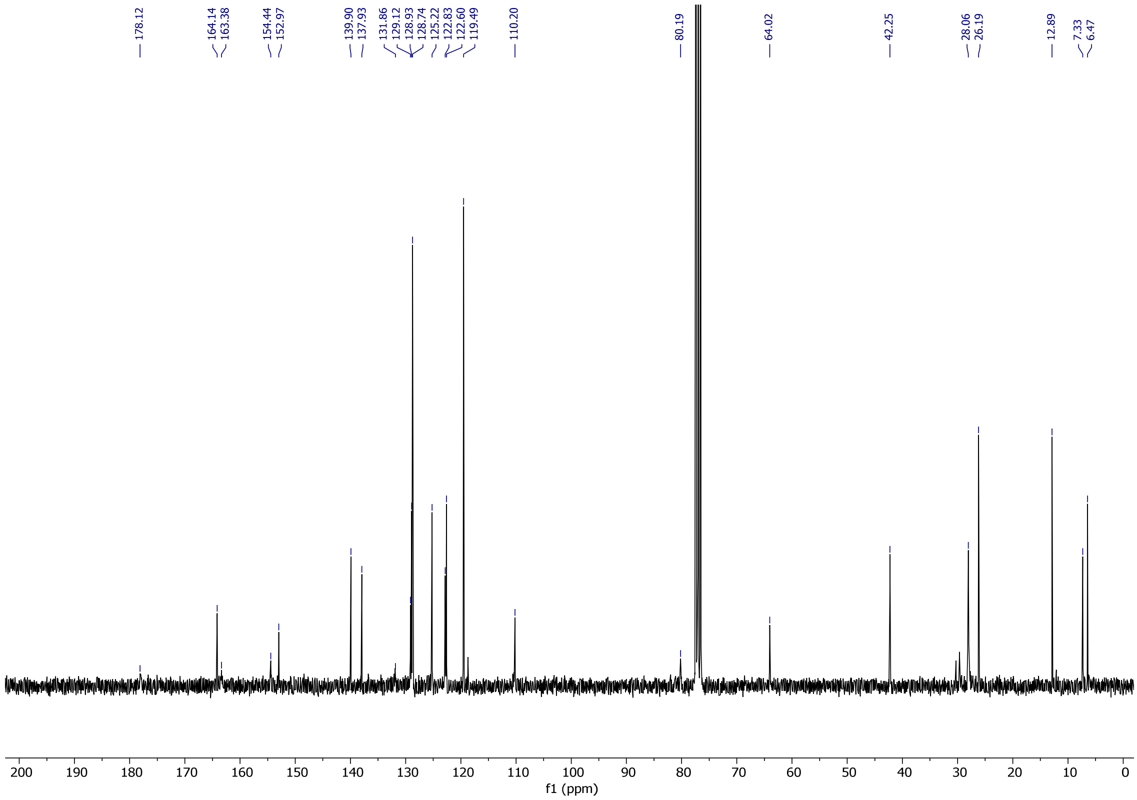

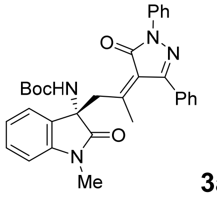

3ae, ^{1}H NMR (300 MHz, CDCl$_3$)
^{13}C NMR (75 MHz, CDCl$_3$)

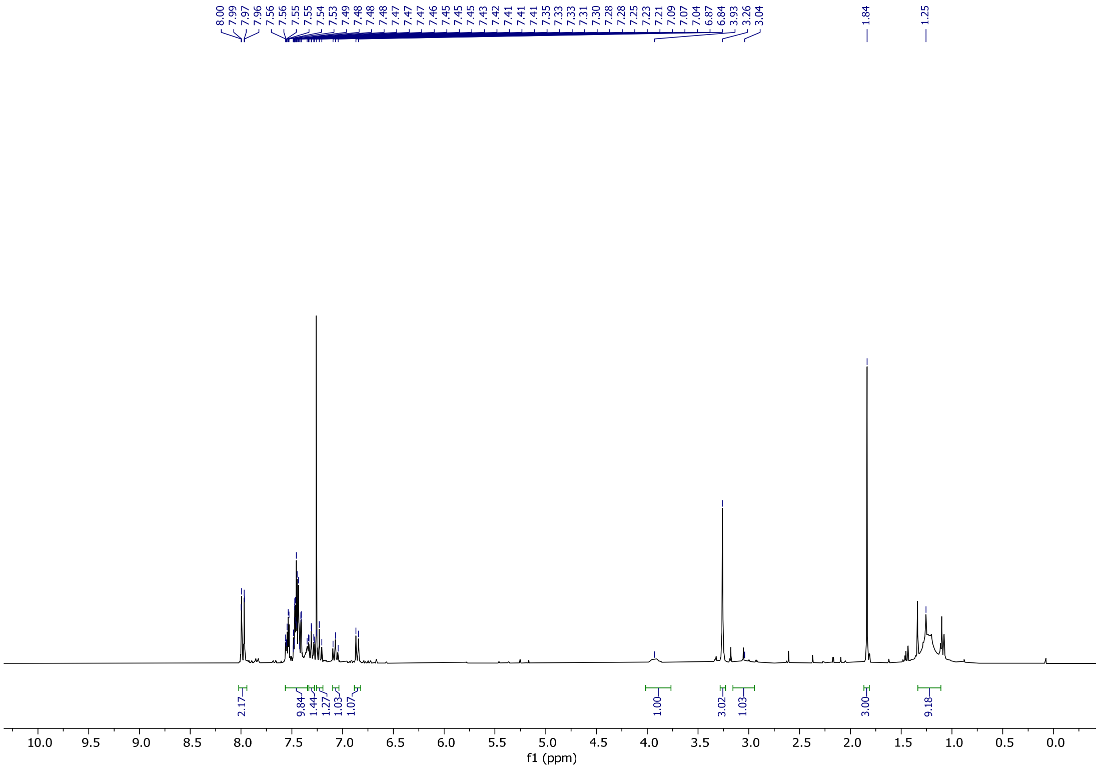

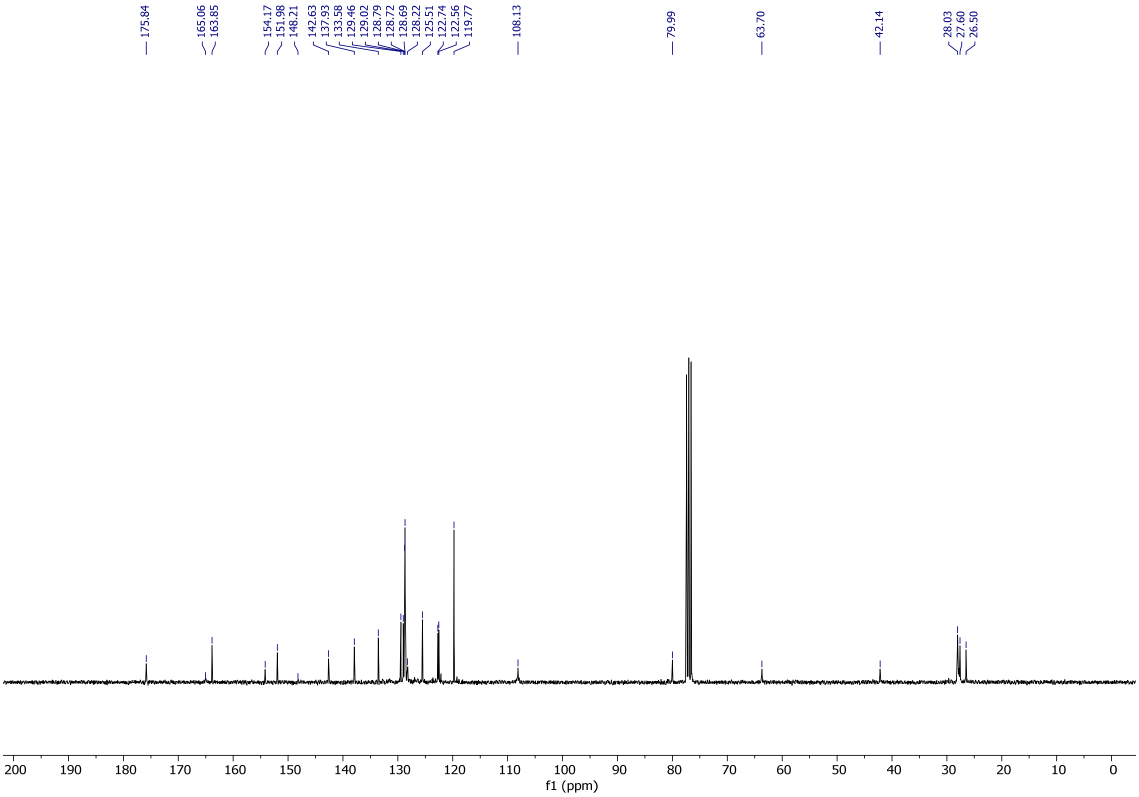

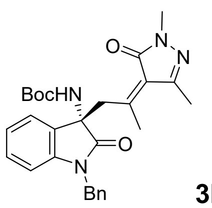

3bf, ^{1}H NMR (300 MHz, $CDCl_3$)
^{13}C NMR (75 MHz, $CDCl_3$)

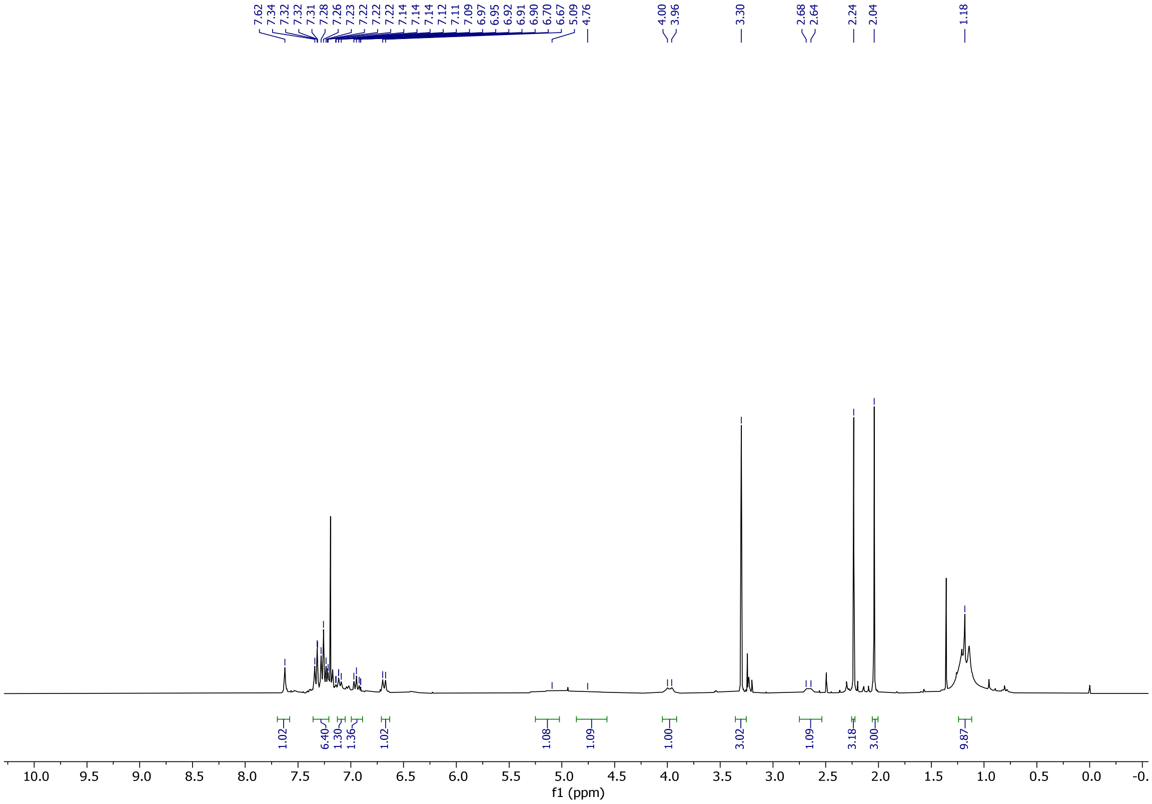

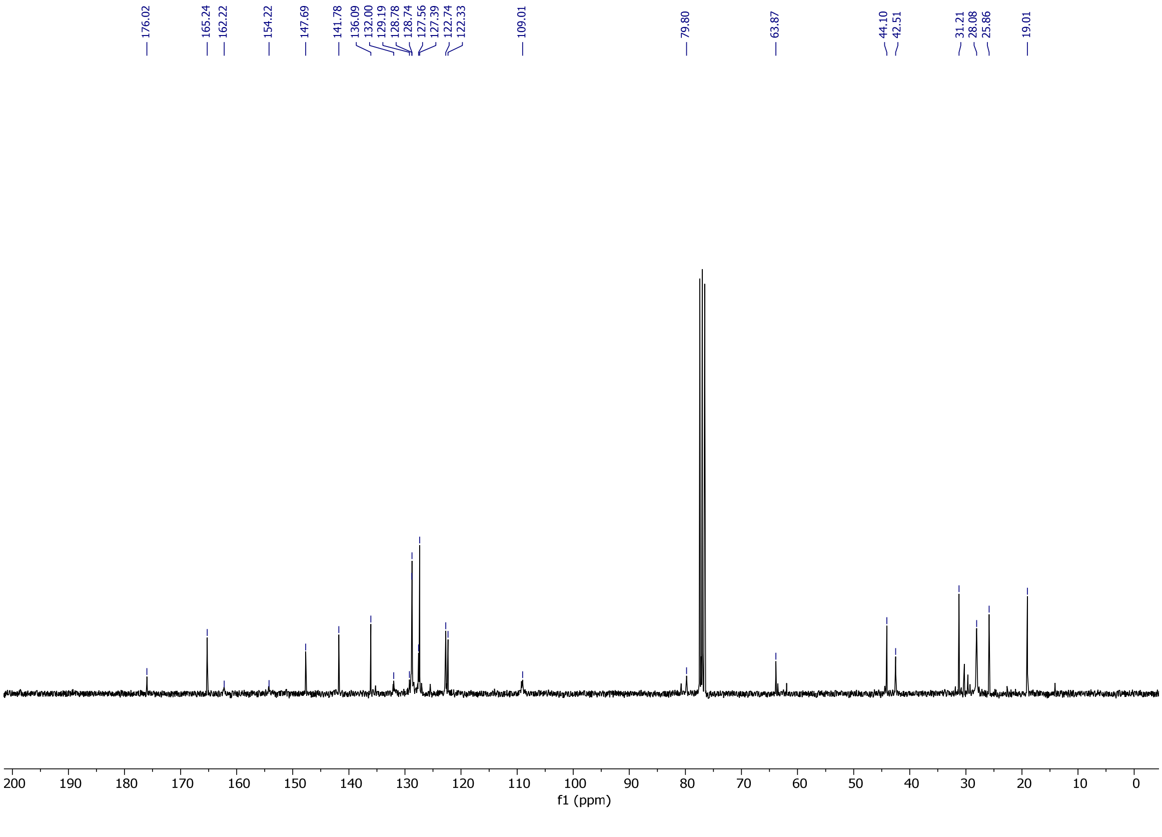

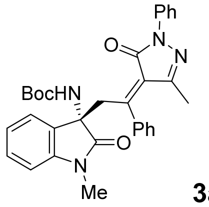

3ag, ^{1}H NMR (300 MHz, $CDCl_3$)
^{13}C NMR (75 MHz, $CDCl_3$)

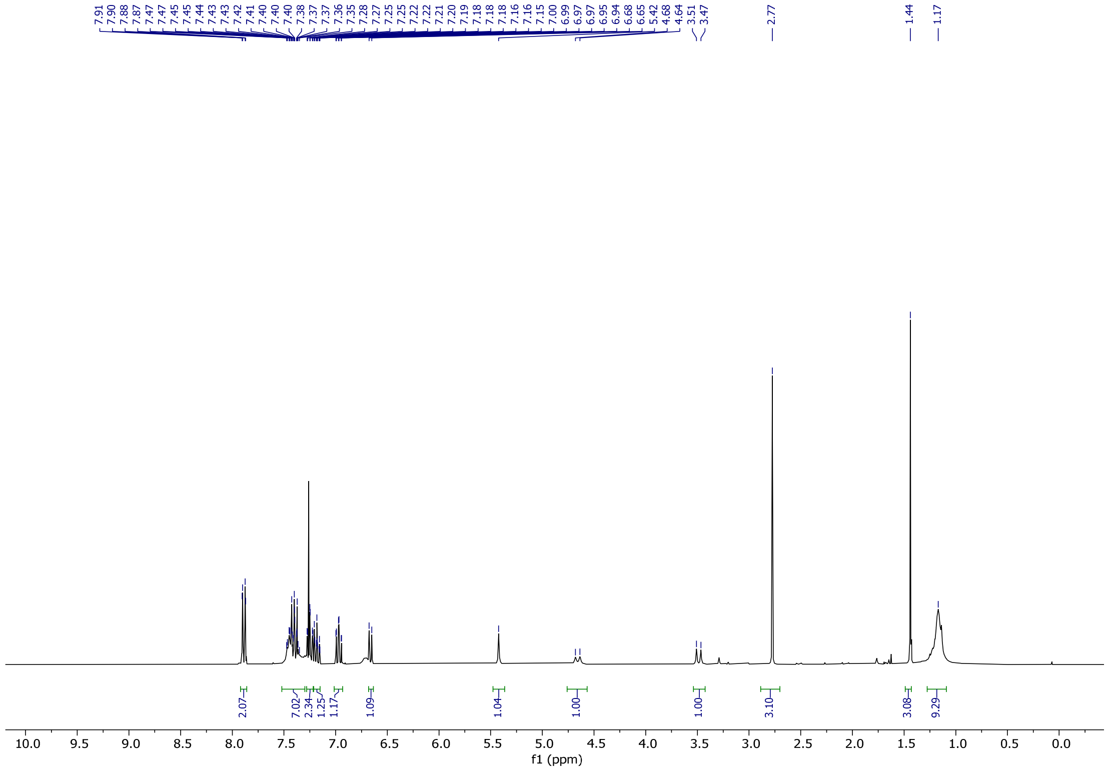

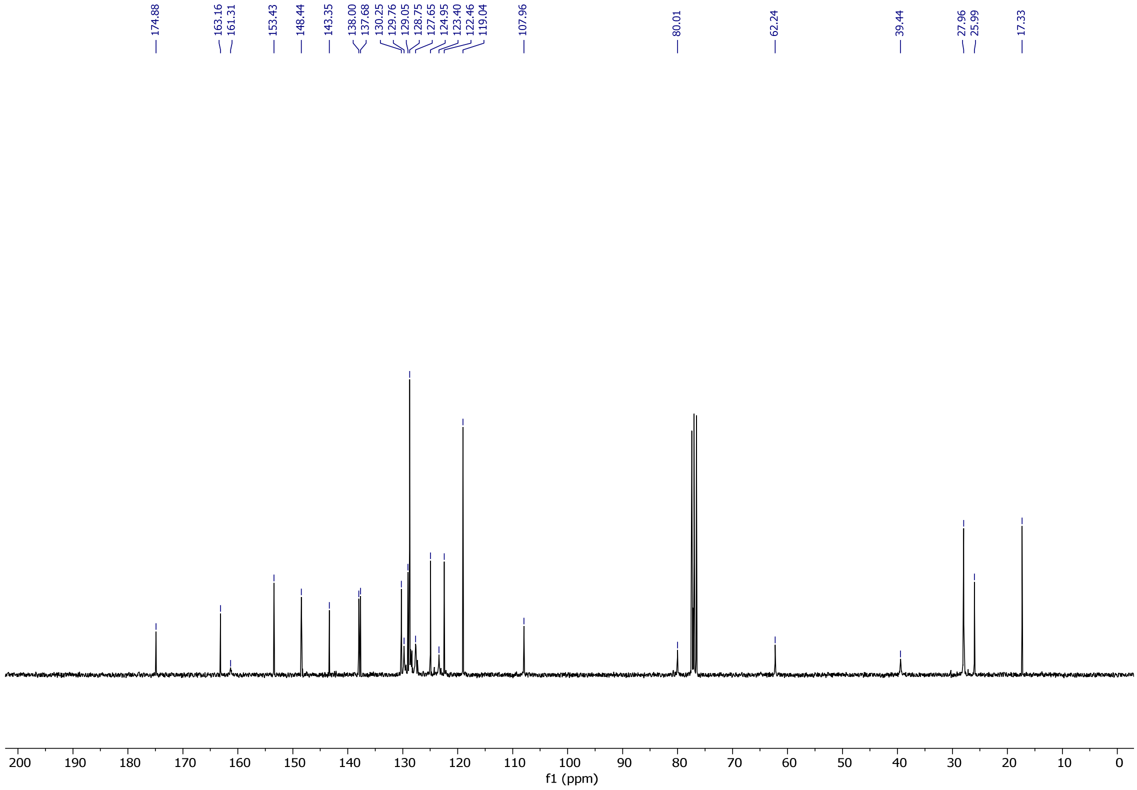

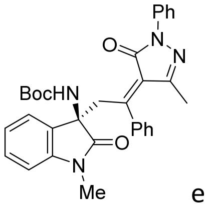

ent-**3ag**, **^{1}H NMR (300 MHz, CDCl$_3$)**

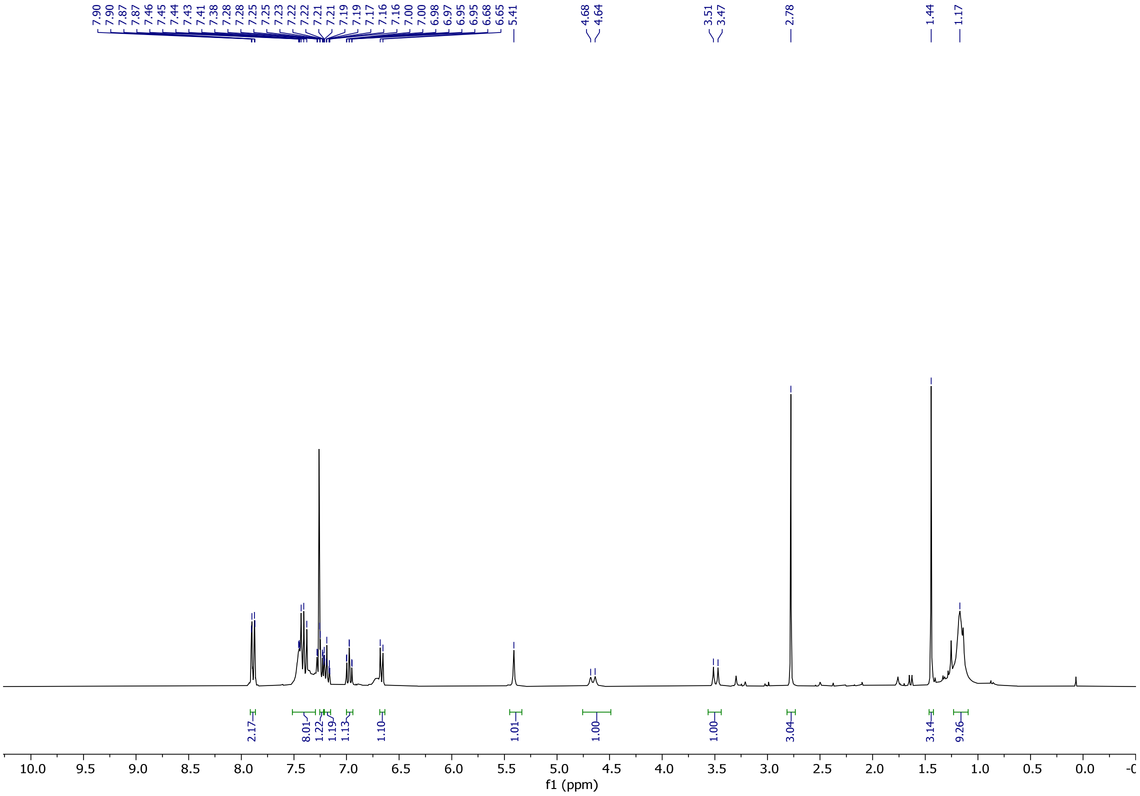

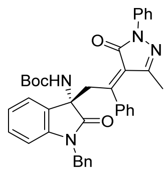

3bg, ^{1}H NMR (300 MHz, CDCl$_3$)
^{13}C NMR (75 MHz, CDCl$_3$)

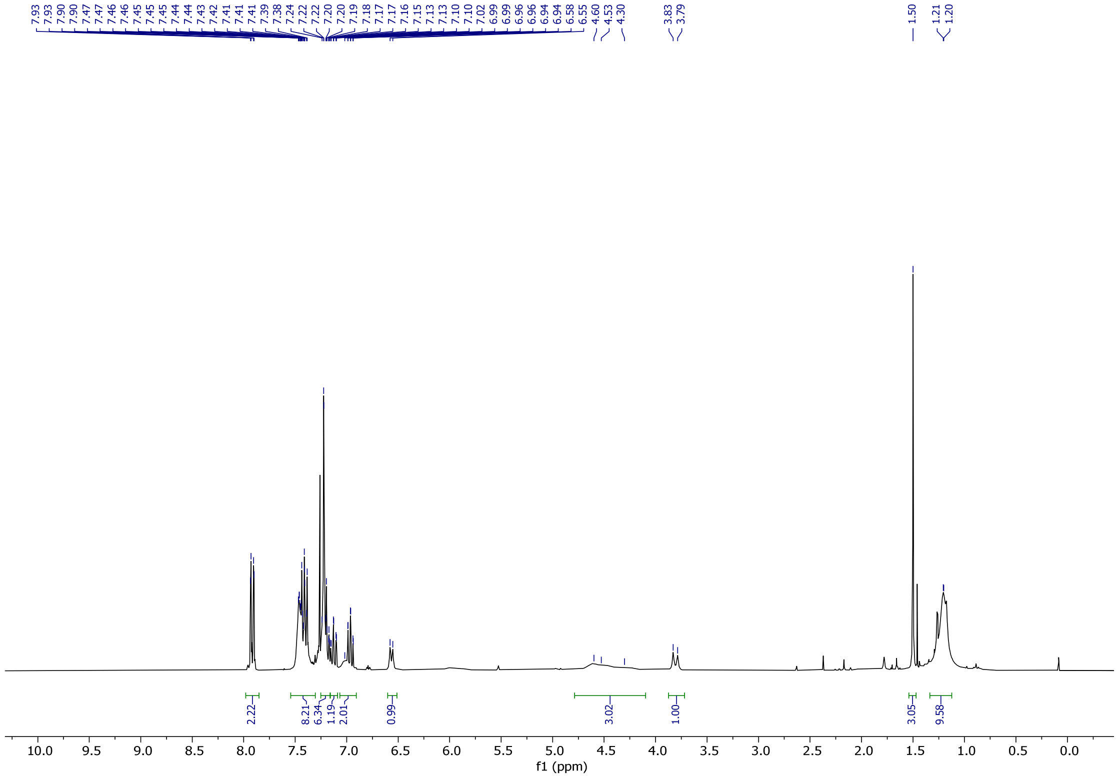

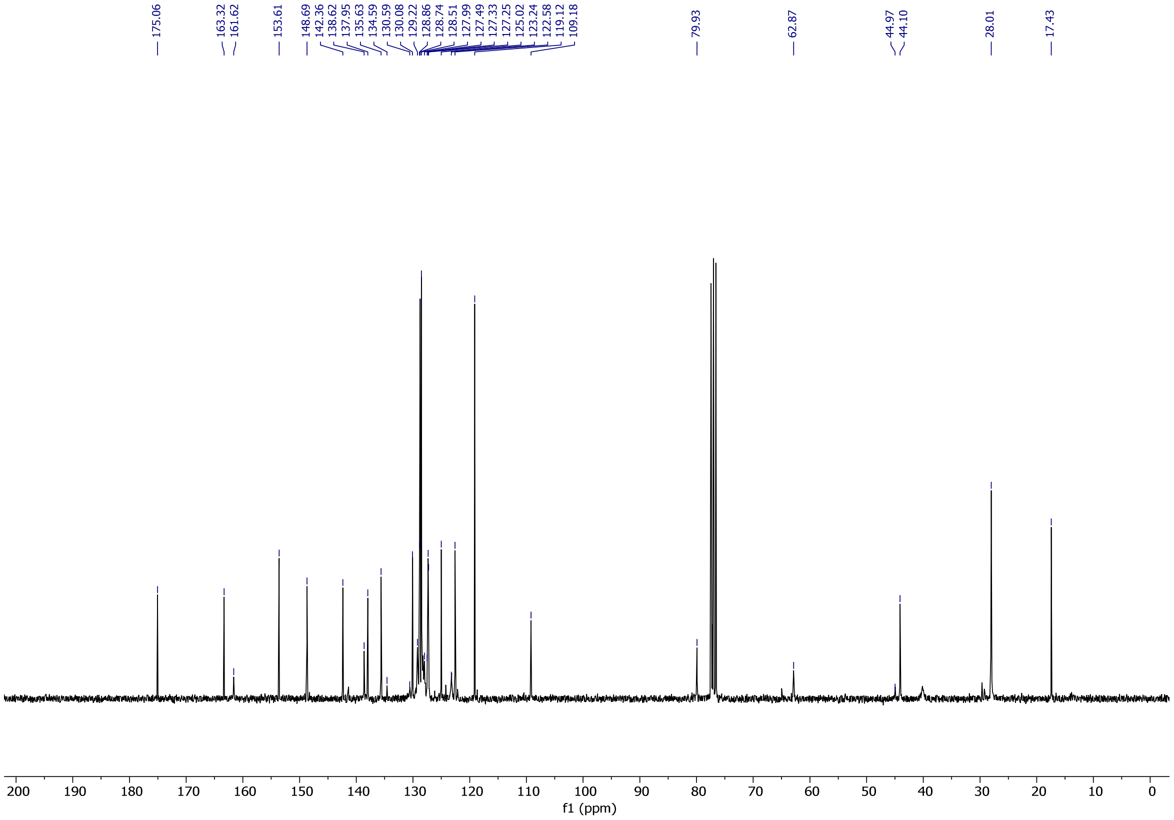

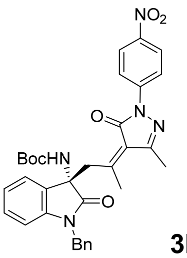

3bh, ^{1}H NMR (300 MHz, $CDCl_3$)

^{13}C NMR (75 MHz, $CDCl_3$)

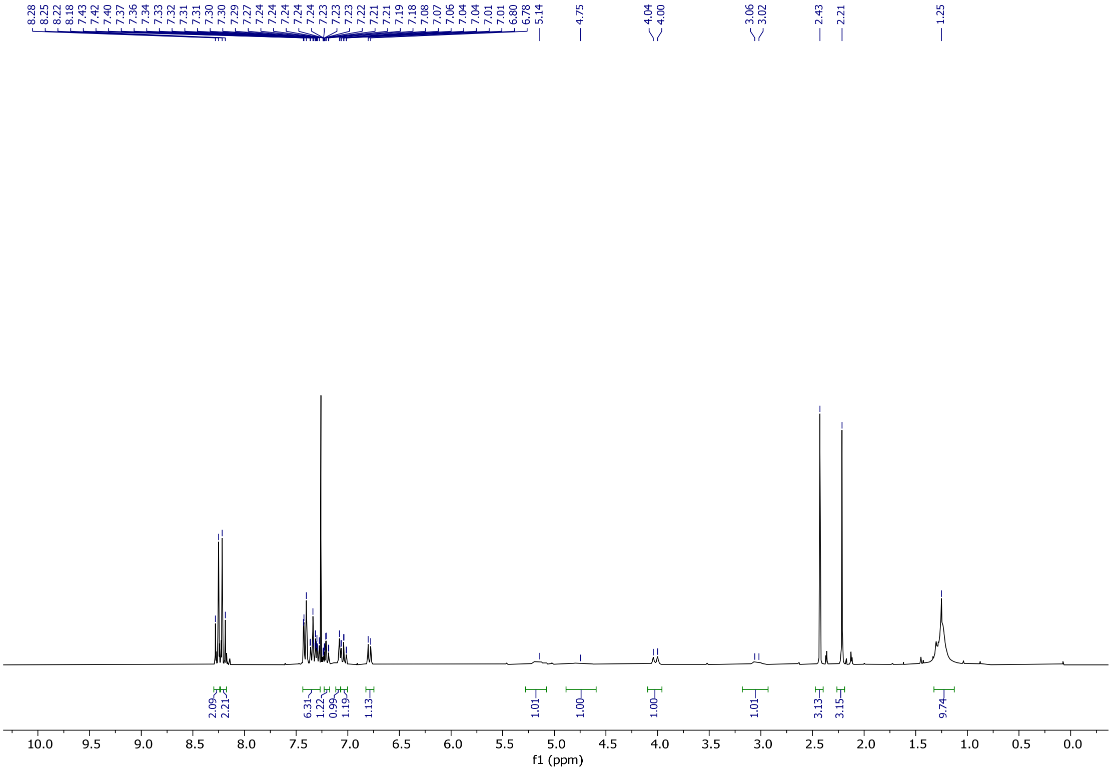

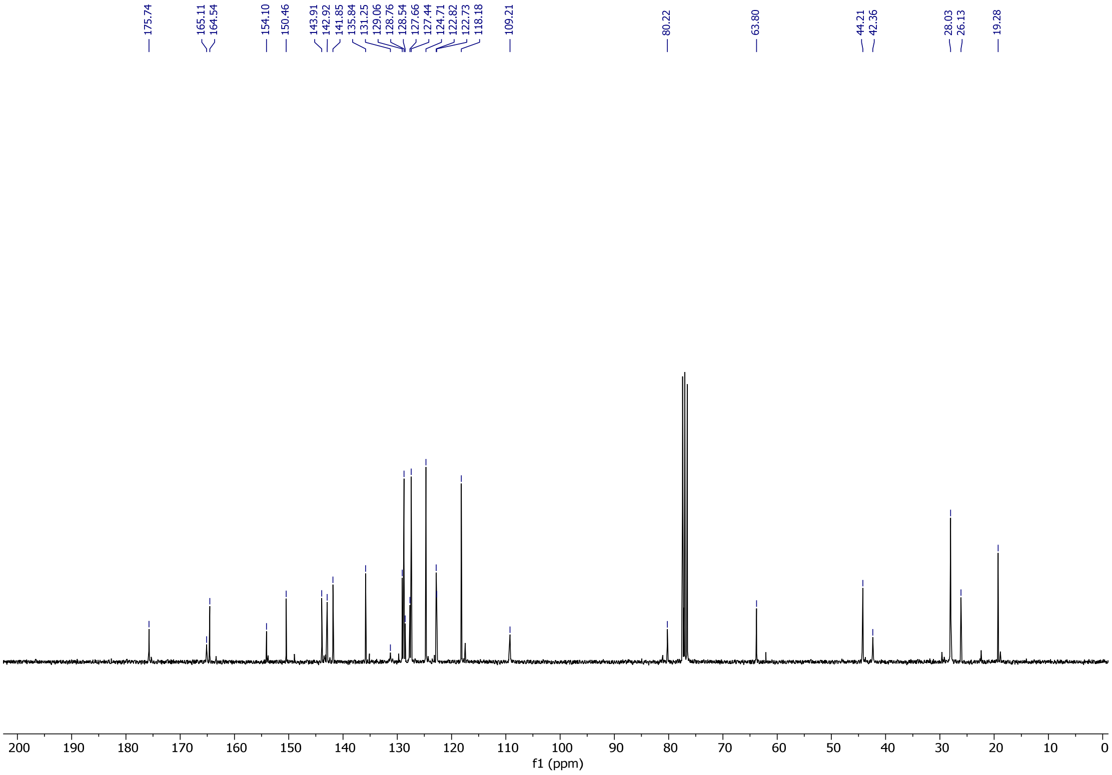

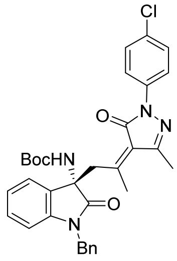

3bi, ¹H NMR (300 MHz, CDCl₃)
¹³C NMR (75 MHz, CDCl₃)

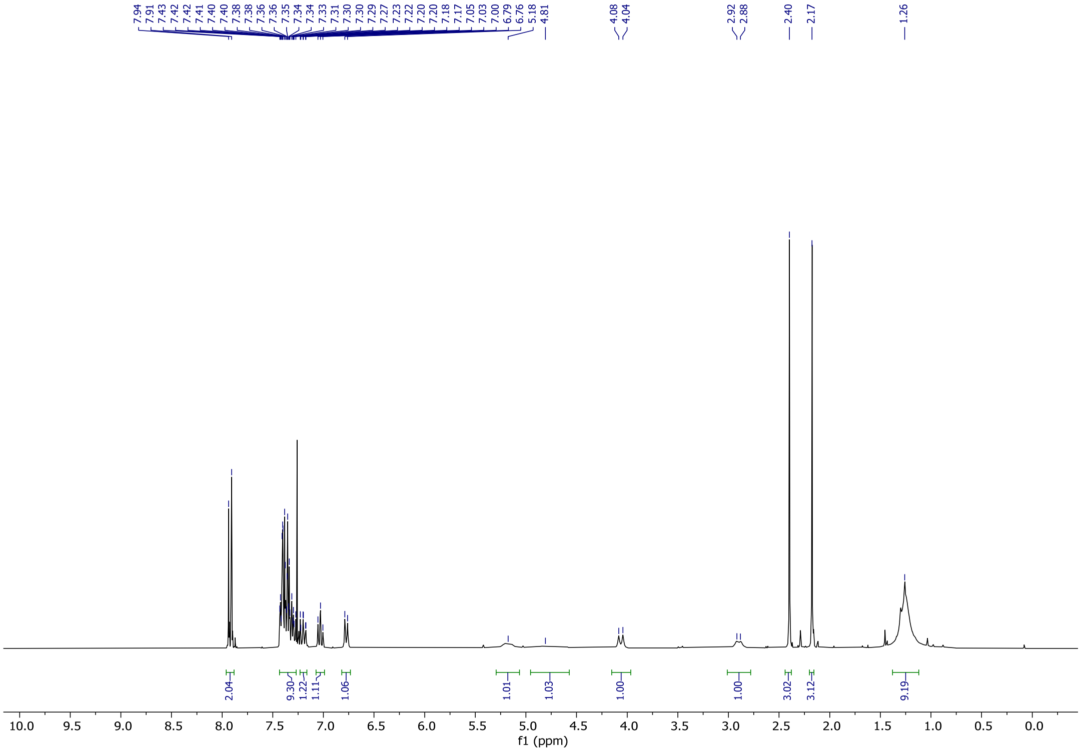

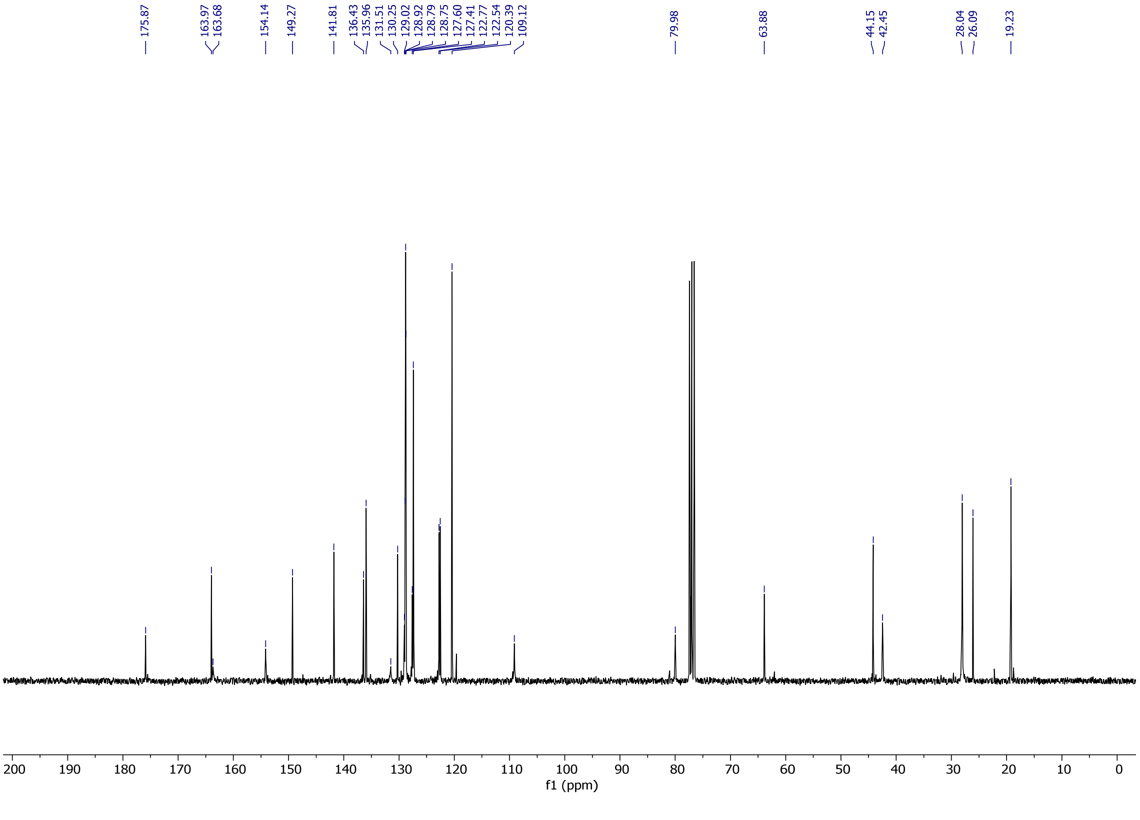

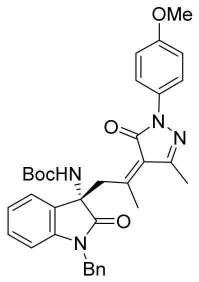

3bj, ^{1}H NMR (300 MHz, $CDCl_3$)
^{13}C NMR (75 MHz, $CDCl_3$)

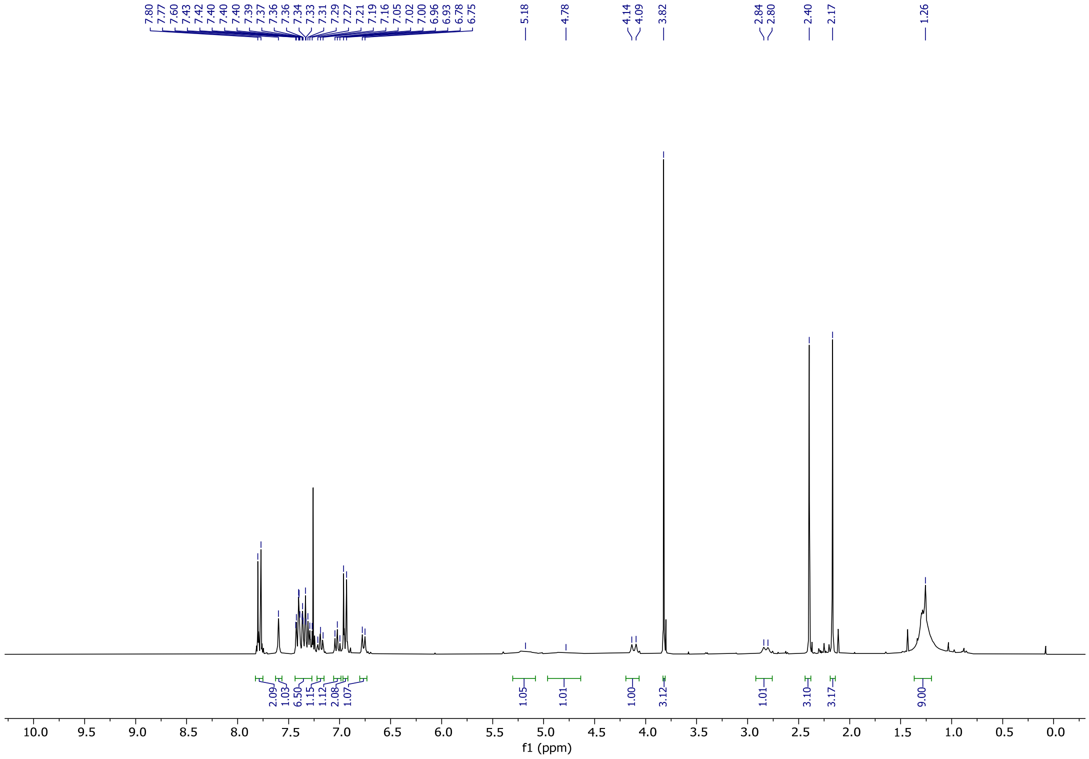

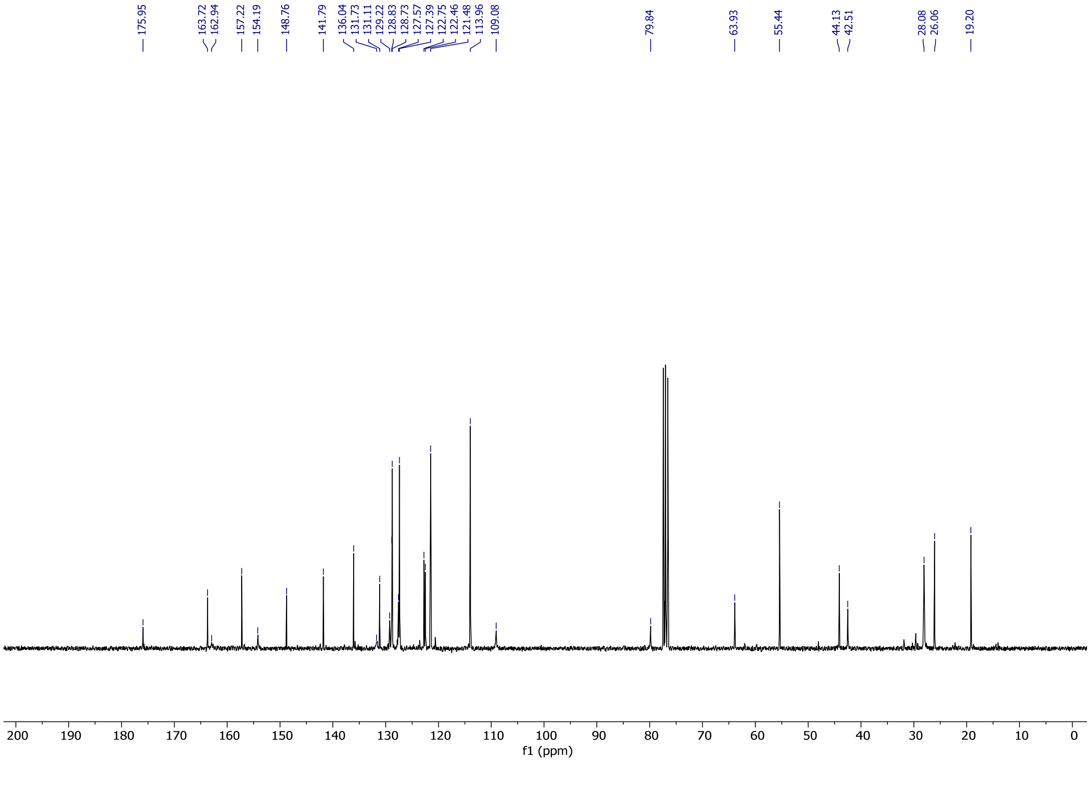

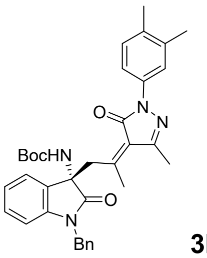

3bk, ¹H NMR (300 MHz, CDCl₃)
¹³C NMR (75 MHz, CDCl₃)

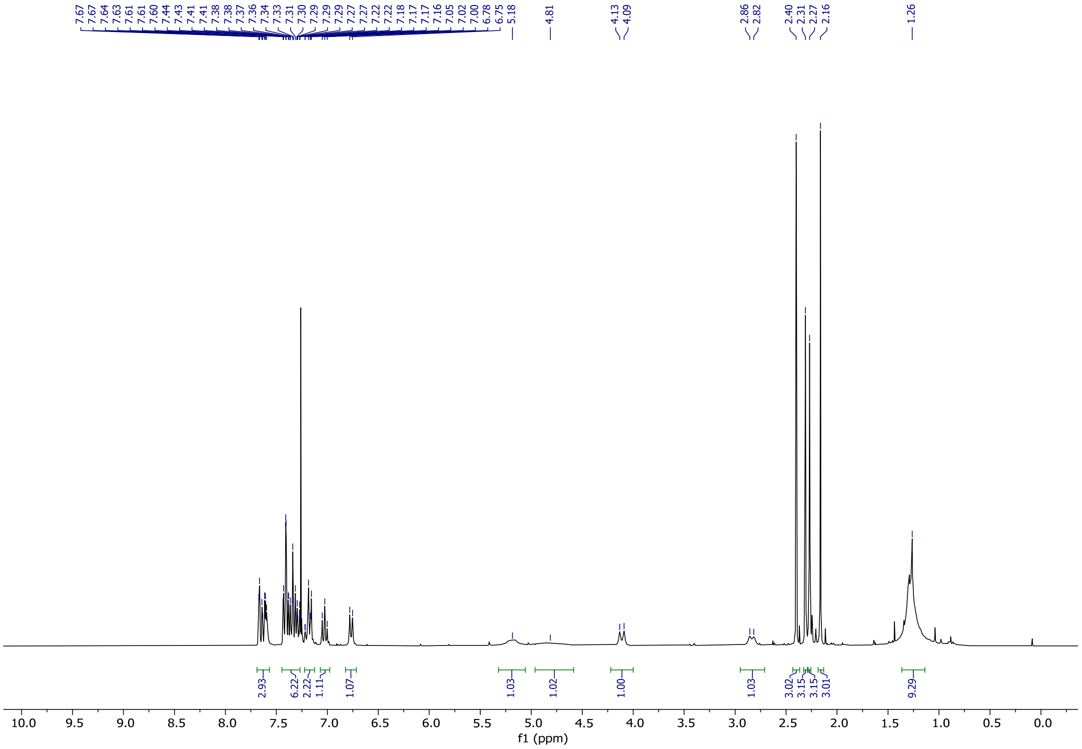

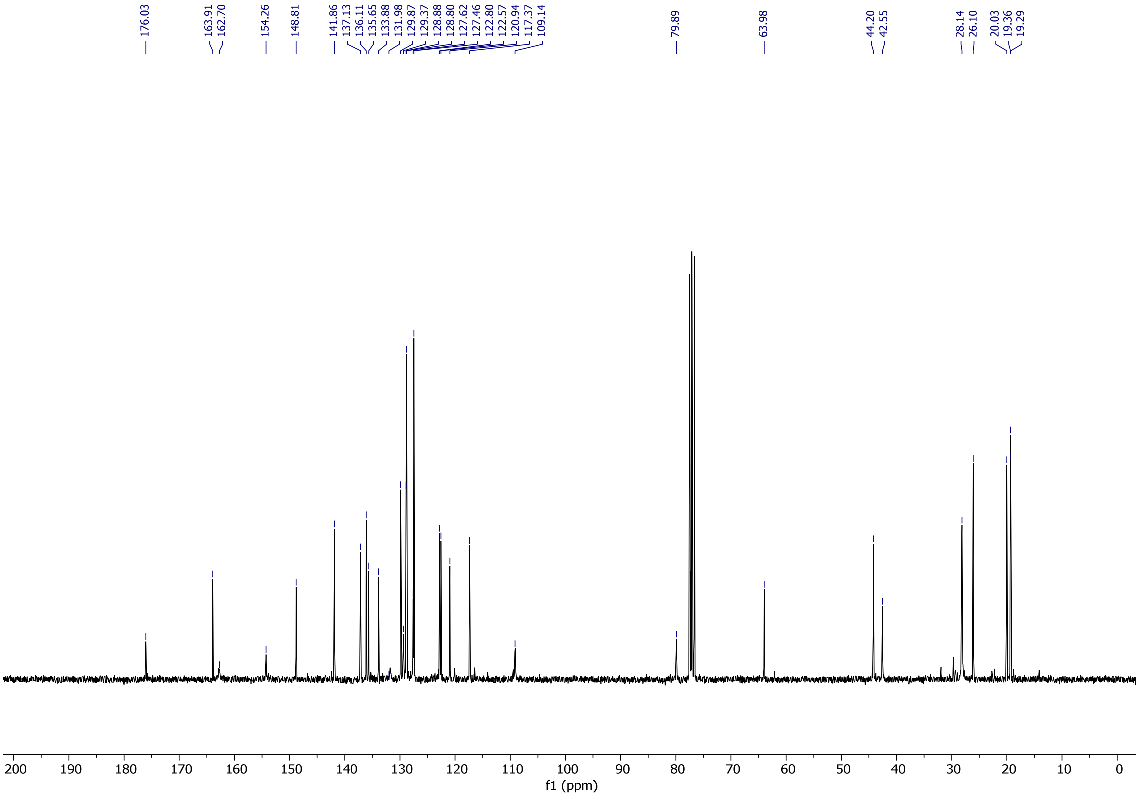

c. FINAL PRODUCT **4** SPECTRA

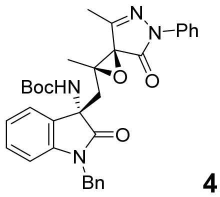

4 (major diastereoisomer), ^{1}H NMR (300 MHz, CDCl$_3$)

^{13}C NMR (75 MHz, CDCl$_3$)

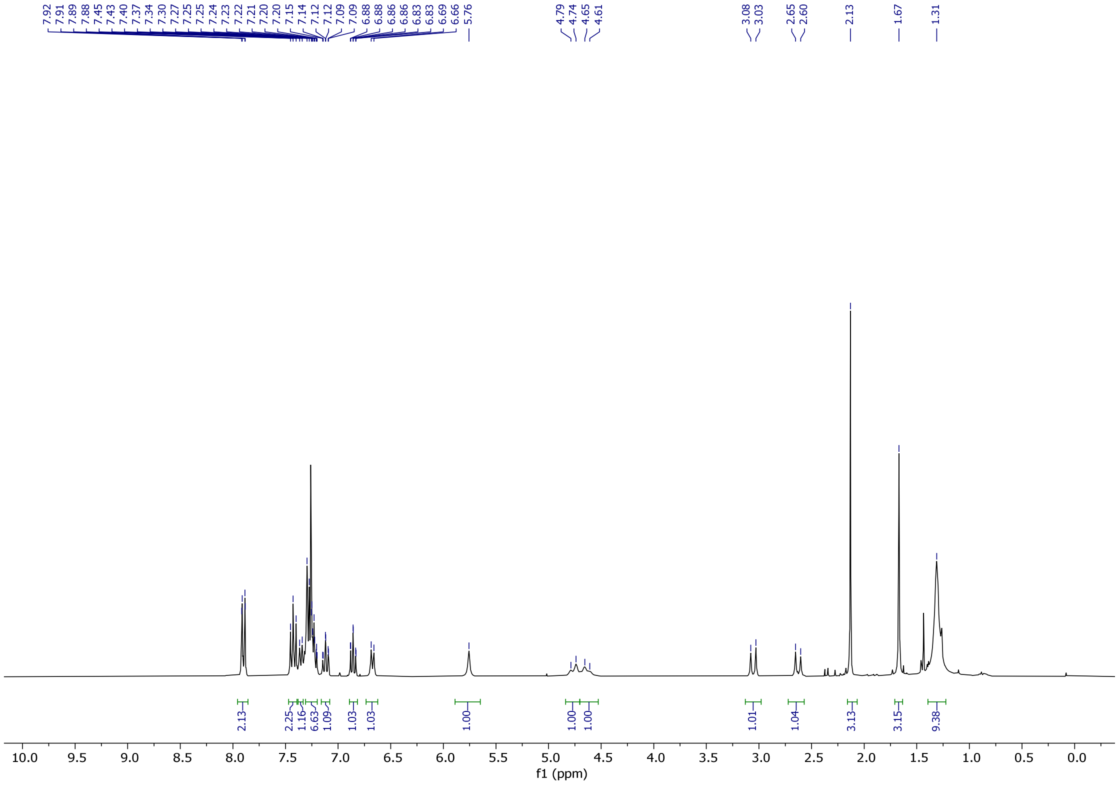

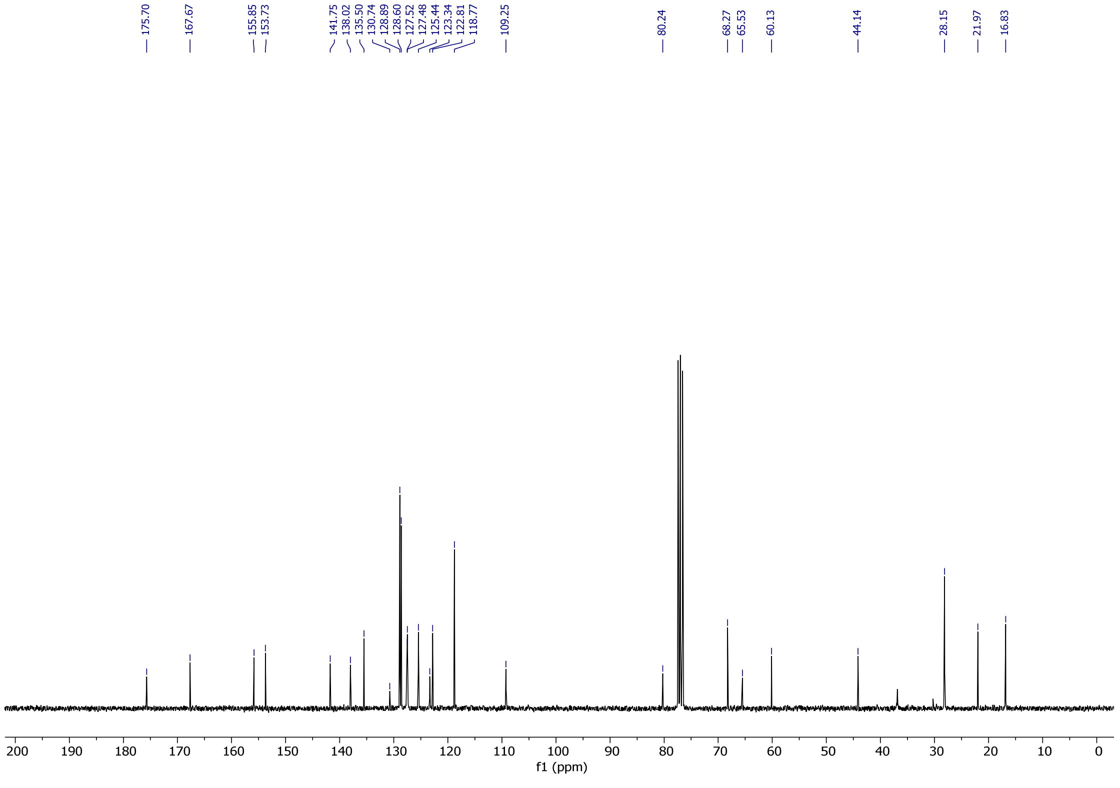

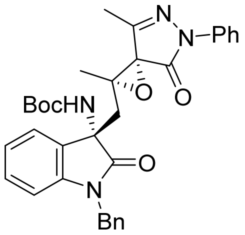

4 (minor diastereoisomer), ^{1}H NMR (300 MHz, $CDCl_3$)
^{13}C NMR (75 MHz, $CDCl_3$)

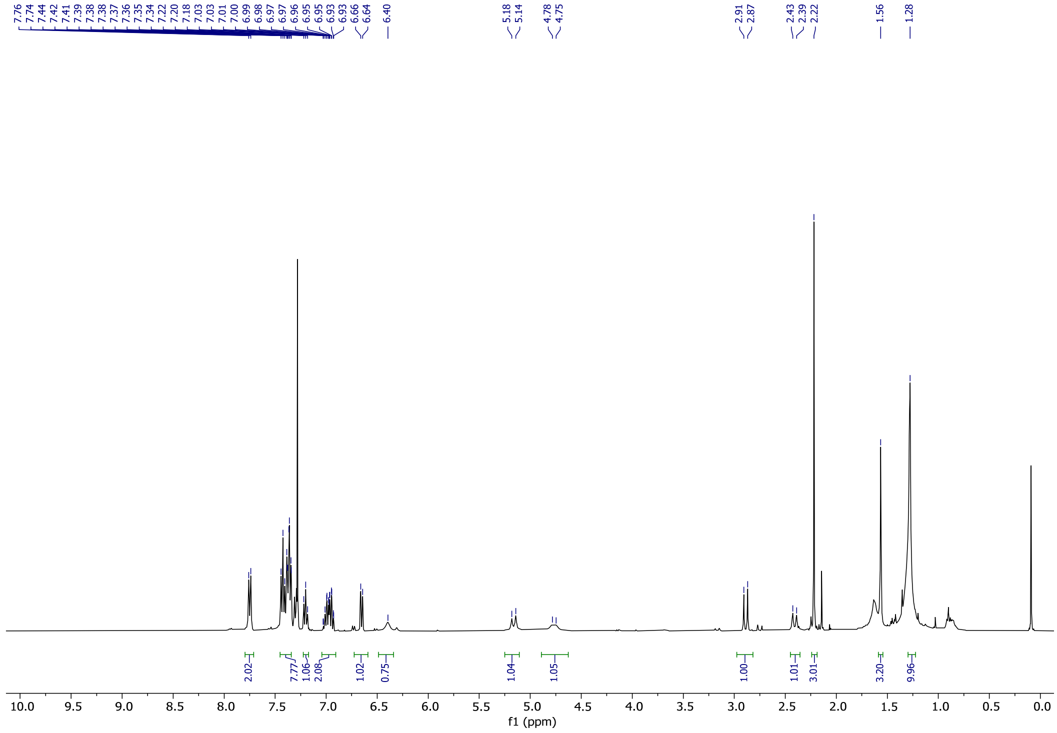

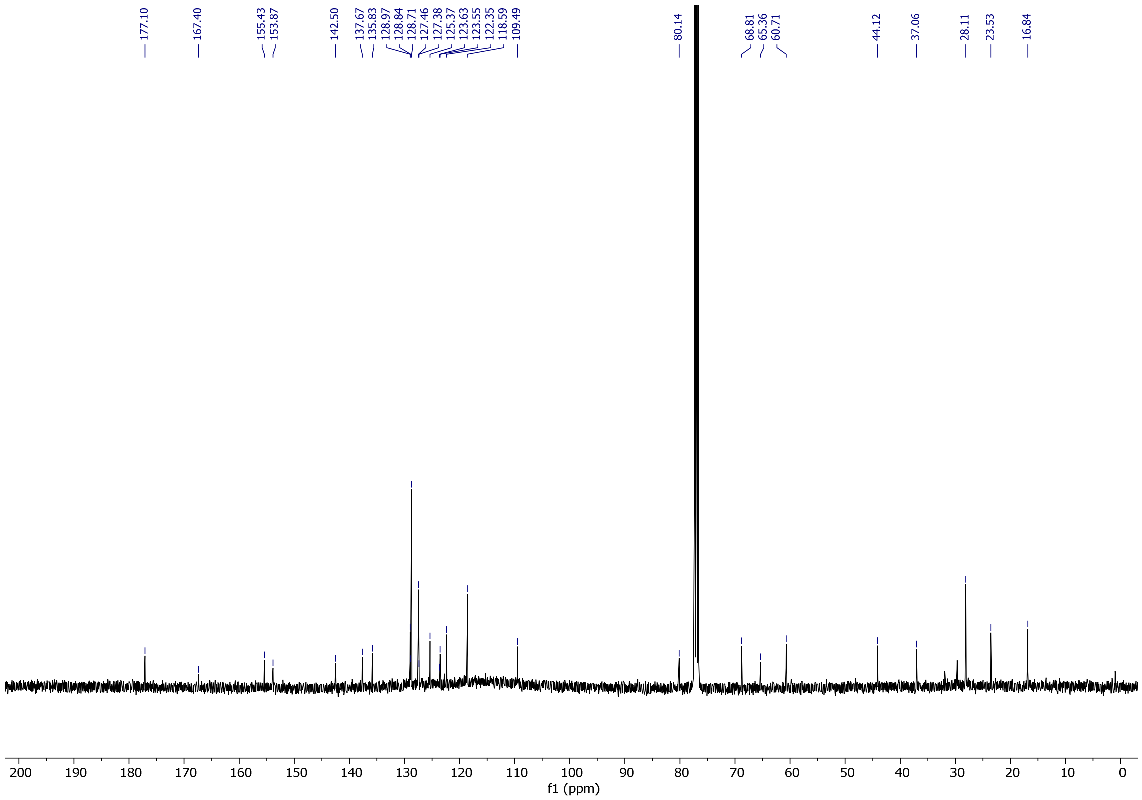

d. FINAL PRODUCT **5** SPECTRA

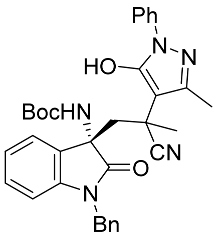

5, ^{1}H NMR (500 MHz, DMSO-*d6*)
^{13}C NMR (126 MHz, DMSO-*d6*)

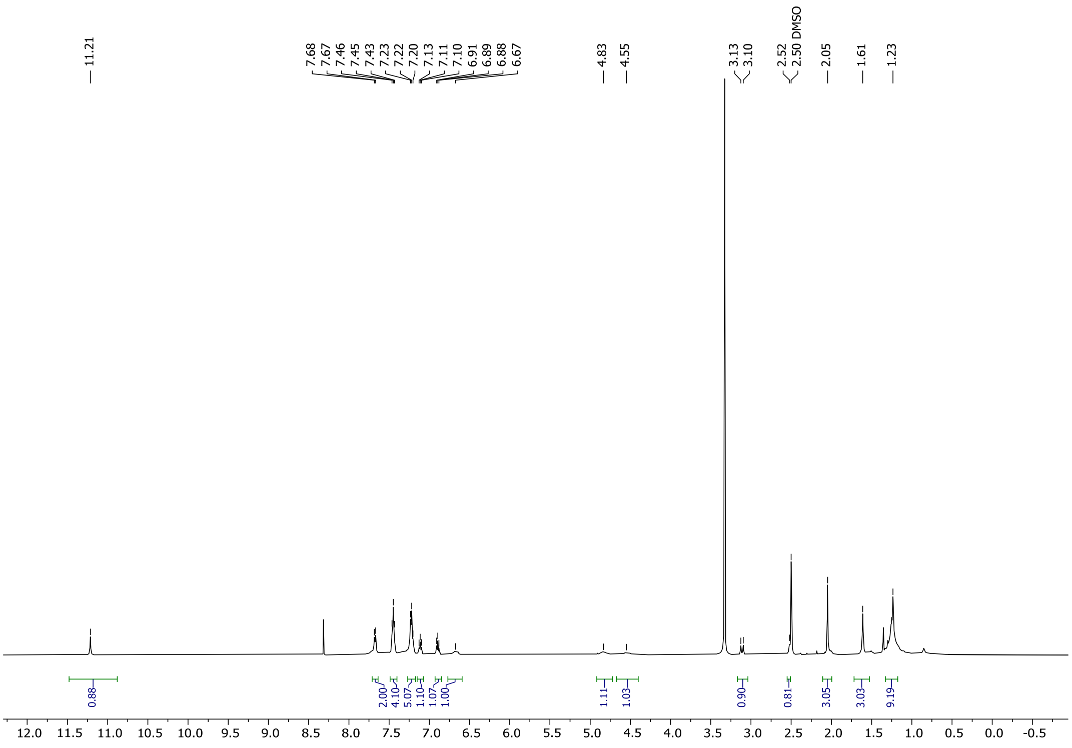

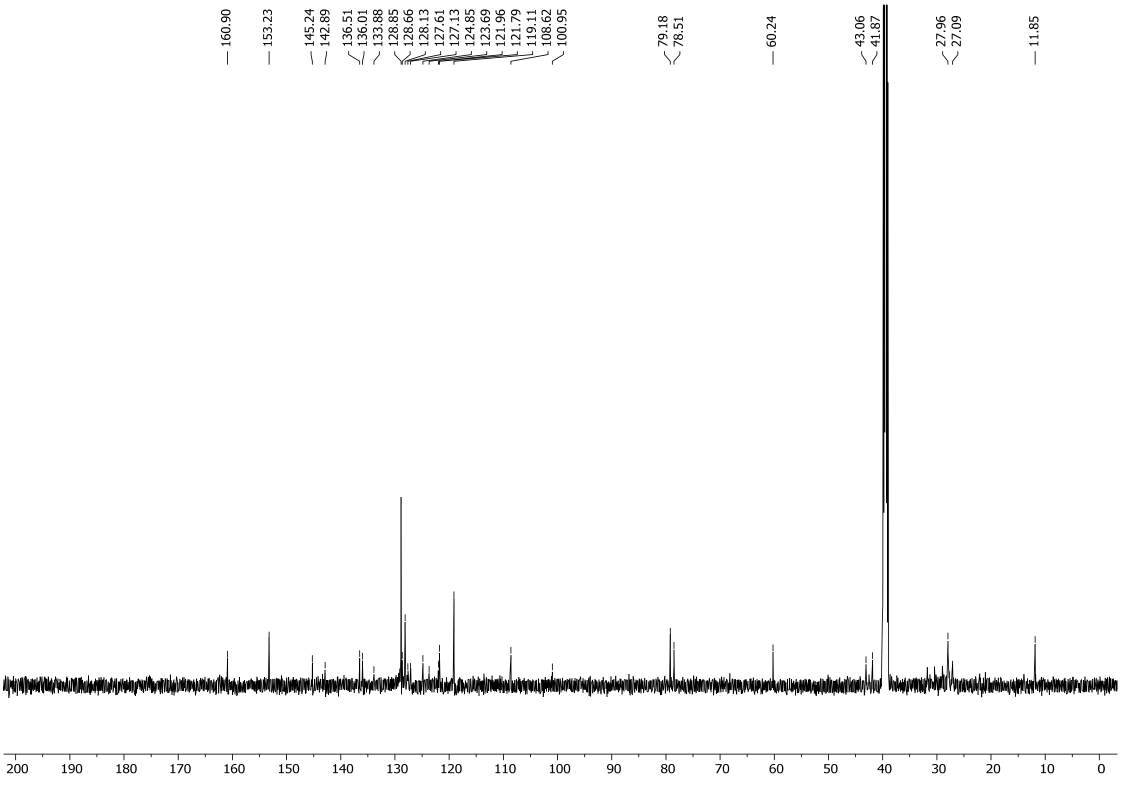

e. FINAL PRODUCT **6** SPECTRA

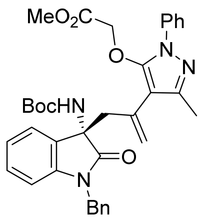

6, ^{1}H NMR (300 MHz, $CDCl_3$)

^{13}C NMR (75 MHz, $CDCl_3$)

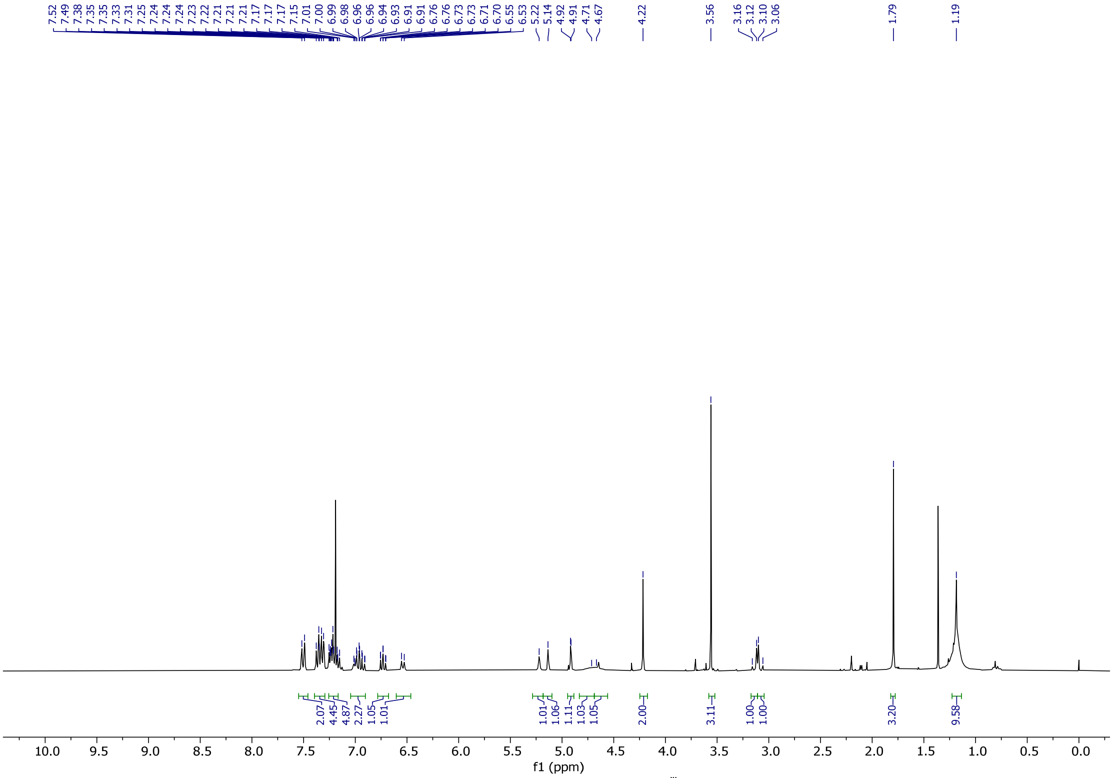

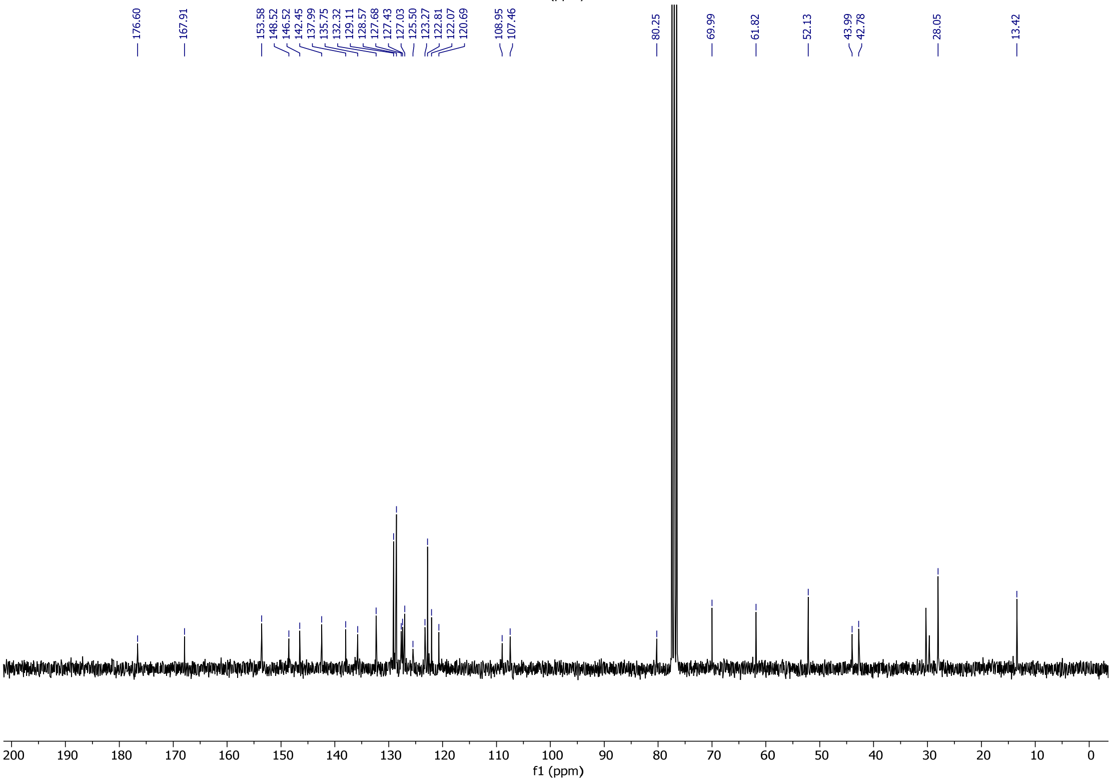

HPLC CHROMATOGRAMS

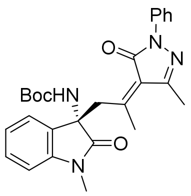

3aa

Sample Name: LC-478 ADH 8020 1mL Vial Number: 1

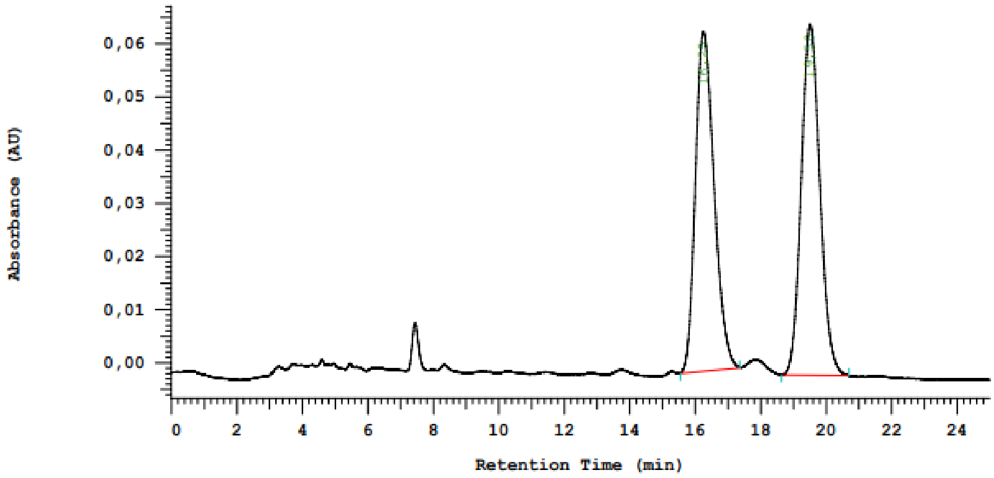

No.	RT	Area	Area %	Name
1	16,25	1267280	49,293	
2	19,50	1303630	50,707	
		2570910	100,000	

Sample Name: LC-485 ADH 8020 1mL Vial Number: 1

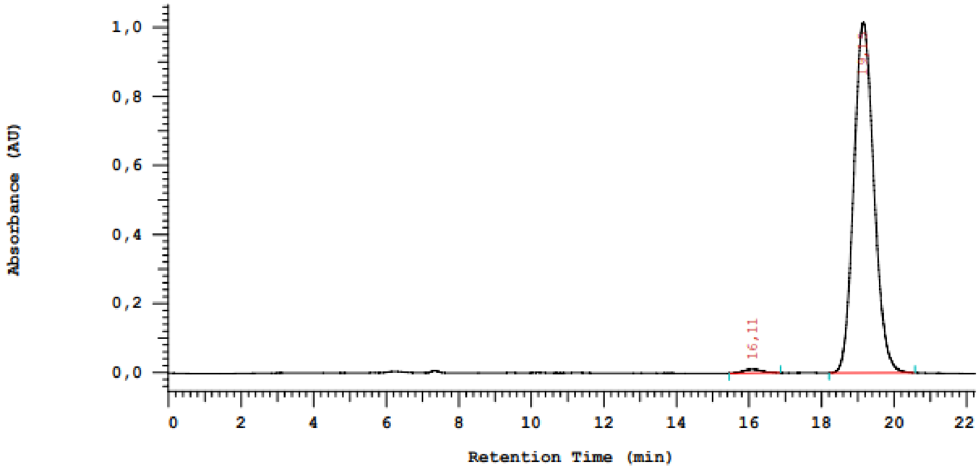

No.	RT	Area	Area %	Name
1	16,11	204840	1,036	
2	19,15	19566910	98,964	
		19771750	100,000	

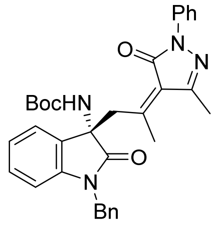

3ba

Sample Name: LC-502 ADH 8020 1mL Vial Number: 1

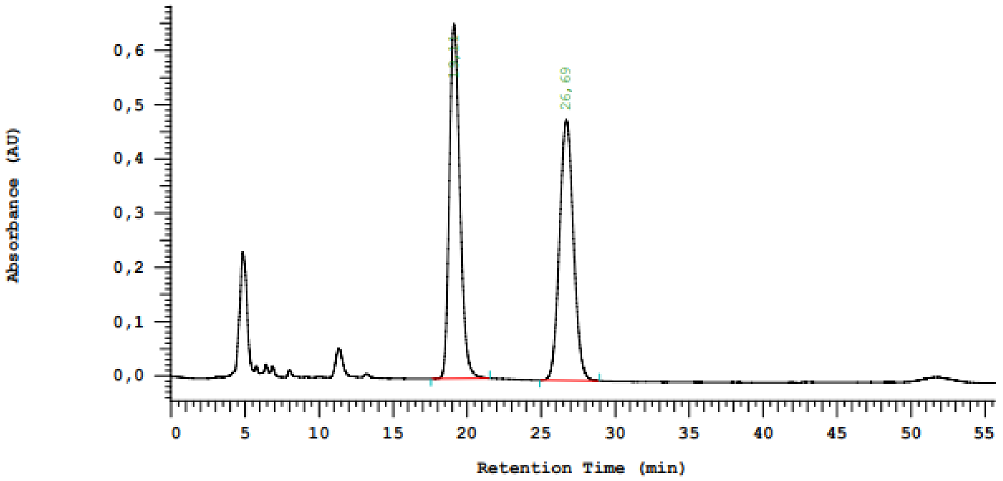

No.	RT	Area	Area %	Name
1	19,11	15772110	50,292	
2	26,69	15588825	49,708	enanti (-)
		31360935	100,000	

Sample Name: LC-522 ADH 8020 1mL Vial Number: 1

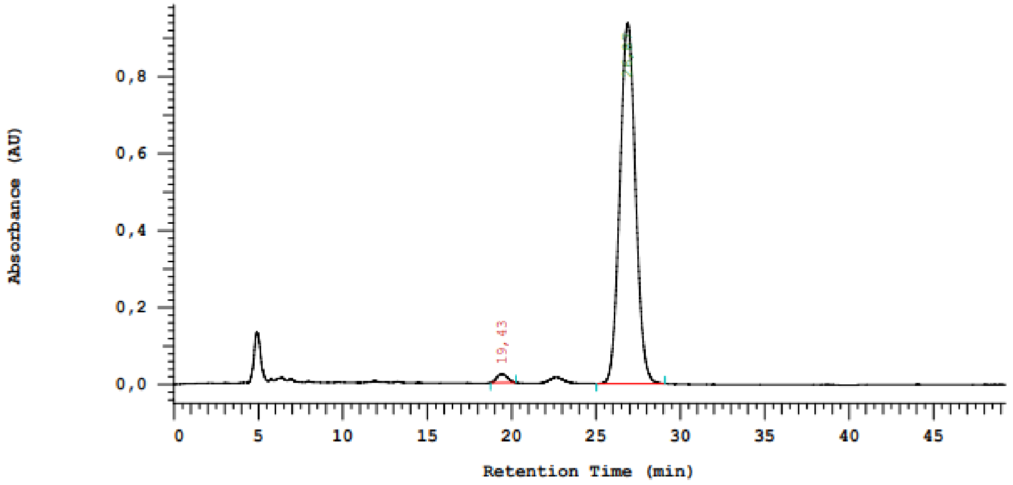

No.	RT	Area	Area %	Name
1	19,43	485695	1,587	
2	26,87	30109904	98,413	enanti (-)
		30595599	100,000	

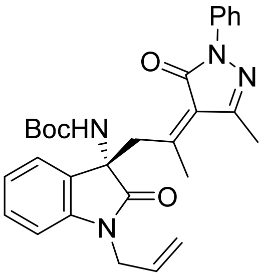

3ca

Sample Name: LC-517 ADH 8020 1mL Vial Number: 1

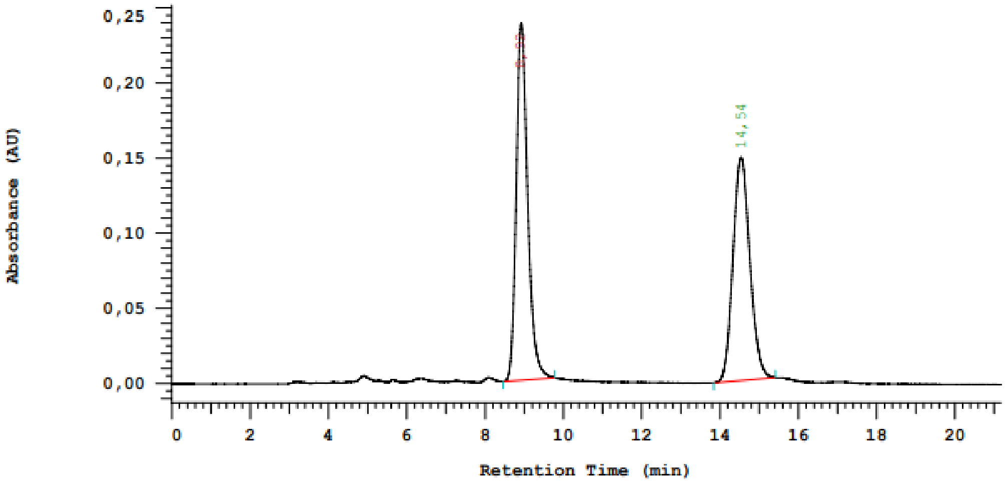

No.	RT	Area	Area %	Name
1	8,92	2399490	51,966	
2	14,54	2217940	48,034	
		4617430	100,000	

Sample Name: LC-526 ADH 8020 1mL Vial Number: 1

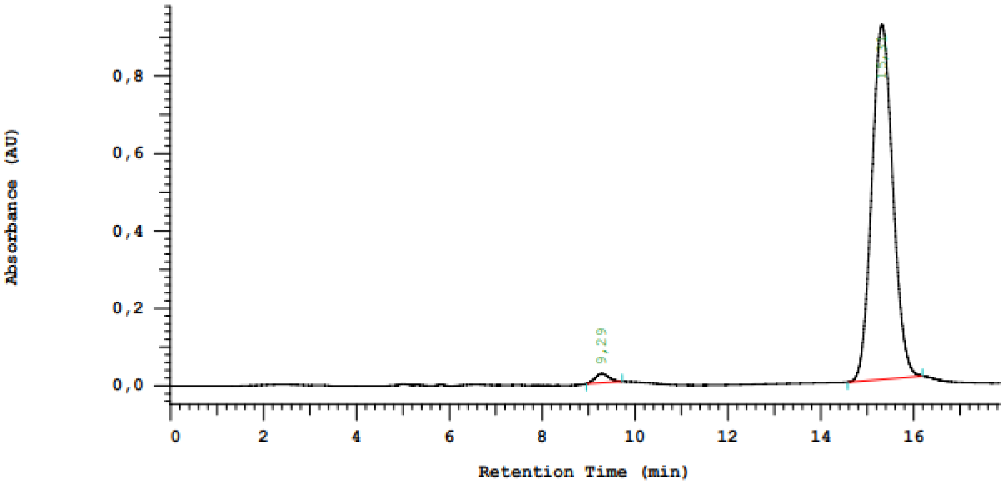

No.	RT	Area	Area %	Name
1	9,29	240335	1,643	
2	15,31	14388380	98,357	
		14628715	100,000	

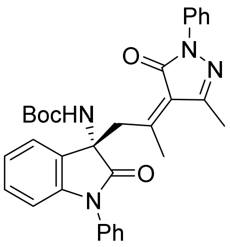

3da

Sample Name: LC-516 ADH 8020 1mL Vial Number: 1

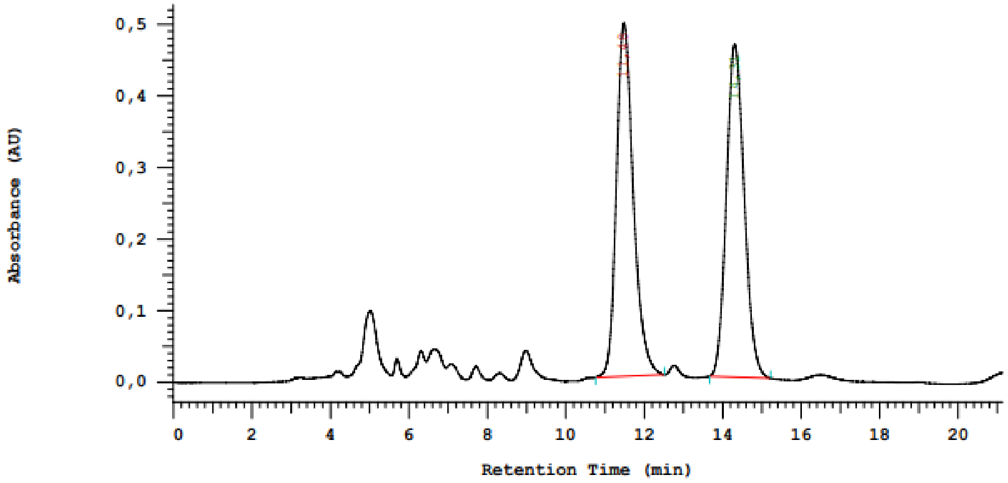

No.	RT	Area	Area %	Name
1	11,48	7402129	50,153	
2	14,31	7357030	49,847	
		14759159	100,000	

Sample Name: LC-530 ADH 8020 1mL Vial Number: 1

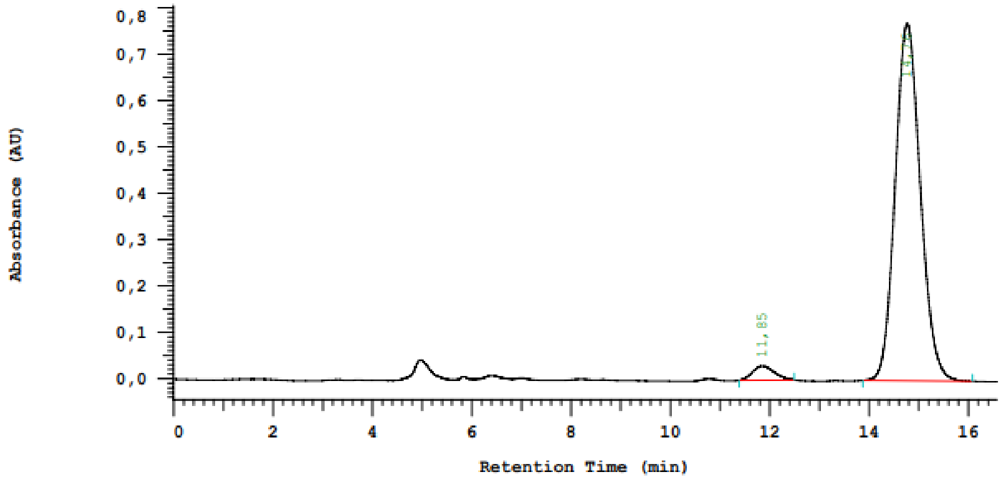

No.	RT	Area	Area %	Name
1	11,85	464950	3,304	
2	14,76	13606694	96,696	
		14071644	100,000	

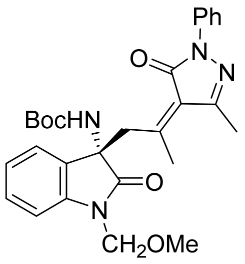

3ea

Sample Name: LC-514 ADH 8020 1mL Vial Number: 1

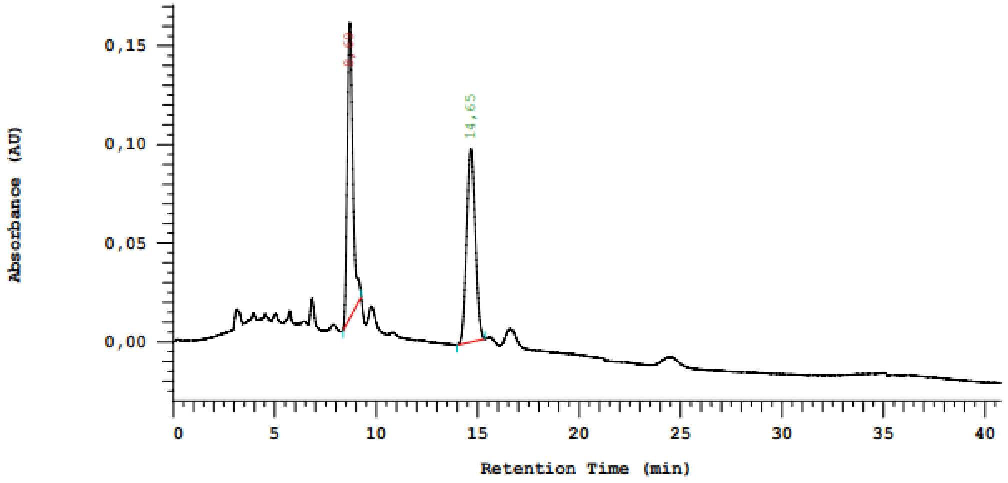

No.	RT	Area	Area %	Name
1	8,69	1395970	49,316	
2	14,65	1434720	50,684	
		2830690	100,000	

Sample Name: LC-525 ADH 8020 1mL Vial Number: 1

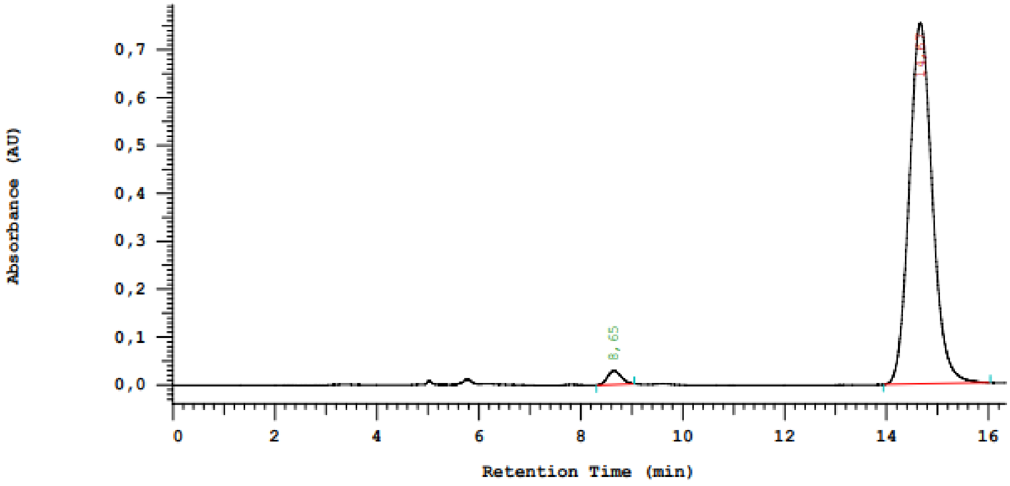

No.	RT	Area	Area %	Name
1	8,65	269470	2,221	
2	14,67	11865780	97,779	
		12135250	100,000	

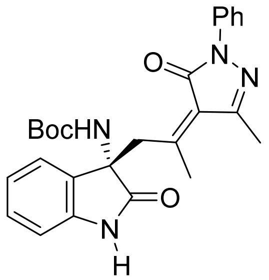

3fa

Sample Name: LC-518 ADH 8020 1mL   Vial Number: 1

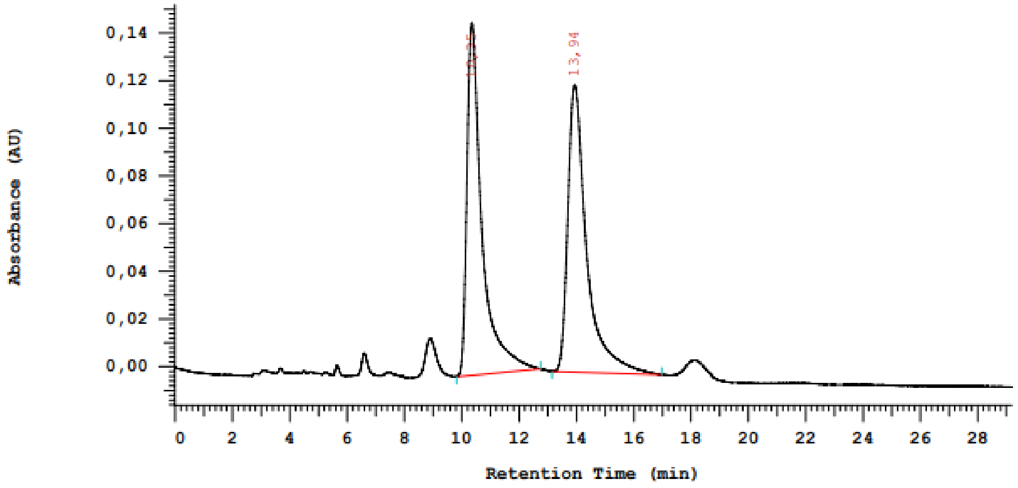

No.	RT	Area	Area %	Name
1	10,35	2677070	49,560	
2	13,94	2724620	50,440	
		5401690	100,000	

Sample Name: LC-523 ADH 8020 1mL   Vial Number: 1

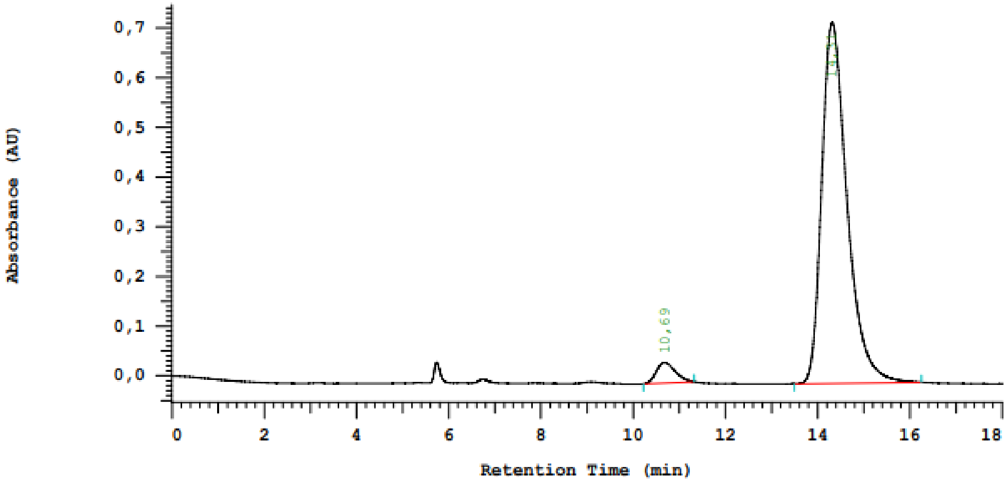

No.	RT	Area	Area %	Name
1	10,69	595080	4,114	
2	14,31	13868070	95,886	
		14463150	100,000	

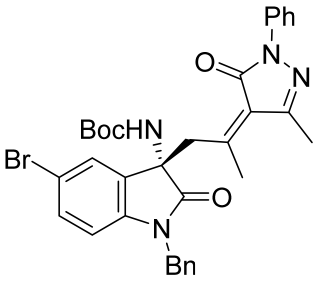

3ga

Sample Name: LC-554B ADH 8020 1mL Vial Number: 1

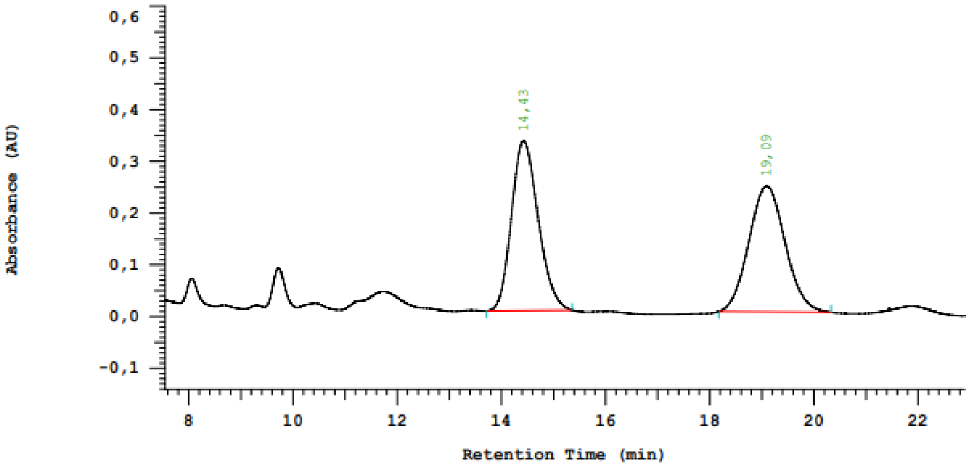

No.	RT	Area	Area %	Name
1	14,43	5851304	49,856	
2	19,09	5885050	50,144	
		11736354	100,000	

Sample Name: LC-864 ADH 8020 1mL Vial Number: 1

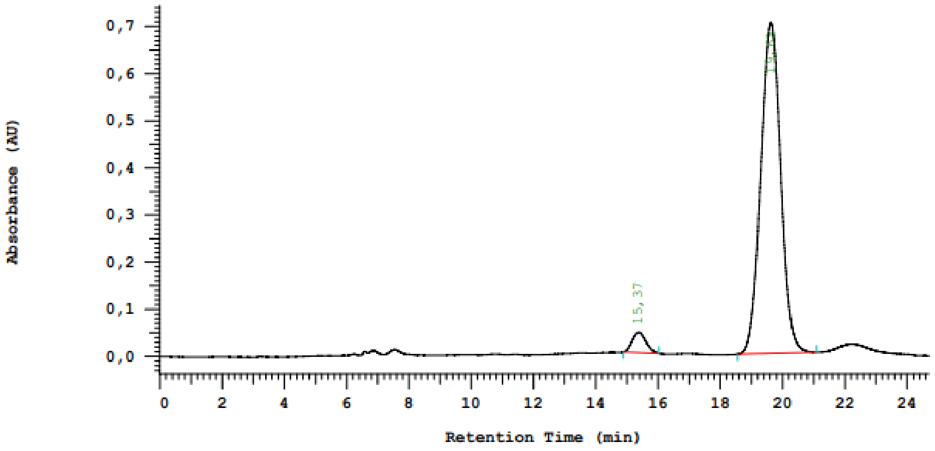

No.	RT	Area	Area %	Name
1	15,37	651870	4,022	
2	19,63	15554150	95,978	
		16206020	100,000	

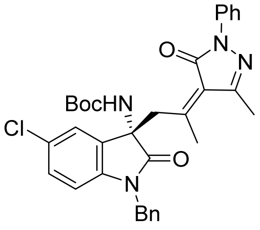

3ha

Sample Name: LC-528 ADH 8020 1mL   Vial Number: 1

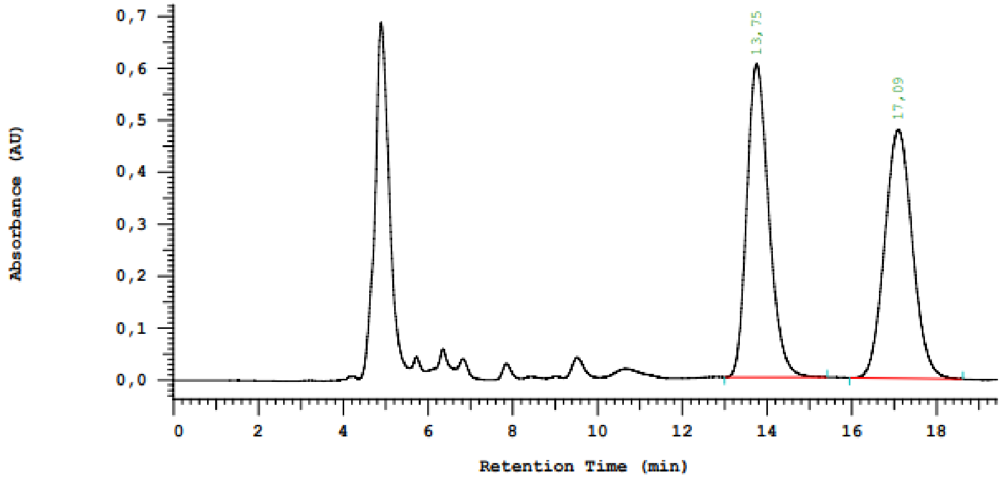

No.	RT	Area	Area %	Name
1	13,75	10871169	49,945	
2	17,09	10895000	50,055	
		21766169	100,000	

Sample Name: LC-532 ADH 8020 1mL   Vial Number: 1

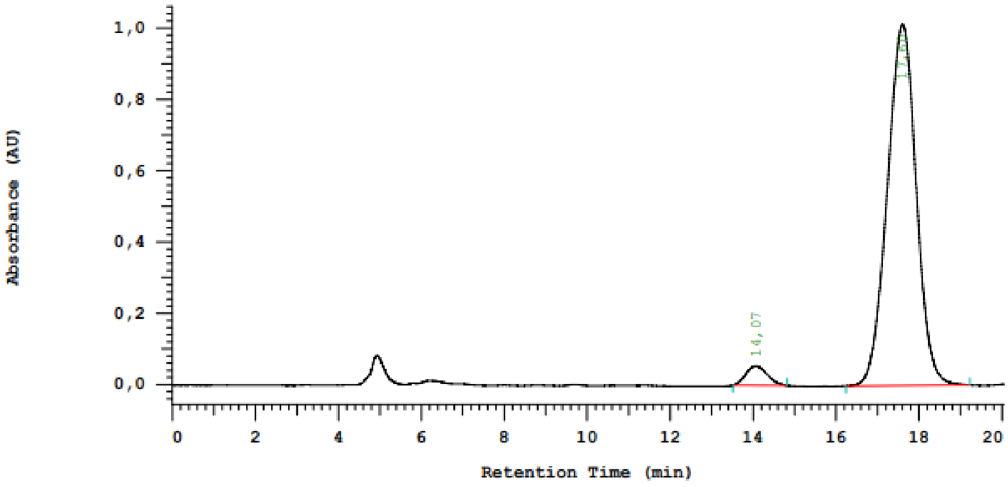

No.	RT	Area	Area %	Name
1	14,07	958010	3,753	
2	17,60	24571035	96,247	
		25529045	100,000	

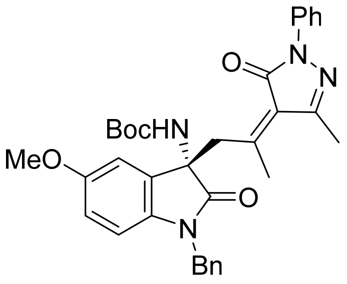

3ia

Sample Name: LC-582 ADH 8020 1mL Vial Number: 1

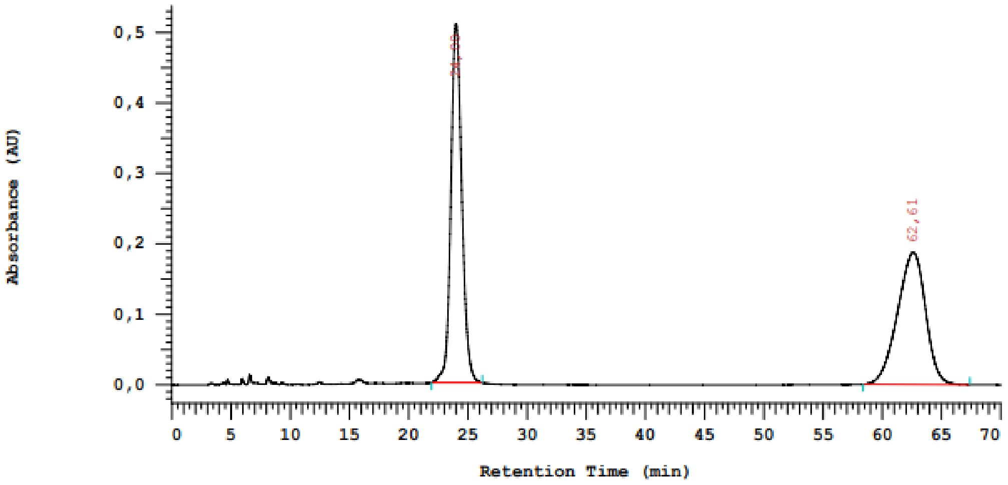

No.	RT	Area	Area %	Name
1	24,00	16113720	51,073	enant. (+)
2	62,61	15436390	48,927	
		31550110	100,000	

Sample Name: LC-573 ADH 8020 1mL Vial Number: 1

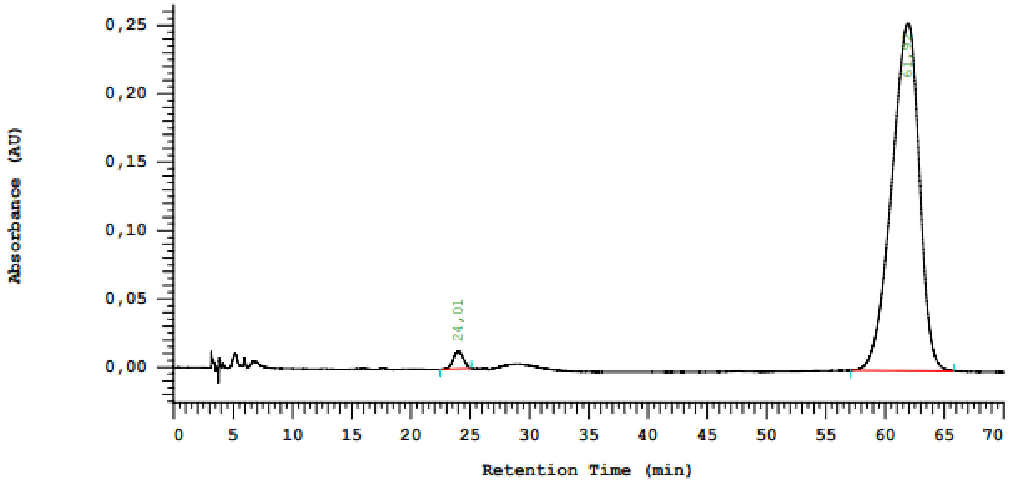

No.	RT	Area	Area %	Name
1	24,01	377275	1,807	enant. (+)
2	61,92	20505284	98,193	
		20882559	100,000	

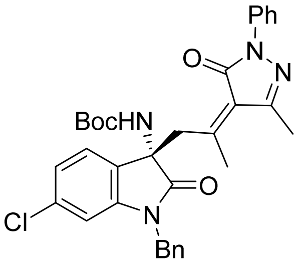

3ja

Sample Name: LC-527 iAmilose1 8020 1mL Vial Number: 1

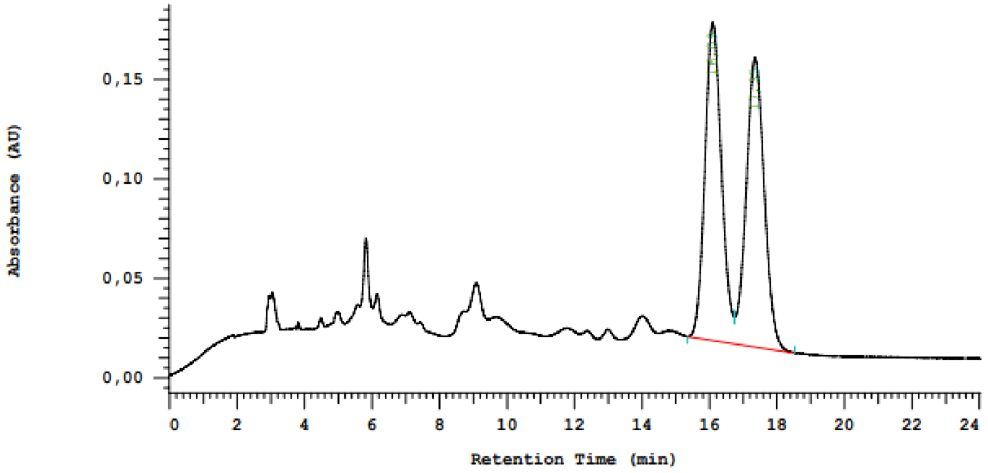

No.	RT	Area	Area %	Name
1	16,09	2736196	50,127	
2	17,35	2722343	49,873	
		5458539	100,000	

Sample Name: LC-531 iAmilose1 8020 1mL Vial Number: 1

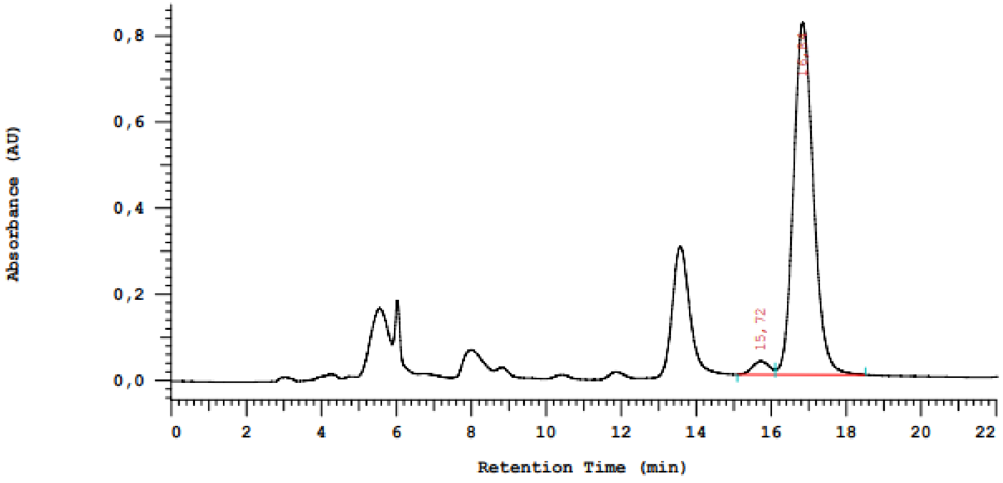

No.	RT	Area	Area %	Name
1	15,72	476692	3,070	
2	16,84	15049187	96,930	
		15525879	100,000	

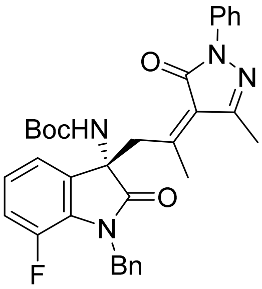

3ka

Sample Name: LC-535 ADH 8020 1mL   Vial Number: 1

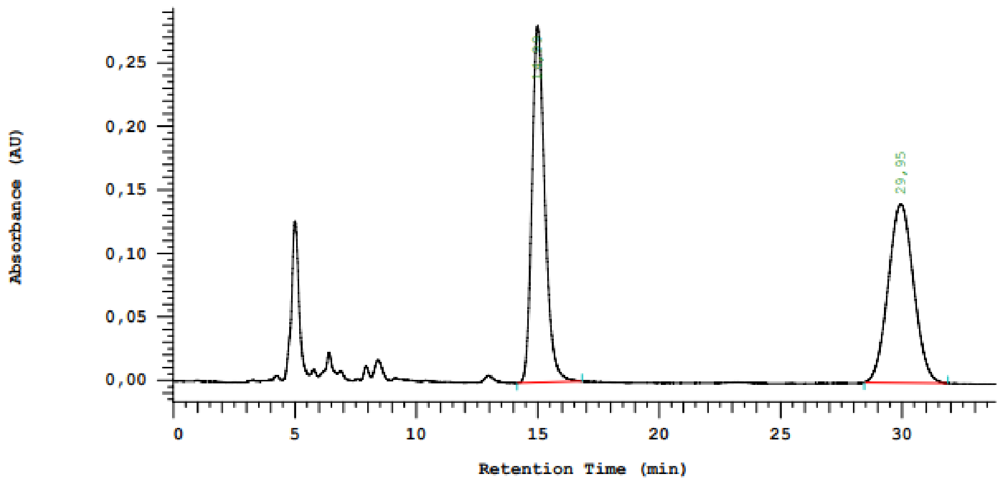

No.	RT	Area	Area %	Name
1	14,99	5190530	50,342	
2	29,95	5119915	49,658	enanti (-)
		10310445	100,000	

Sample Name: LC-542 ADH 8020 1mL   Vial Number: 1

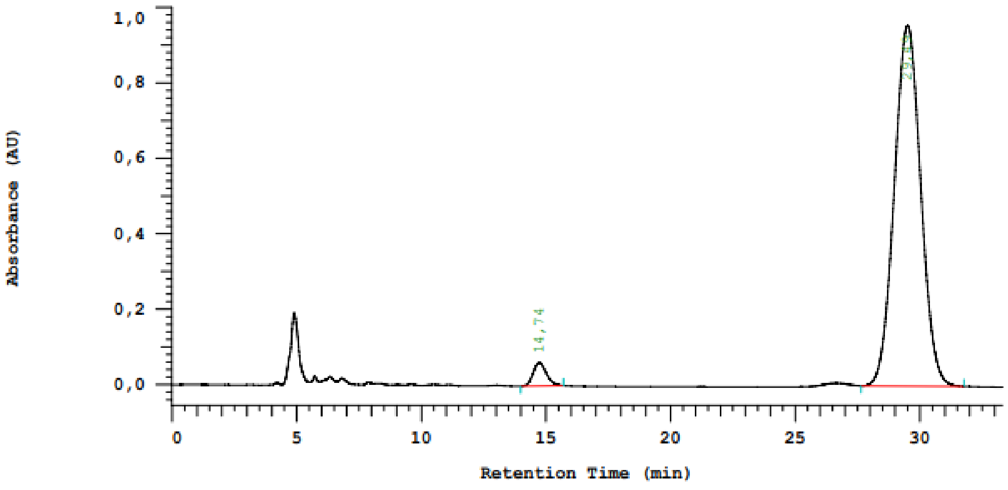

No.	RT	Area	Area %	Name
1	14,74	1135160	3,123	
2	29,49	35218684	96,877	enanti (-)
		36353844	100,000	

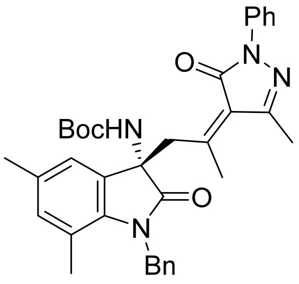

3la

Sample Name: LC-529 iAmilose1 8020 1mL Vial Number: 1

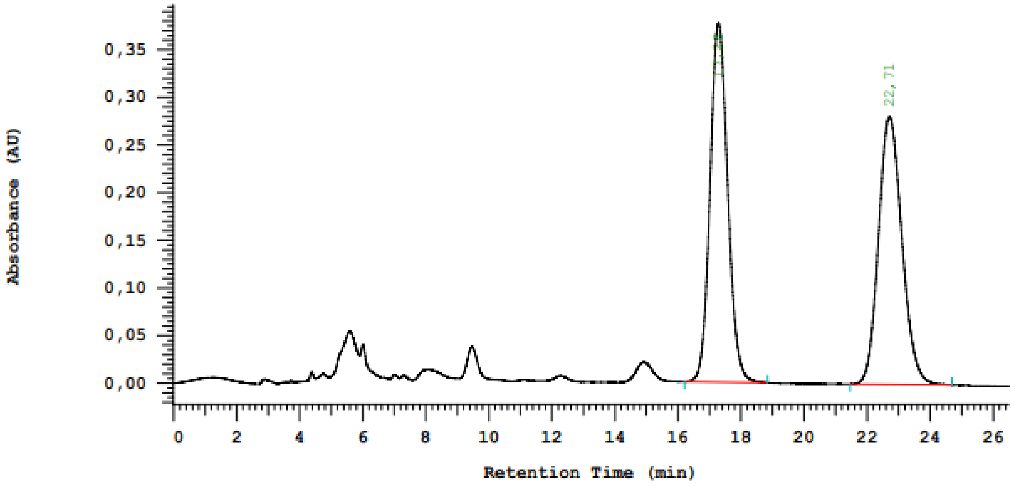

No.	RT	Area	Area %	Name
1	17,28	7342790	50,399	
2	22,71	7226449	49,601	enant. (+)
		14569239	100,000	

Sample Name: LC-533 iAmilose1 8020 1mL Vial Number: 1

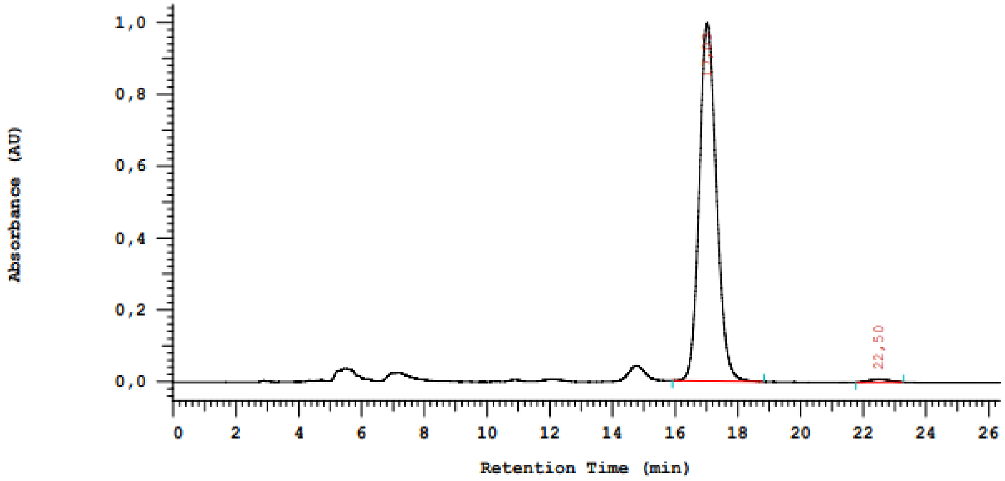

No.	RT	Area	Area %	Name
1	17,03	18999049	99,013	
2	22,50	189430	0,987	enant. (+)
		19188479	100,000	

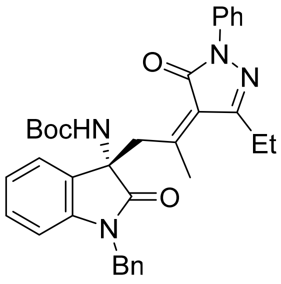

3bb

Sample Name: LC-537 ADH 8020 1mL   Vial Number: 1

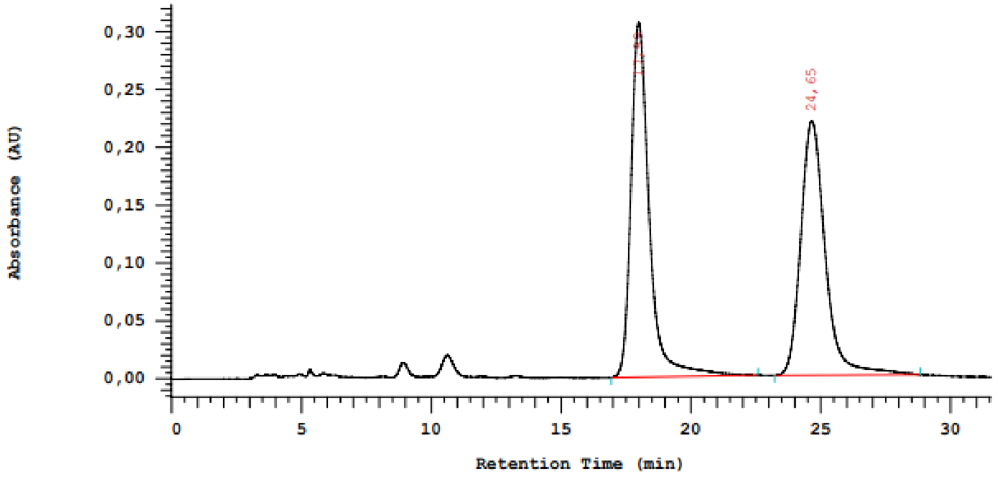

No.	RT	Area	Area %	Name
1	17,99	7475150	50,968	
2	24,65	7191240	49,032	enant. (+)
		14666390	100,000	

Sample Name: LC-549 ADH 8020 1mL   Vial Number: 1

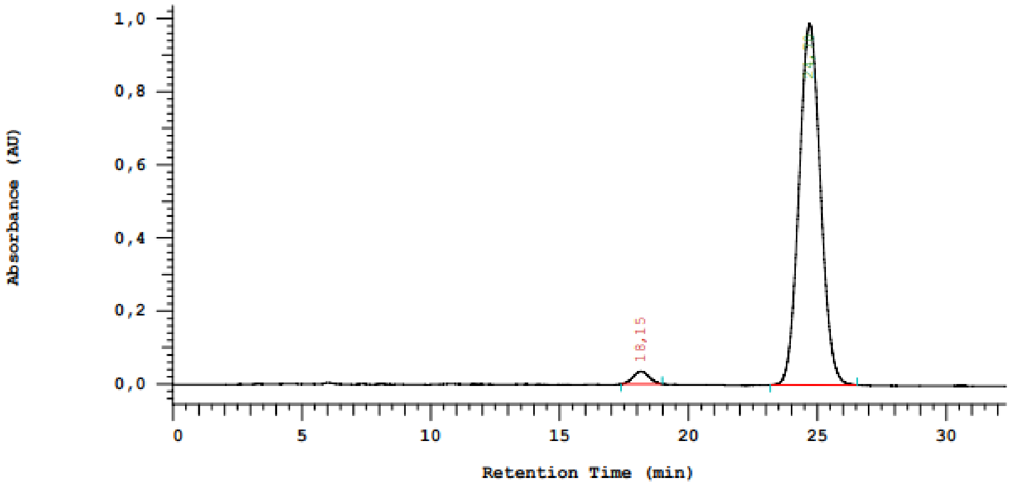

No.	RT	Area	Area %	Name
1	18,15	750780	2,573	
2	24,70	28427440	97,427	enant. (+)
		29178220	100,000	

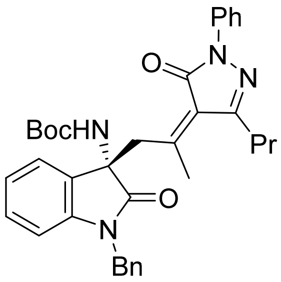

Sample Name: LC-540 iAmilose1 8020 1mL   Vial Number: 1

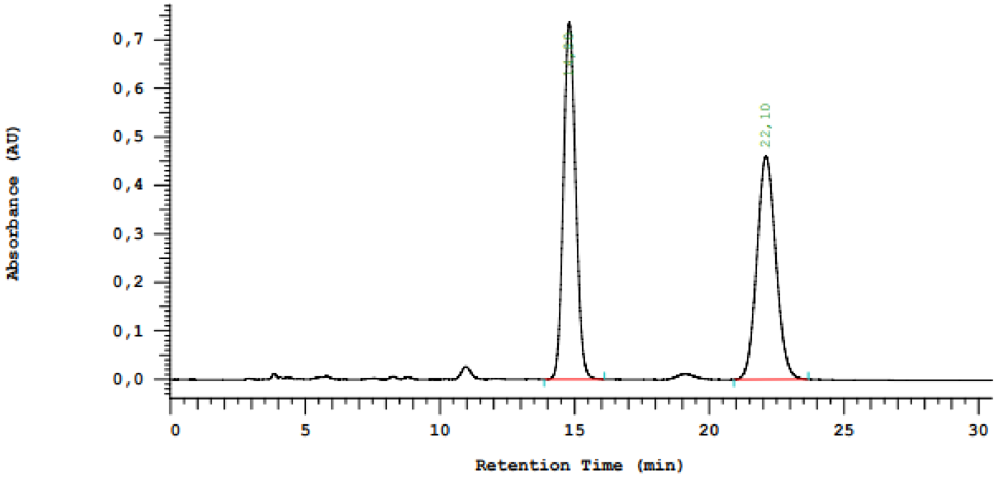

No.	RT	Area	Area %	Name
1	14,80	11480555	50,474	
2	22,10	11265150	49,526	enant. (+)
		22745705	100,000	

Sample Name: LC-550 iAmilose1 8020 1mL   Vial Number: 1

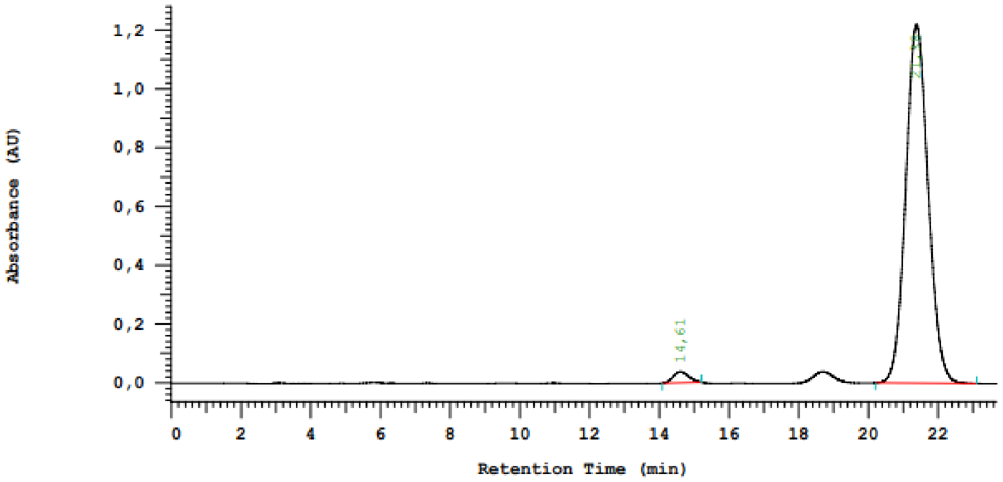

No.	RT	Area	Area %	Name
1	14,61	578635	2,079	
2	21,38	27260374	97,921	
		27839009	100,000	

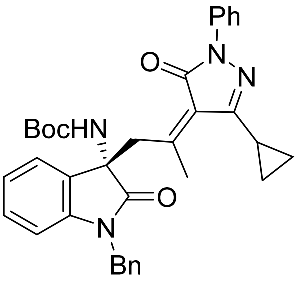

3bd

Sample Name: LC-543 ADH 8020 1mL Vial Number: 1

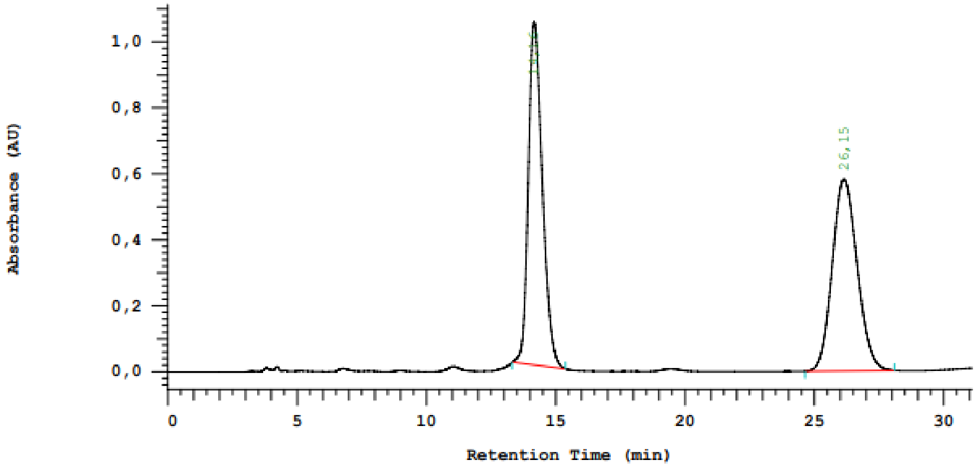

No.	RT	Area	Area %	Name
1	14,16	19452720	50,057	
2	26,15	19408049	49,943	enant. (+)
		38860769	100,000	

Sample Name: LC-549 ADH 8020 1mL Vial Number: 1

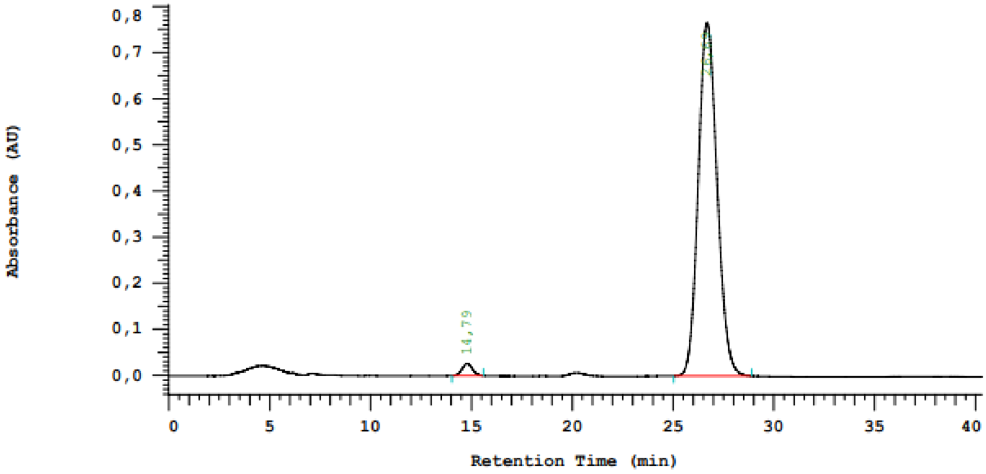

No.	RT	Area	Area %	Name
1	14,79	455950	1,806	
2	26,69	24786310	98,194	enanti (-)
		25242260	100,000	

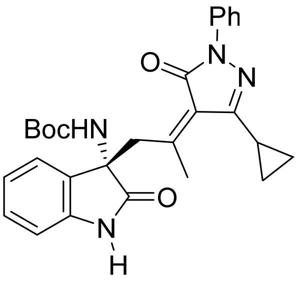

3fd

Sample Name: LC-589 ADH 8020 1mL   Vial Number: 1

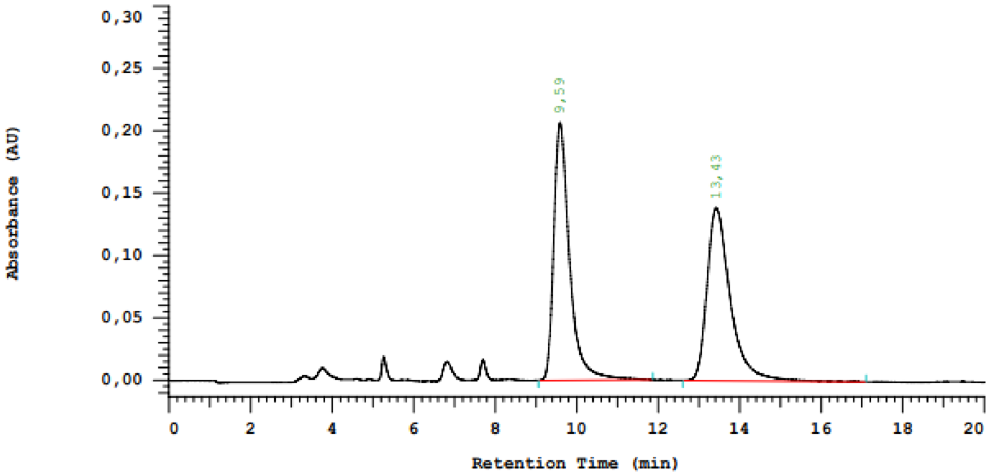

No.	RT	Area	Area %	Name
1	9,59	2782180	50,099	
2	13,43	2771225	49,901	
		5553405	100,000	

Sample Name: LC-588 ADH 8020 1mL   Vial Number: 1

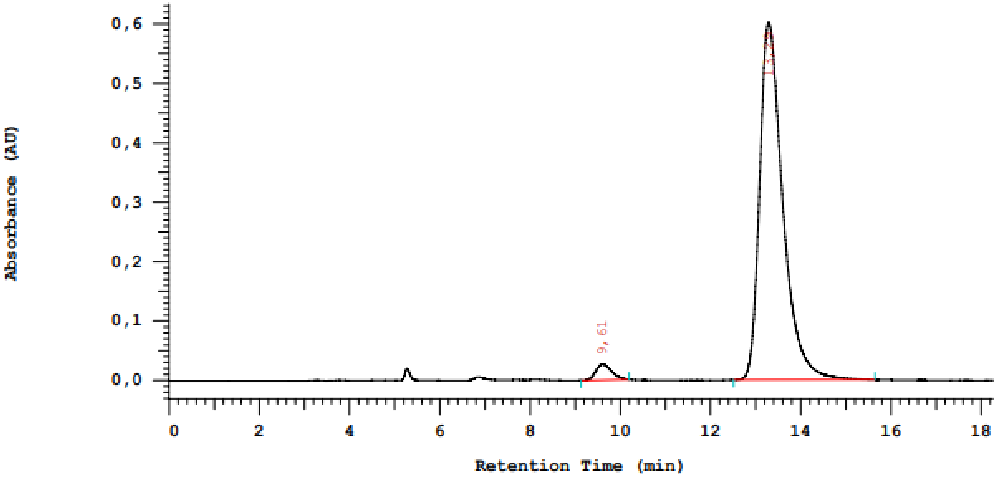

No.	RT	Area	Area %	Name
1	9,61	320410	2,891	
2	13,29	10761670	97,109	
		11082080	100,000	

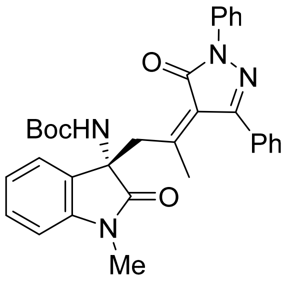

3ae

Sample Name: LC-565+558 ADH 8020 1mL   Vial Number: 1

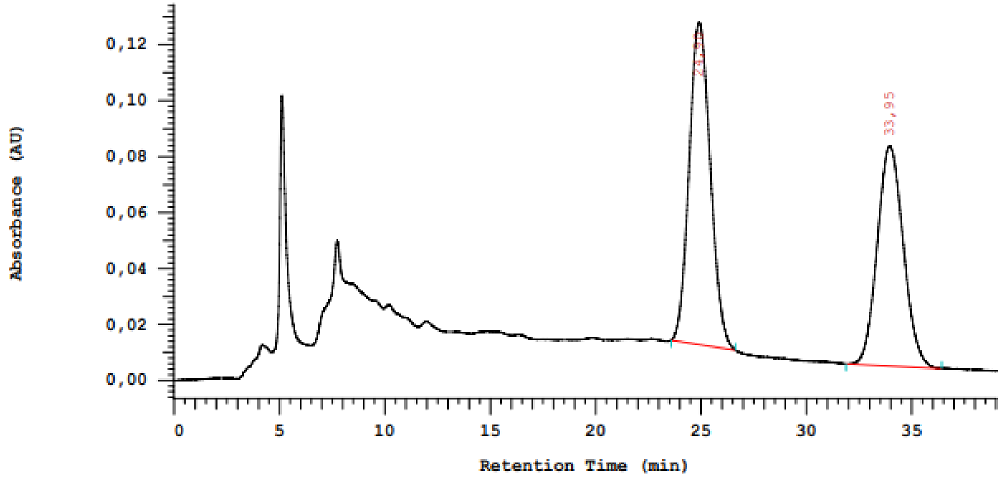

No.	RT	Area	Area %	Name
1	24,90	3996060	54,230	enant. (+)
2	33,95	3372650	45,770	
		7368710	100,000	

Sample Name: LC-558 ADH 8020 1mL   Vial Number: 1

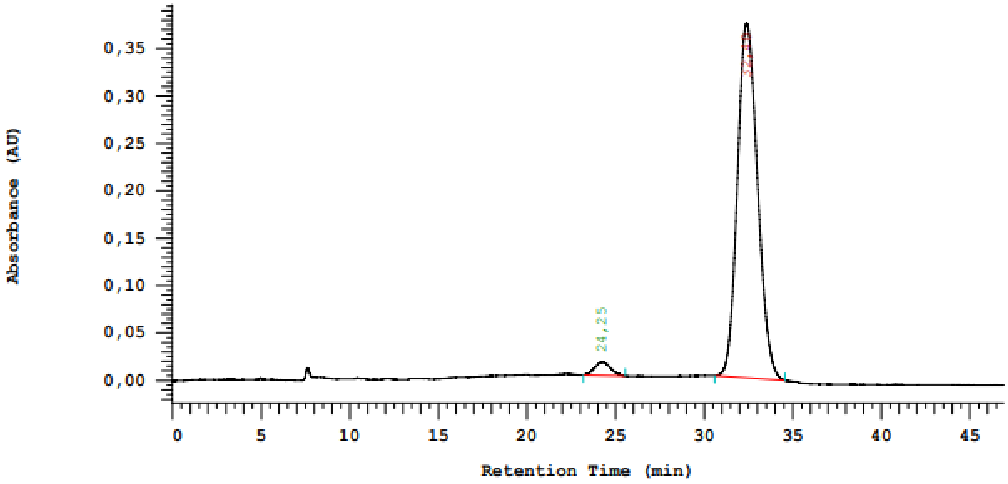

No.	RT	Area	Area %	Name
1	24,25	431480	2,880	enant. (+)
2	32,40	14548060	97,120	
		14979540	100,000	

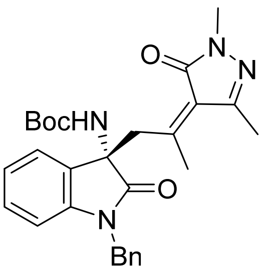

3bf

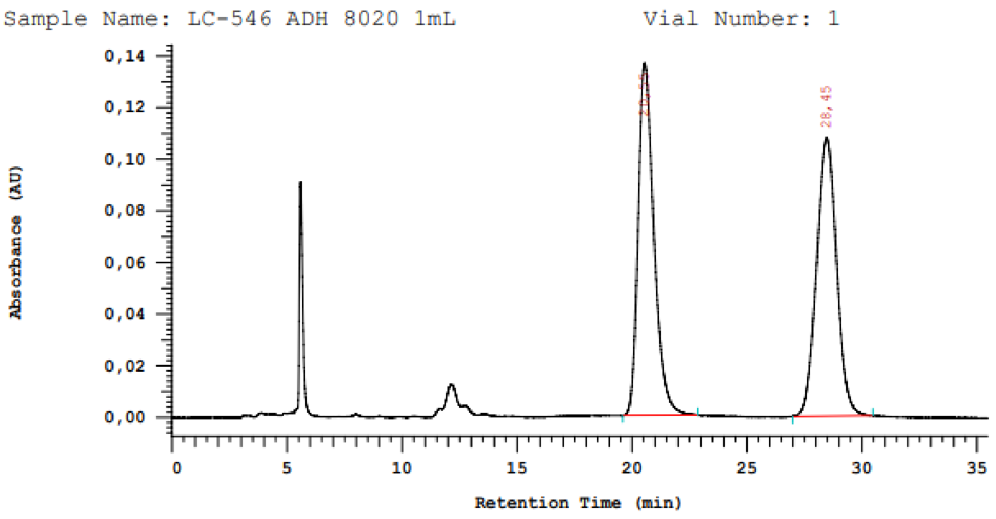

No.	RT	Area	Area %	Name
1	20,55	3262305	50,105	
2	28,45	3248690	49,895	enanti (-)
		6510995	100,000	

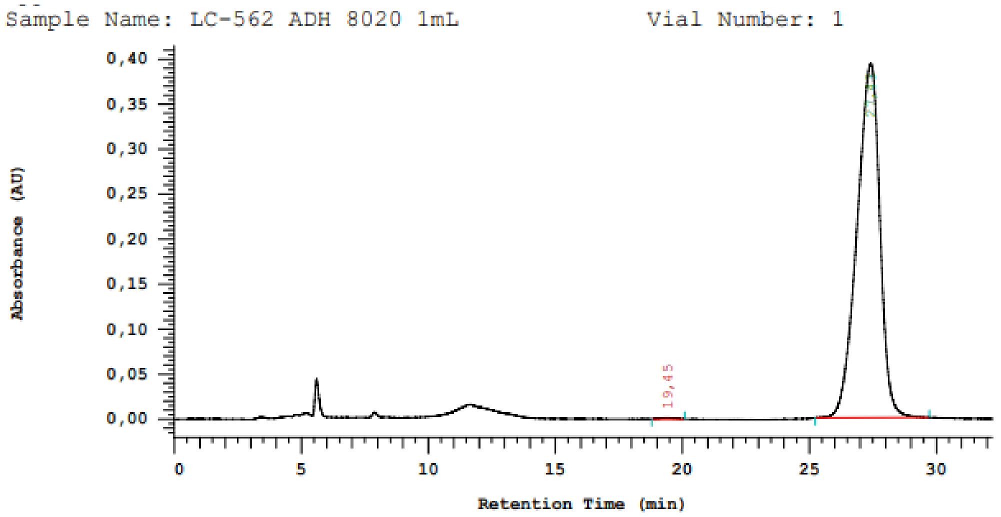

No.	RT	Area	Area %	Name
1	19,45	30400	0,246	
2	27,43	12303920	99,754	enanti (-)
		12334320	100,000	

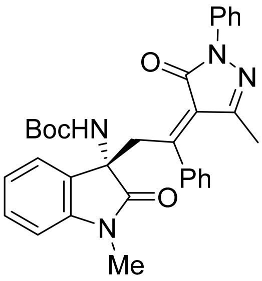

3ag

Sample Name: LC-570+567 ADH 8020 1mL Vial Number: 1

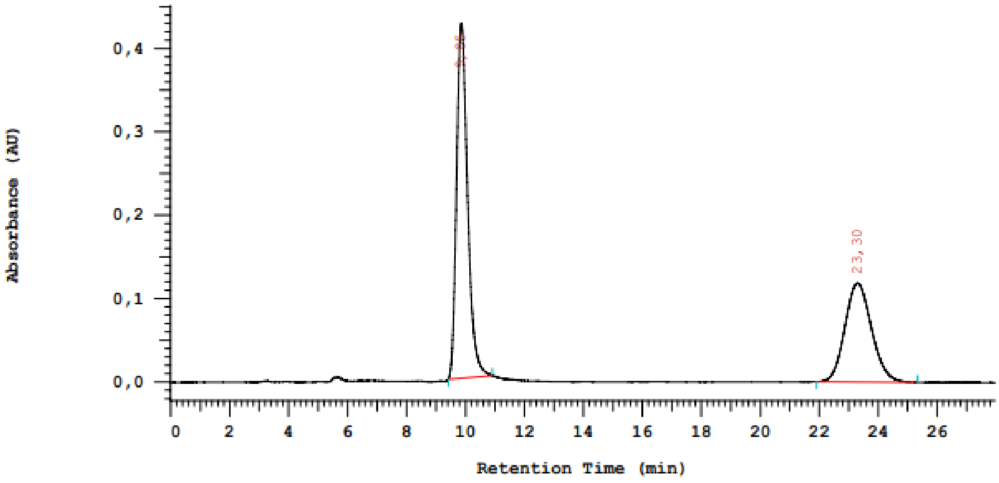

No.	RT	Area	Area %	Name
1	9,85	5447224	59,434	
2	23,30	3717970	40,566	enant. (+)
		9165194	100,000	

Sample Name: LC-570 ADH 8020 1mL Vial Number: 1

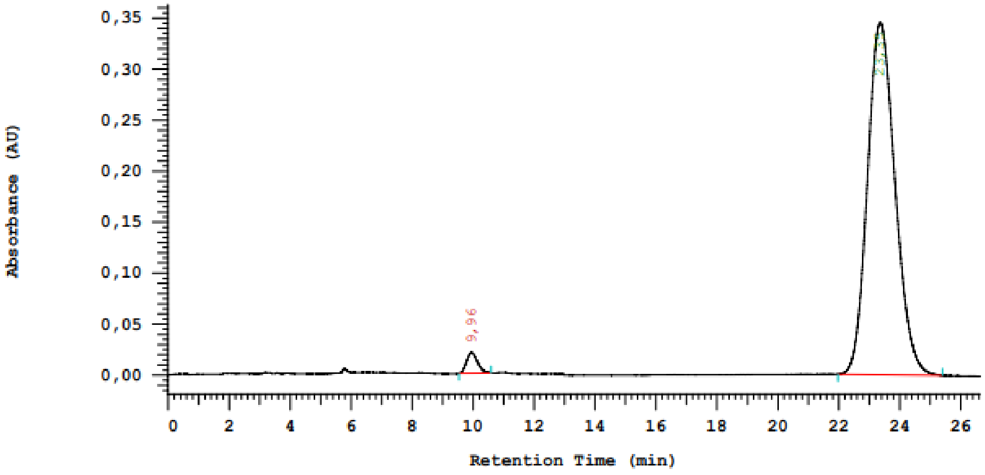

No.	RT	Area	Area %	Name
1	9,96	249130	2,242	
2	23,35	10862220	97,758	enant. (+)
		11111350	100,000	

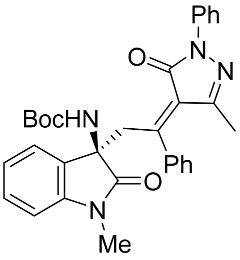

ent-**3ag**,

Sample Name: LC-567 ADH 8020 1mL Vial Number: 1

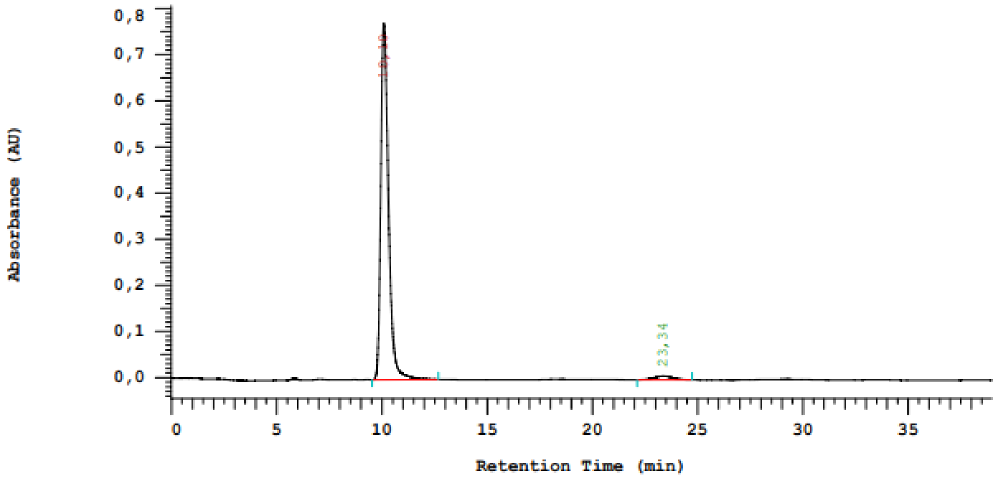

No.	RT	Area	Area %	Name
1	10,10	9697849	97,558	
2	23,34	242750	2,442	enant. (+)
		9940599	100,000	

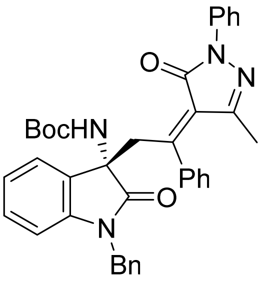

3bg

Sample Name: LC-547 ADH 8020 1mL Vial Number: 1

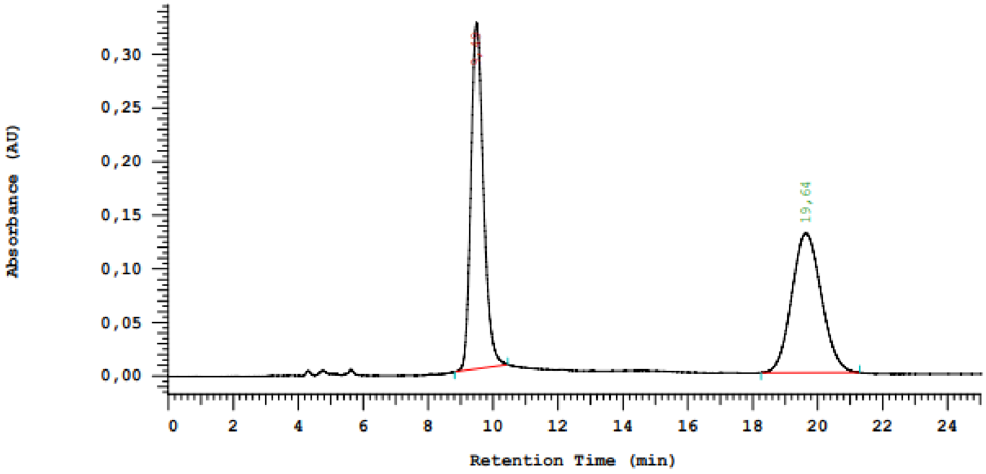

No.	RT	Area	Area %	Name
1	9,49	4318835	51,121	
2	19,64	4129410	48,879	
		8448245	100,000	

Sample Name: LC-555 ADH 8020 1mL Vial Number: 1

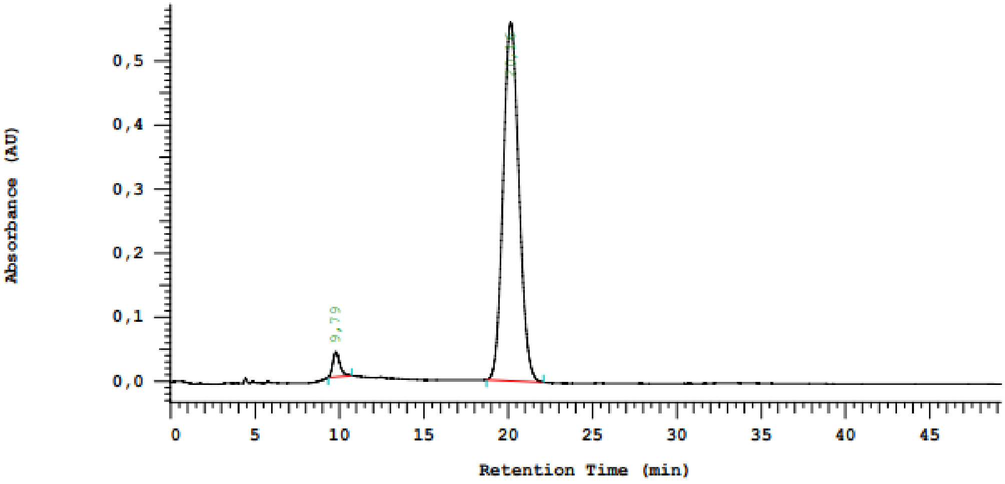

No.	RT	Area	Area %	Name
1	9,79	560610	3,017	
2	20,16	18022760	96,983	
		18583370	100,000	

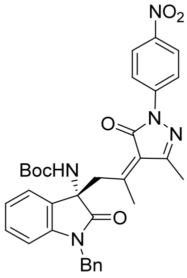

3bh

Sample Name: LC-544 IC 8020 1mL   Vial Number: 1

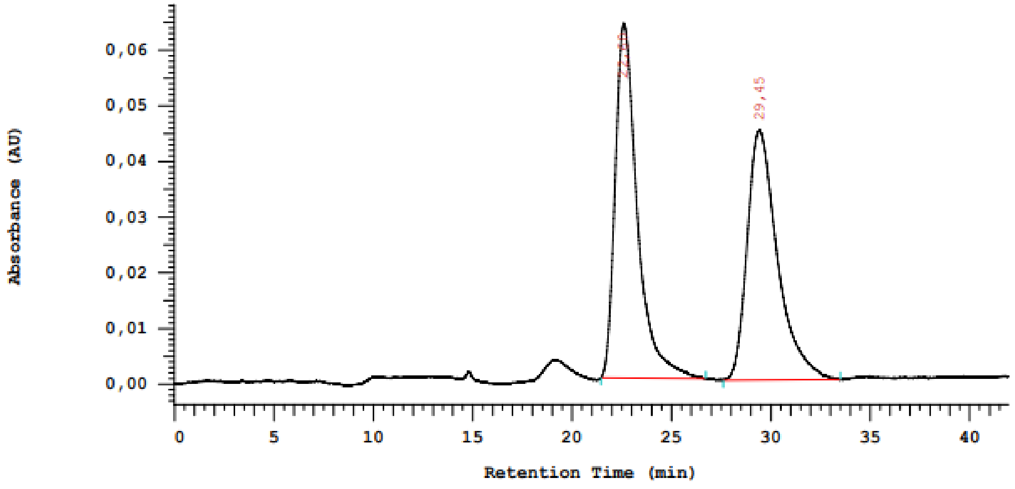

No.	RT	Area	Area %	Name
1	22,60	2453025	50,909	enant. (+)
2	29,45	2365420	49,091	enanti (-)
		4818445	100,000	

Sample Name: LC-559 IC 8020 1mL   Vial Number: 1

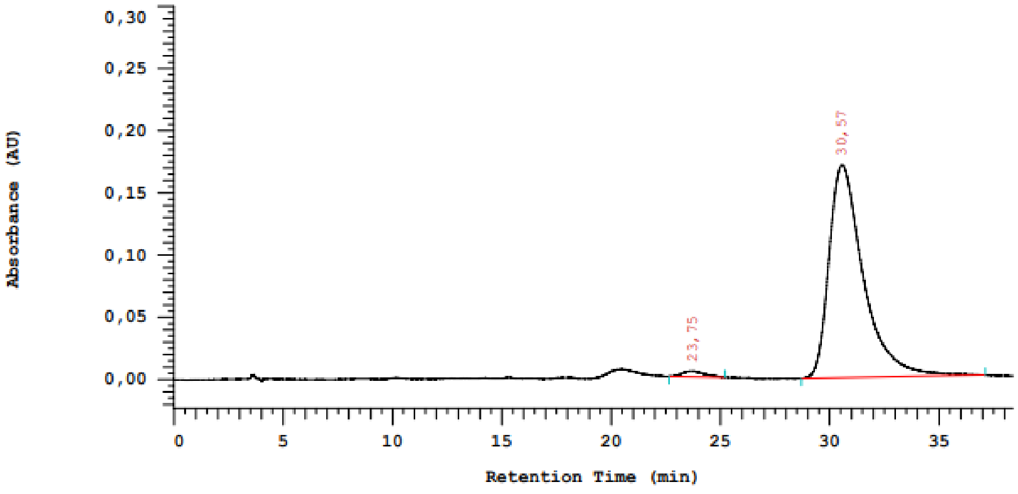

No.	RT	Area	Area %	Name
1	23,75	165155	1,741	enant. (+)
2	30,57	9320000	98,259	enanti (-)
		9485155	100,000	

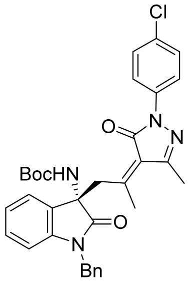

3bi

Sample Name: LC-539 iAmilose1 8020 1mL Vial Number: 1

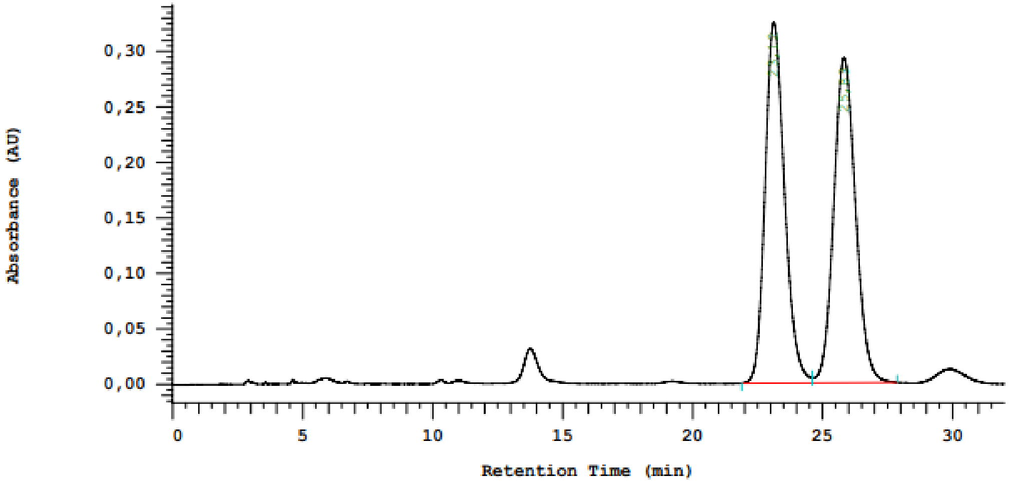

No.	RT	Area	Area %	Name
1	23,12	8479282	49,912	enant. (+)
2	25,83	8509057	50,088	enanti (-)
		16988339	100,000	

Sample Name: LC-556 iAmilose1 8020 1mL Vial Number: 1

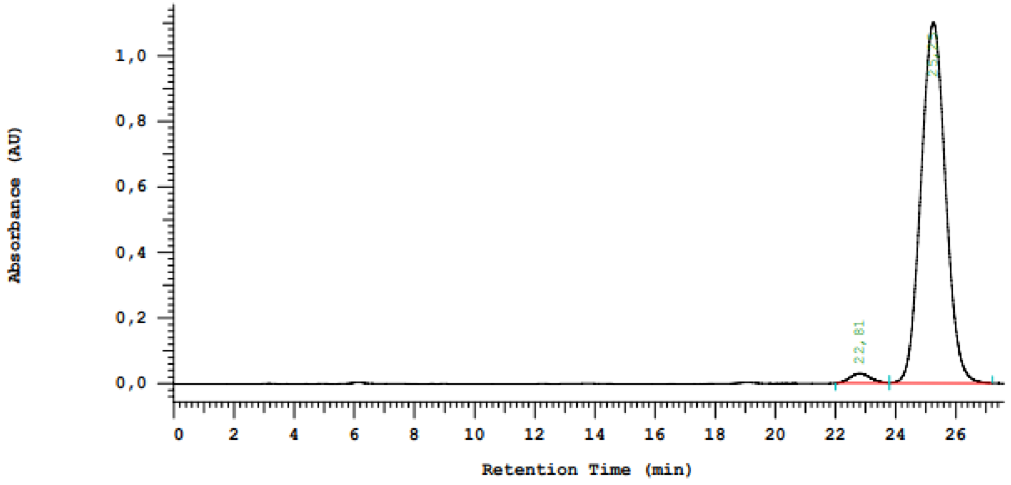

No.	RT	Area	Area %	Name
1	22,81	693954	2,178	enant. (+)
2	25,25	31160924	97,822	
		31854878	100,000	

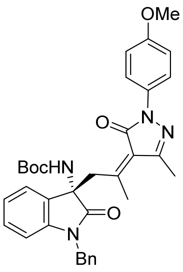

3bj

Sample Name: LC-538 ODH 8020 1mL Vial Number: 1

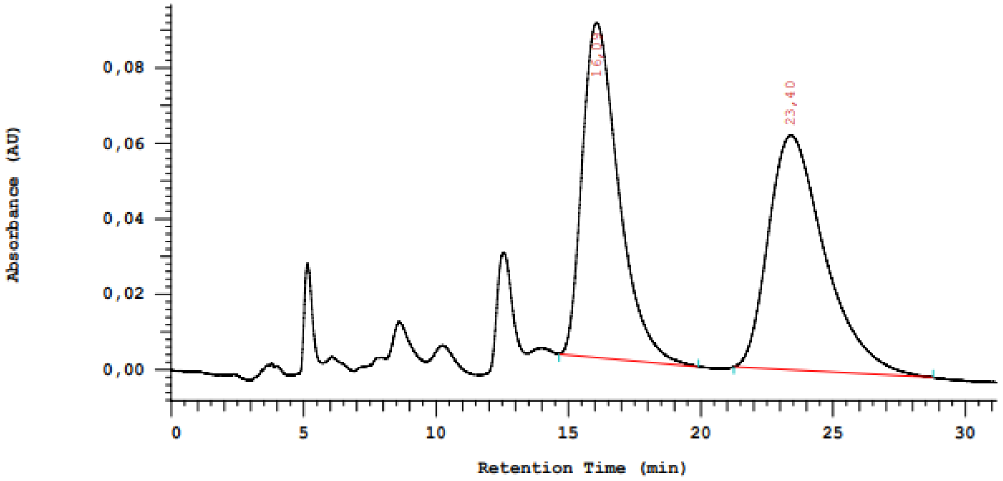

No.	RT	Area	Area %	Name
1	16,09	4242880	48,020	
2	23,40	4592840	51,980	enant. (+)
		8835720	100,000	

Sample Name: LC-557 ODH 8020 1mL Vial Number: 1

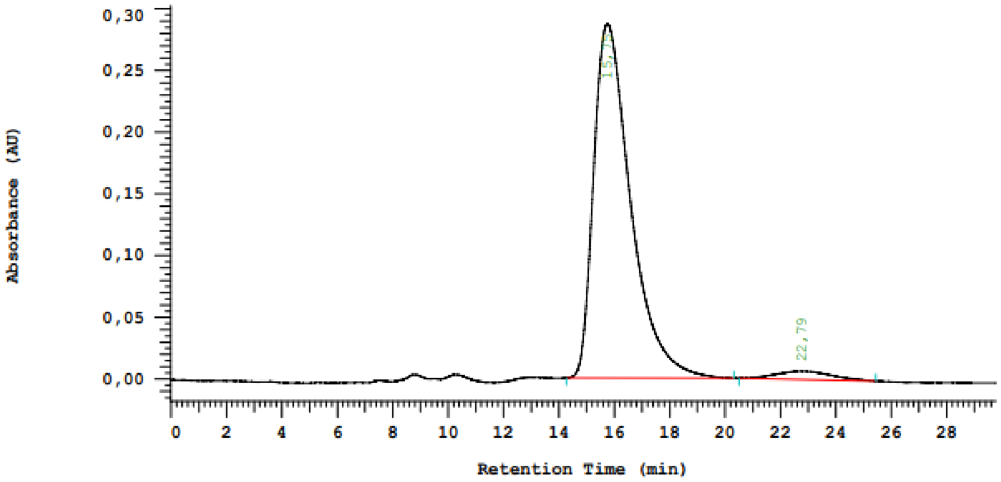

No.	RT	Area	Area %	Name
1	15,75	13295035	96,508	
2	22,79	481080	3,492	enant. (+)
		13776115	100,000	

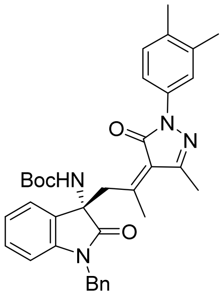

3bk

Sample Name: LC-545 ADH 8020 1mL Vial Number: 1

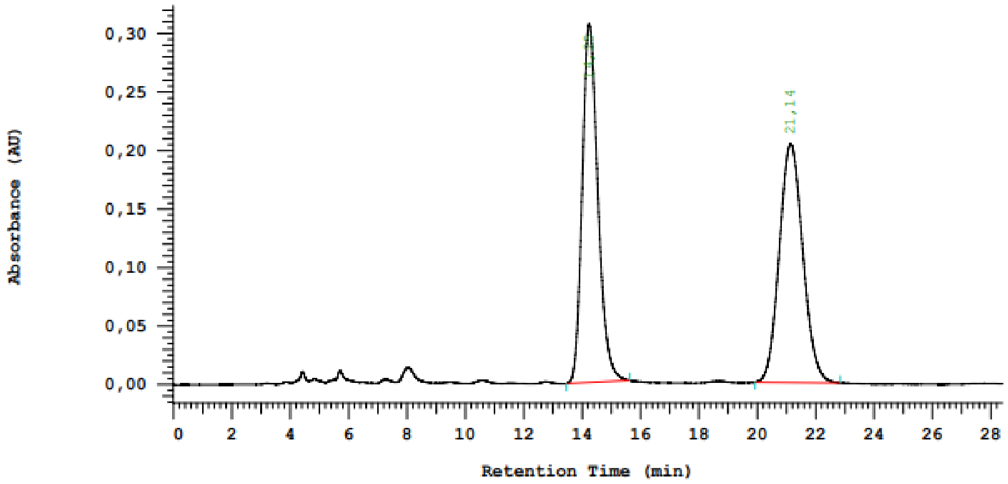

No.	RT	Area	Area %	Name
1	14,25	5715480	50,104	
2	21,14	5691800	49,896	
		11407280	100,000	

Sample Name: LC-560 ADH 8020 1mL Vial Number: 1

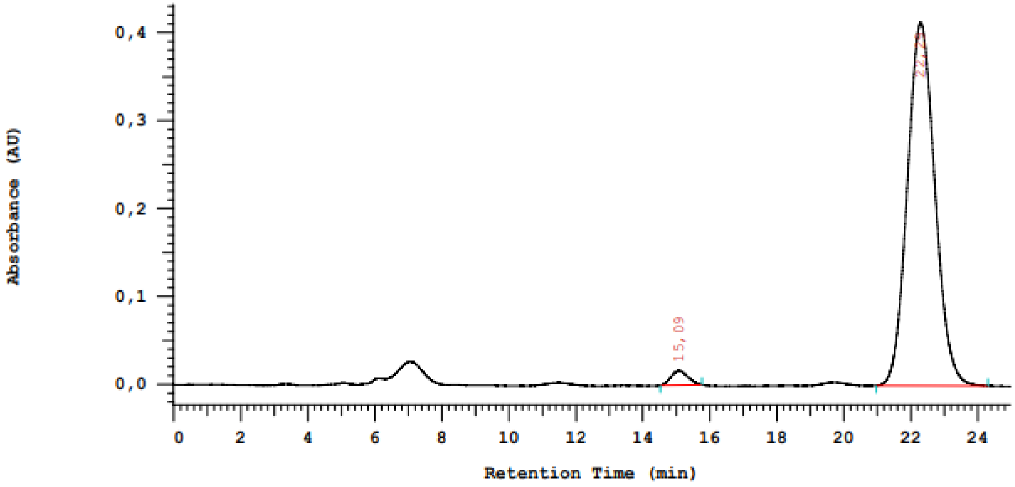

No.	RT	Area	Area %	Name
1	15,09	287520	2,380	
2	22,29	11795300	97,620	enant. (+)
		12082820	100,000	

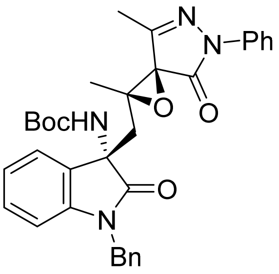

4 (major diastereoisomer)

Sample Name: LC-583A ADH 8020 1mL Vial Number: 1

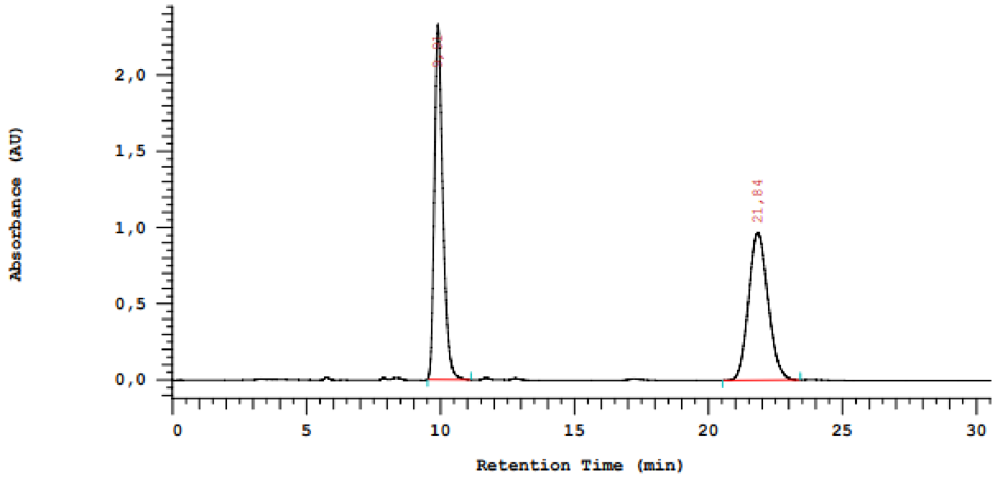

No.	RT	Area	Area %	Name
1	9,91	25336160	50,940	
2	21,84	24401230	49,060	enant. (+)
		49737390	100,000	

Sample Name: LC-579A ADH 8020 1mL Vial Number: 1

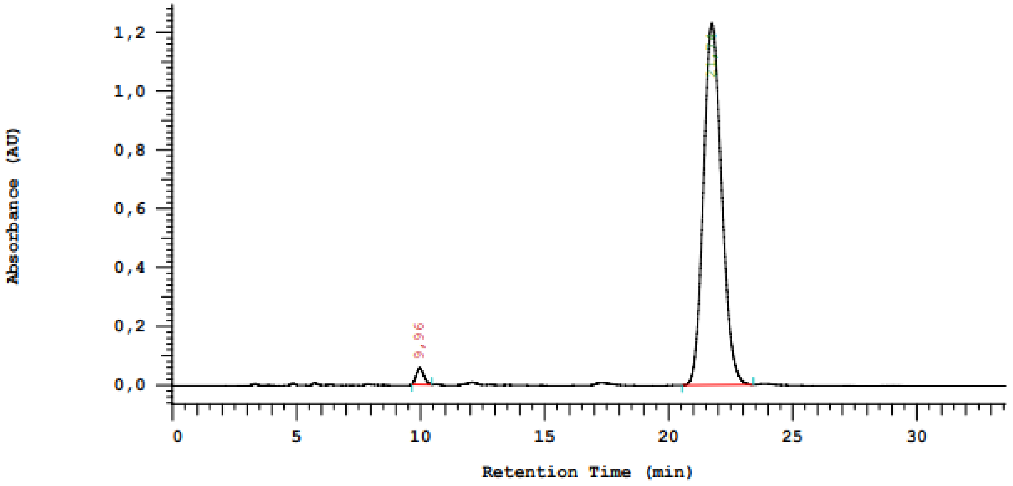

No.	RT	Area	Area %	Name
1	9,96	578610	1,830	
2	21,74	31031814	98,170	enant. (+)
		31610424	100,000	

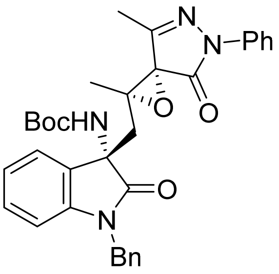

4 (minor diastereoisomer)

Sample Name: LC-589 ADH 8020 1mL Vial Number: 1

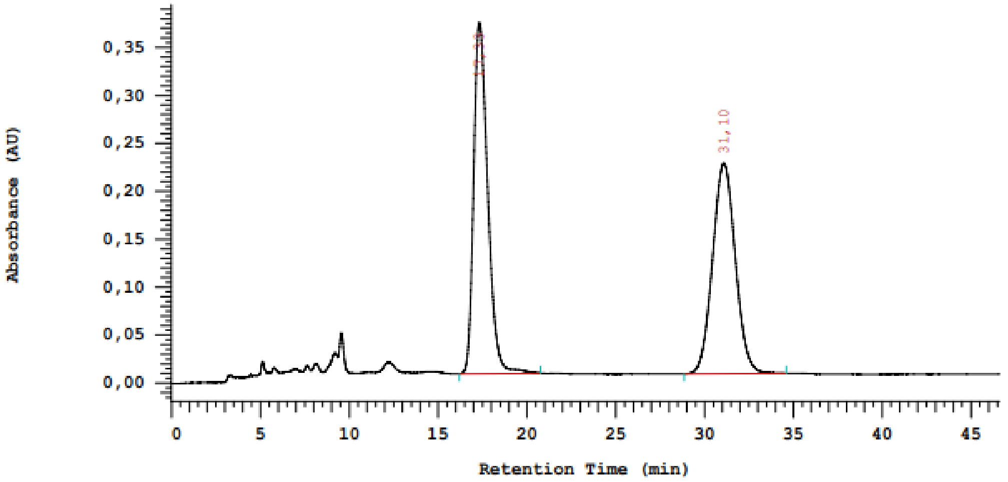

No.	RT	Area	Area %	Name
1	17,33	9972500	50,529	
2	31,10	9763564	49,471	
		19736064	100,000	

Sample Name: LC-589 ADH 8020 1mL Vial Number: 1

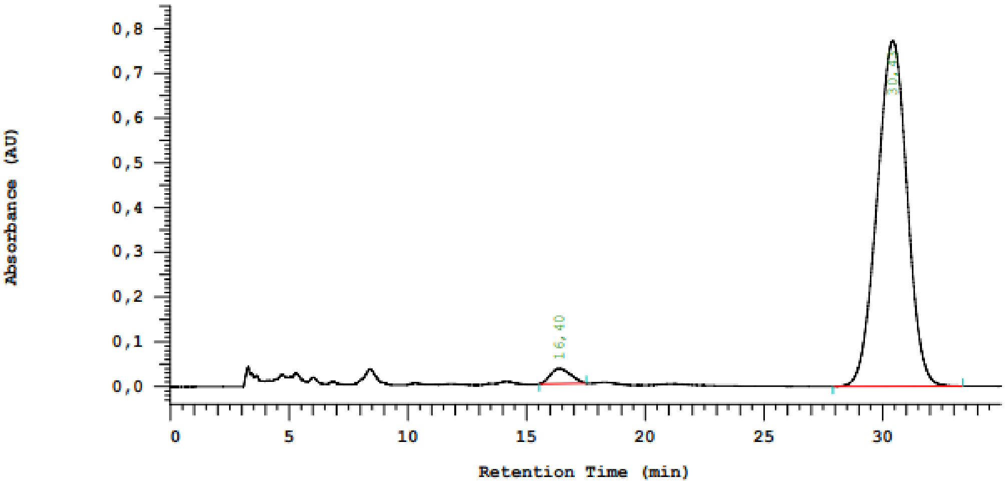

No.	RT	Area	Area %	Name
1	16,40	987430	2,836	
2	30,43	33829980	97,164	enanti (-)
		34817410	100,000	

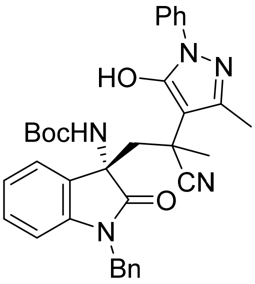

5

Sample Name: LC-586 IC 8020 1mL Vial Number: 1

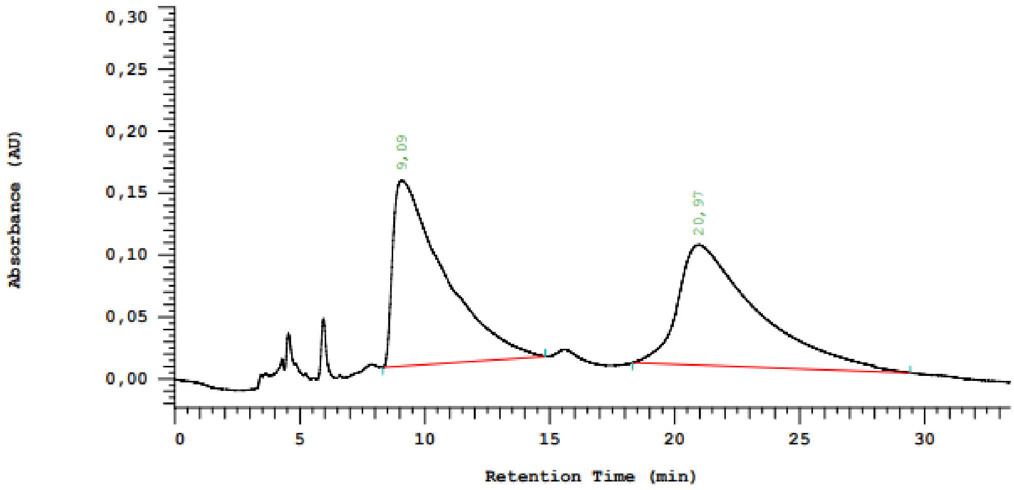

No.	RT	Area	Area %	Name
1	9,09	10673270	50,030	
2	20,97	10660655	49,970	
		21333925	100,000	

Sample Name: LC-582 IC 8020 1mL Vial Number: 1

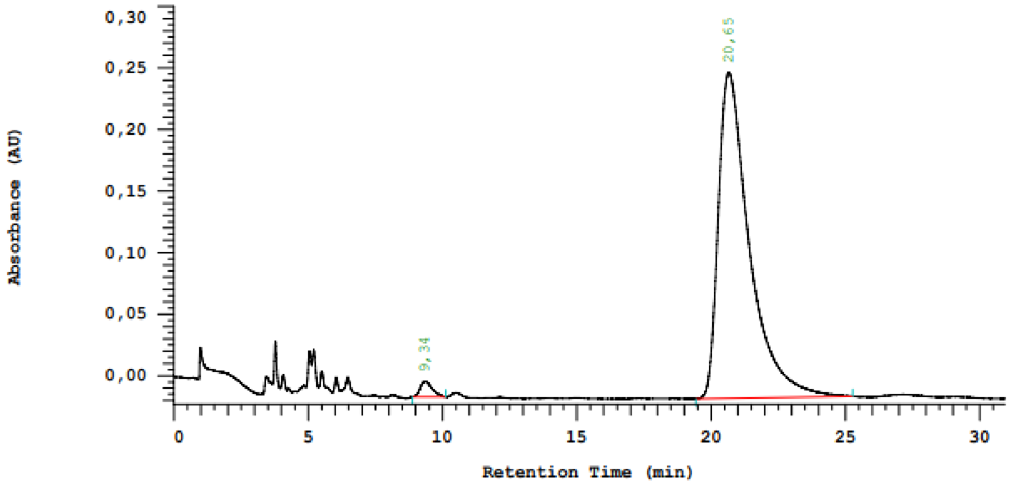

No.	RT	Area	Area %	Name
1	9,34	189795	1,756	
2	20,65	10615550	98,244	
		10805345	100,000	

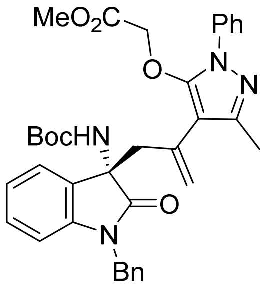

6

Sample Name: LC-584 ODH 9010 1mL Vial Number: 1

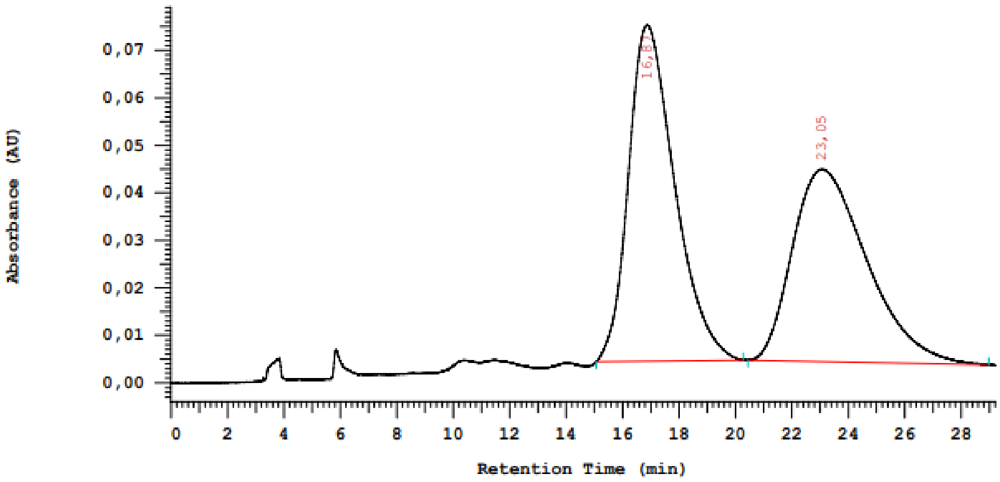

No.	RT	Area	Area %	Name
1	16,87	4018320	52,034	
2	23,05	3704150	47,966	enant. (+)
		7722470	100,000	

Sample Name: LC-581 ODH 9010 1mL Vial Number: 1

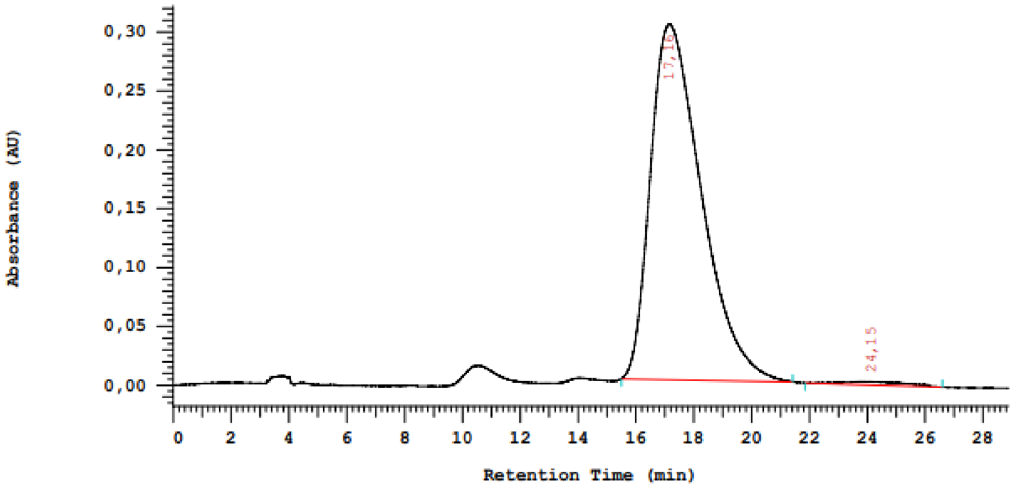

No.	RT	Area	Area %	Name
1	17,16	18474620	98,825	
2	24,15	219640	1,175	enant. (+)
		18694260	100,000	
